# Supplementary material for: Using cross-species co-expression to predict metabolic interactions in microbiomes
Source: mSystems. 2025 Dec 9;11(1):e01321-25. doi: 10.1128/msystems.01321-25 (PMC12817932; doi:10.1128/msystems.01321-25)
Supplement: Supplemental Figures — Figures S1 to S12. [file msystems.01321-25-s0001.pdf]

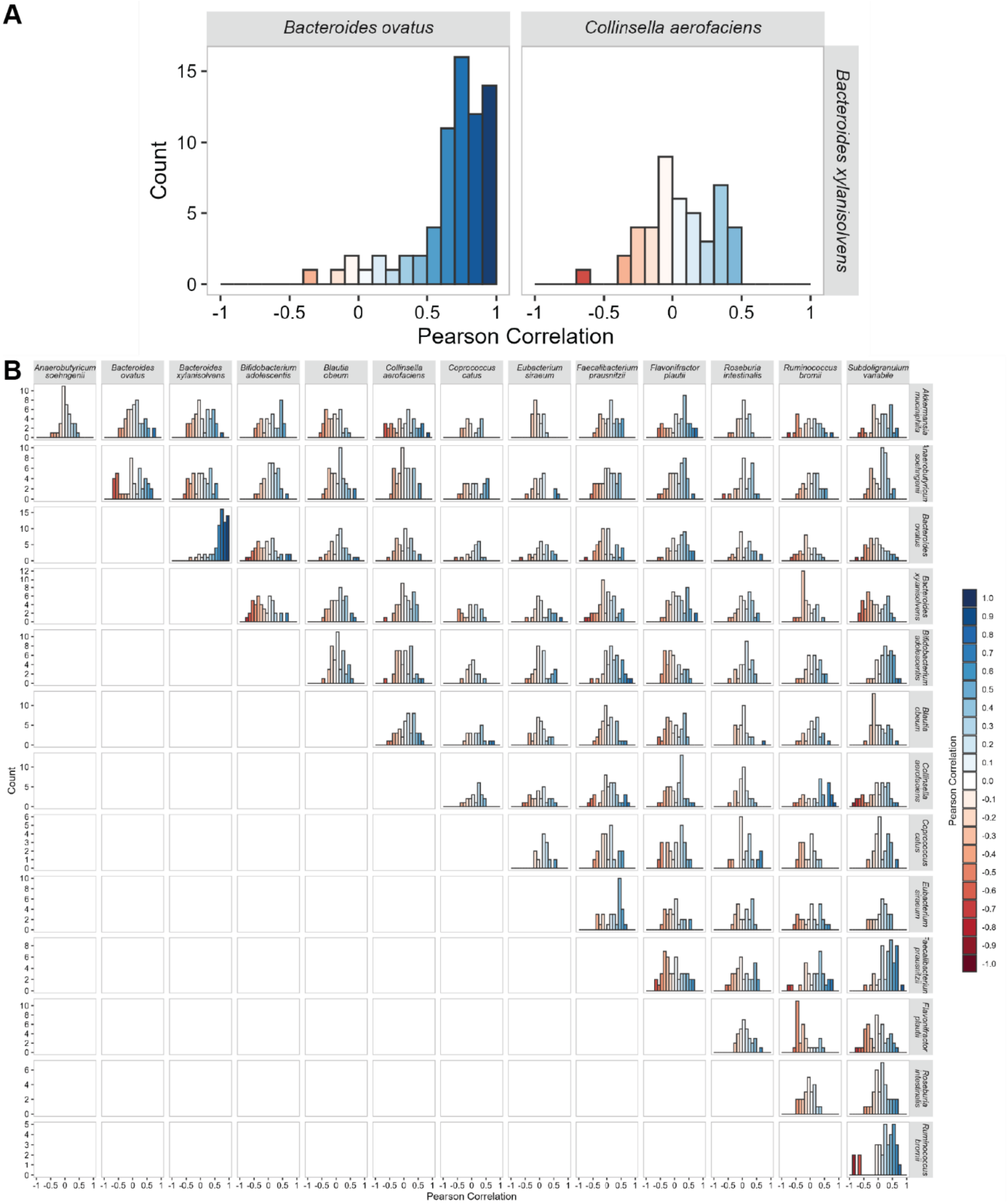

Fig S1 *Distributions of Pearson correlation values quantifying the co-expression of corresponding GMMs from different MDb-MM species. By correlating identical GMMs, we investigate whether redundant functions of the community are generally expressed simultaneously by different species. A) GMM co-expression of Bacteroides xylanisolvens vs Bacteroides ovatus and Collinsella aerofaciens. The Bacteroides pair co-expresses many of the GMMs that occur in both species, the pair B. xylanisolvens and C. aerofaciens is used as a representative example for the relation that is observed for most pairs of species, notably fewer strong correlations exist in comparison to the Bacteroides pair. B) Full matrix with all combinations of the MDb-MM species, showing co-expression of corresponding GMMs for each pair.*

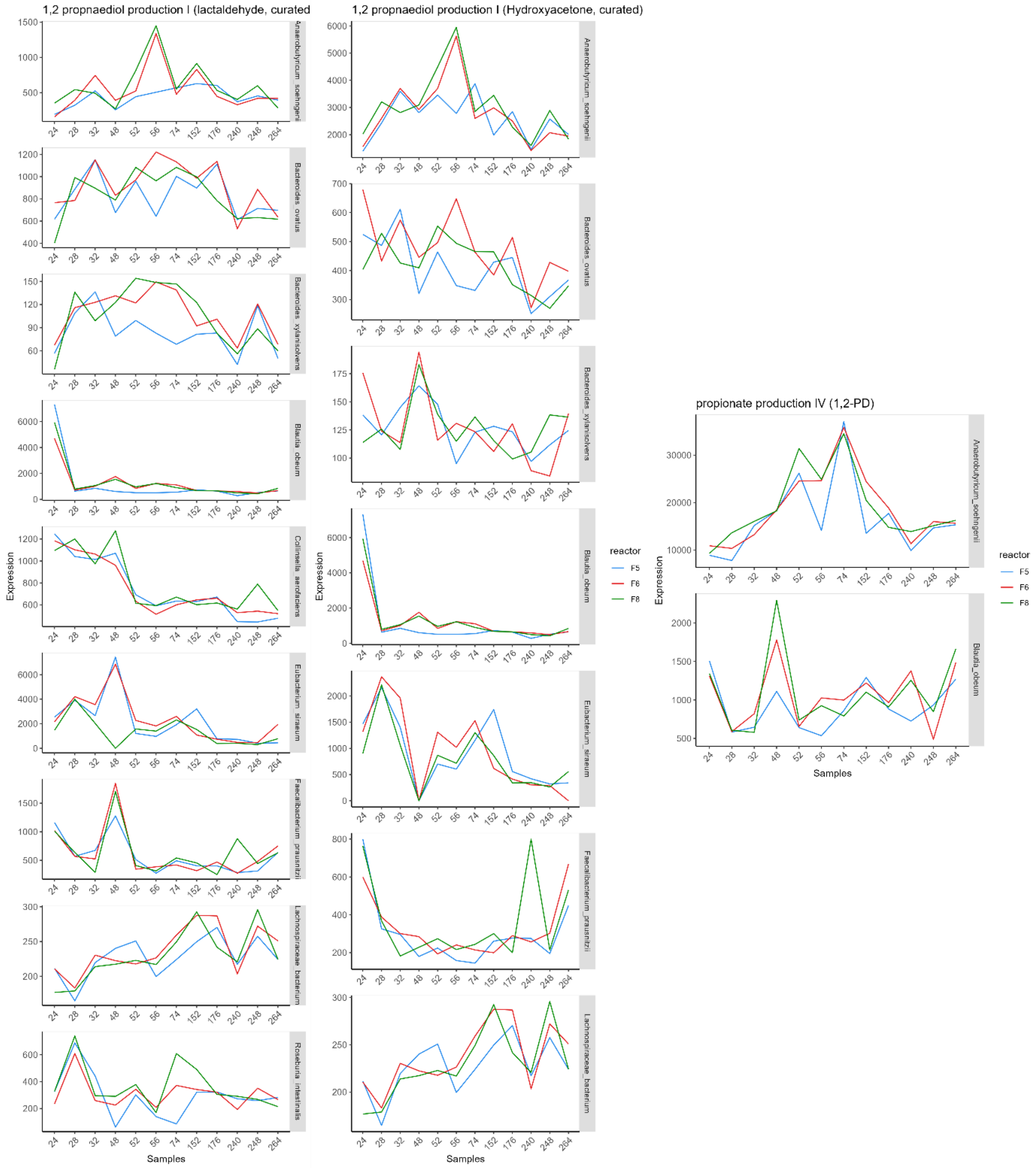

Fig S2 *Expression profiles of GMMs related to 1,2-propanediol production (left) and its consumption (right). Columns show different GMMs, each panel depicts expression (TMM normalized counts) of a single MDb-MM species over time (hours) for 3 parallel bioreactors. Notable is the peak in 1,2-propanediol consumption for Blautia obeum at 48h, which matches well with peaks in production of Faecalibacterium prausnitzii and Bacteroides xylanisolvens at the same timepoint.*

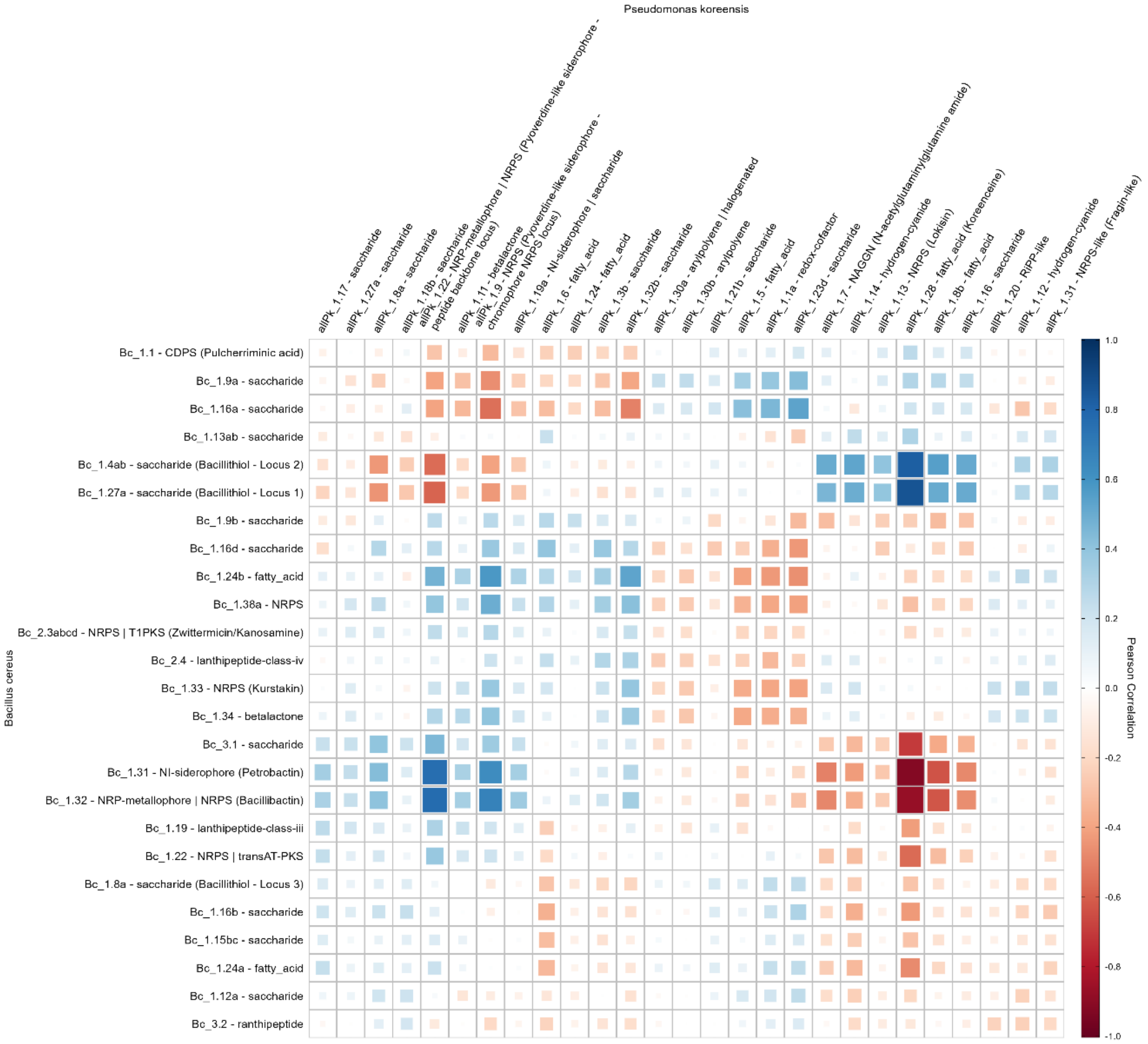

***Fig S3** Co-expression of Bacillus cereus and Pseudomonas koreensis BGCs, as shown in the main text. Shown here for a complete representation in the supplementals of the BGC co-expression results for all THOR species pairs.*

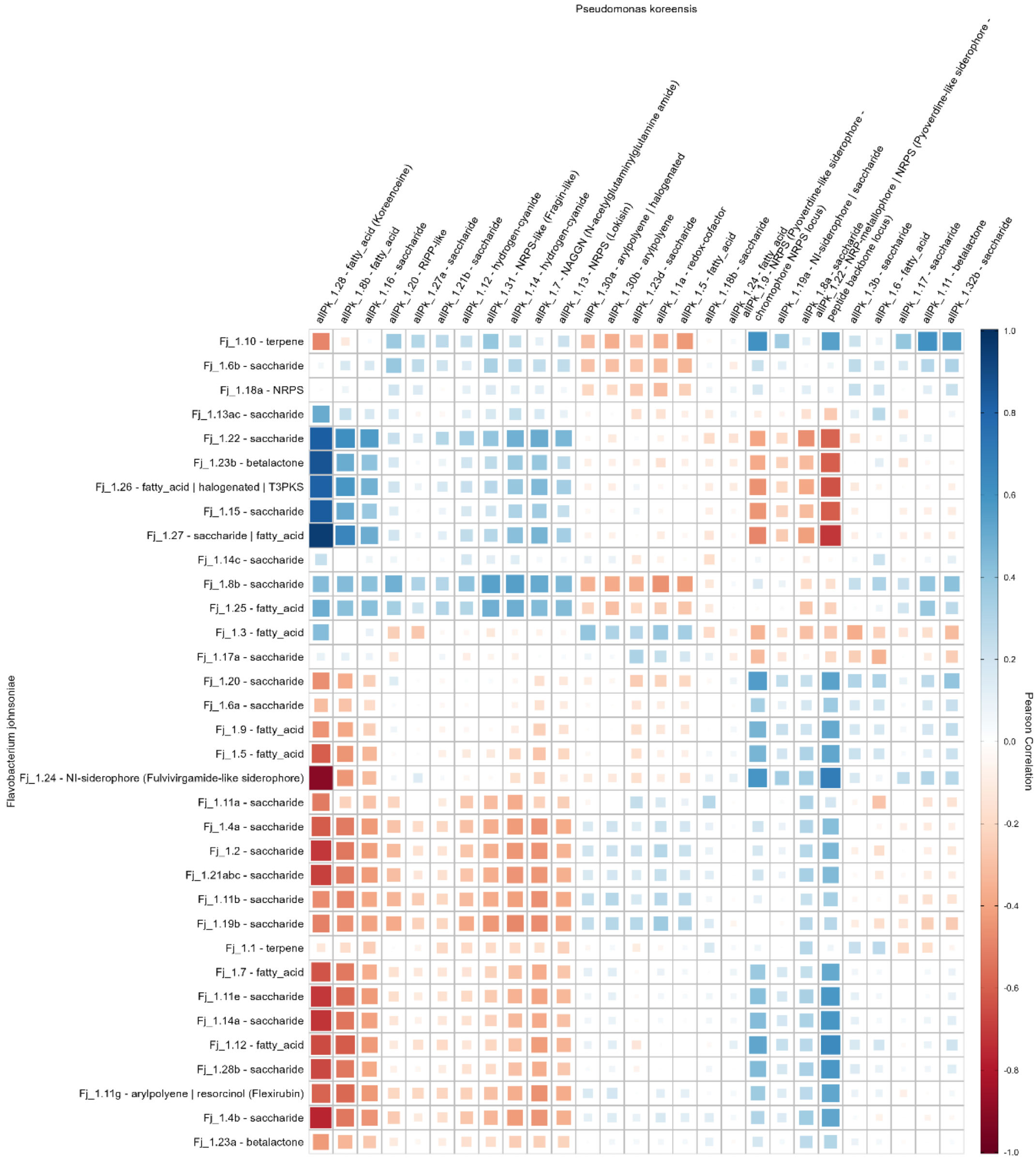

Fig S4 *Co-expression of Flavobacterium johnsoniae and Pseudomonas koreensis BGCs.*

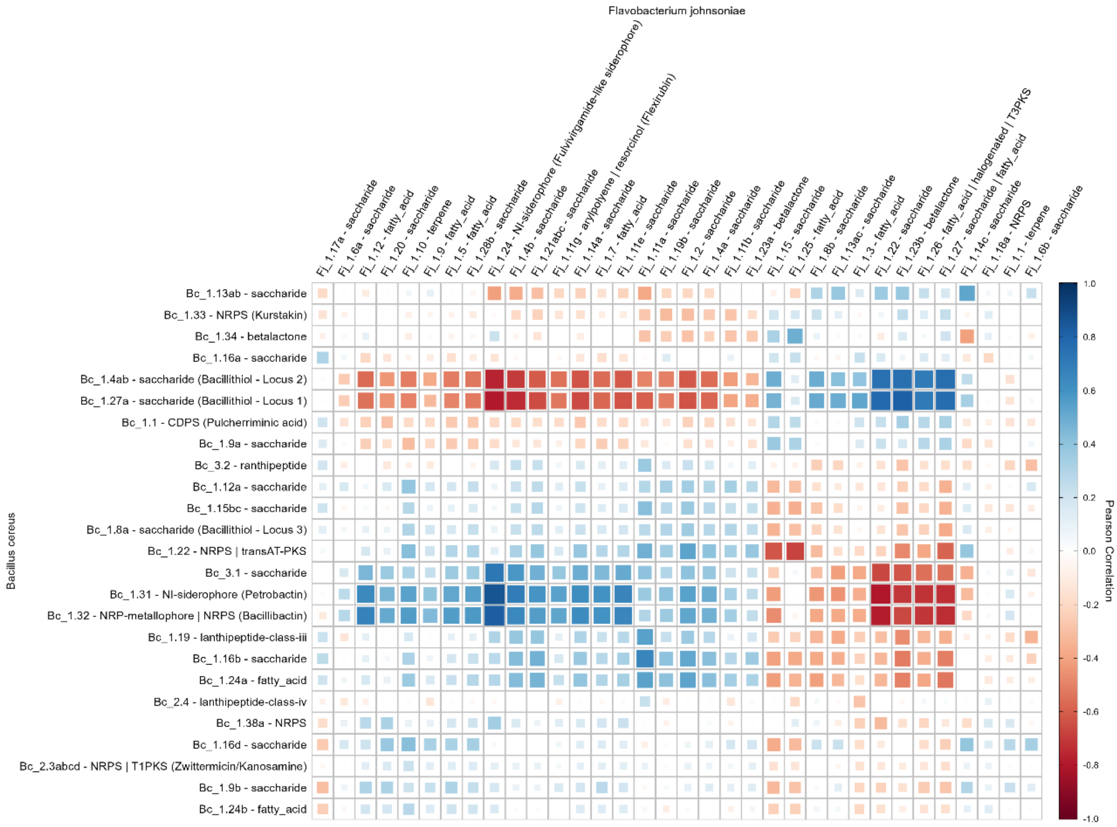

Fig S5 *Co-expression of Flavobacterium johnsoniae and Bacillus cereus BGCs.*

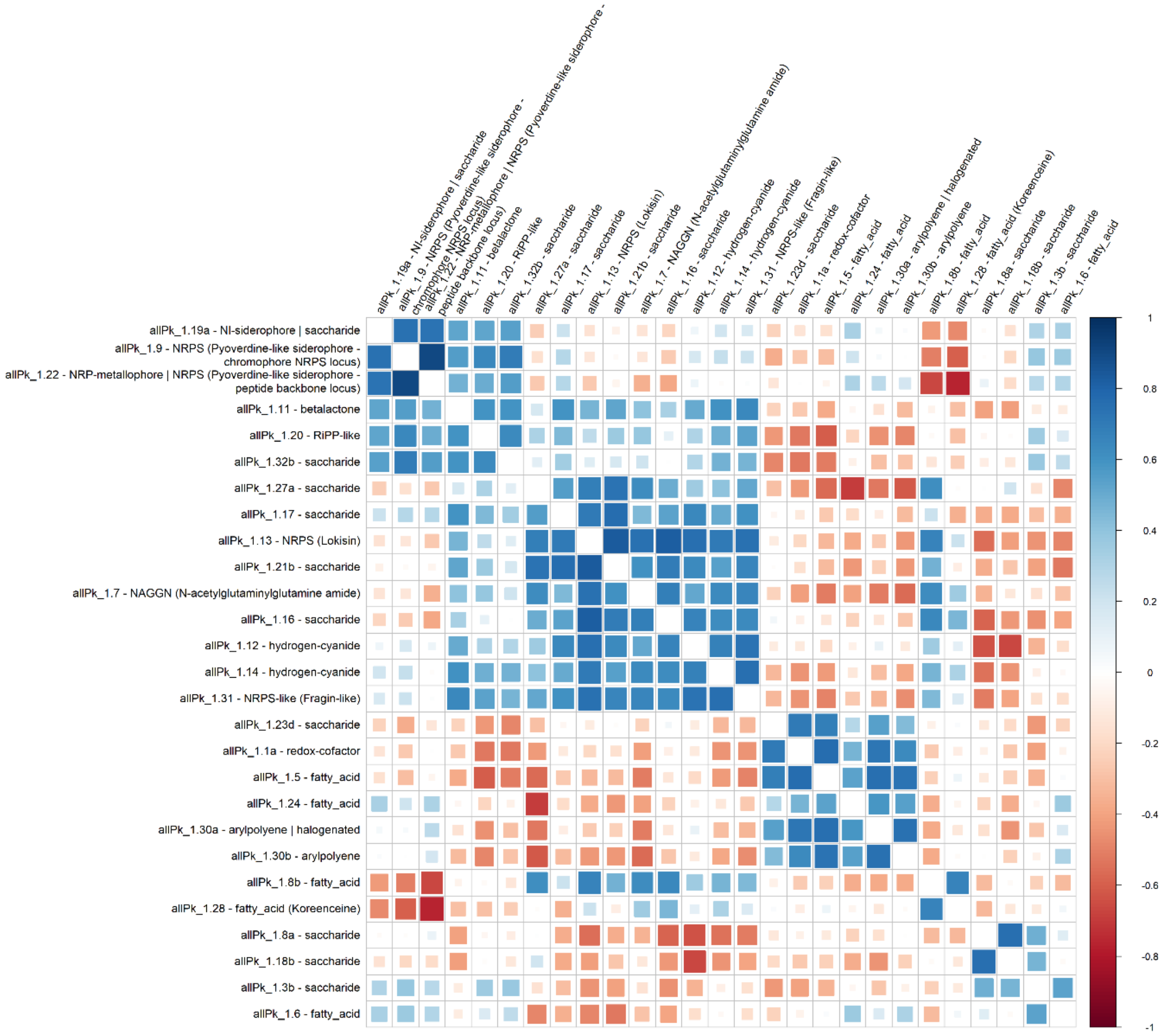

Fig S6 *Co-expression of Pseudomonas koreensis BGCs.*

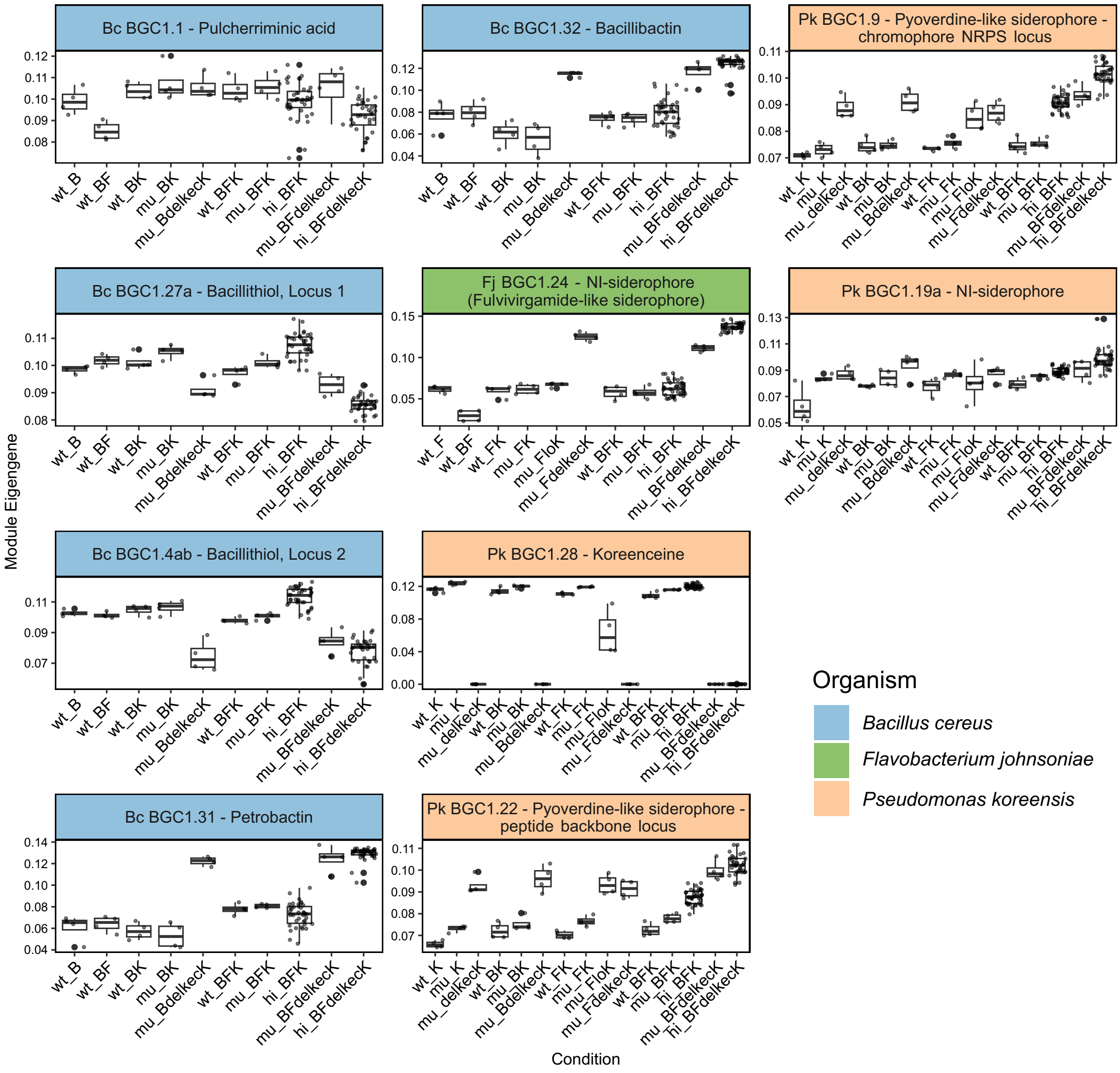

Fig S7 *Expression profiles of BGCs highlighted in the main text. The x-axis labels describe different community compositions, B = B. cereus, F = F. johnsoniae, K = P. koreensis, while prefixes describe different experiments, wt = wild type, no mutants were included in this round of experiments, mu = mutant, this experimental round introduced some conditions with P. koreensis koreenceine deletion mutants (delkecK) and a low inoculum condition (loK), hi = high replicate, this experimental round was characterized by conditions with very high replicate numbers. BGC expression is visualized with the cluster's eigengene, which summarizes the expression of the BGC's genes by taking the first principal component of their scaled and log-transformed counts. Note that co-expression was calculated across samples and not conditions as shown here.*

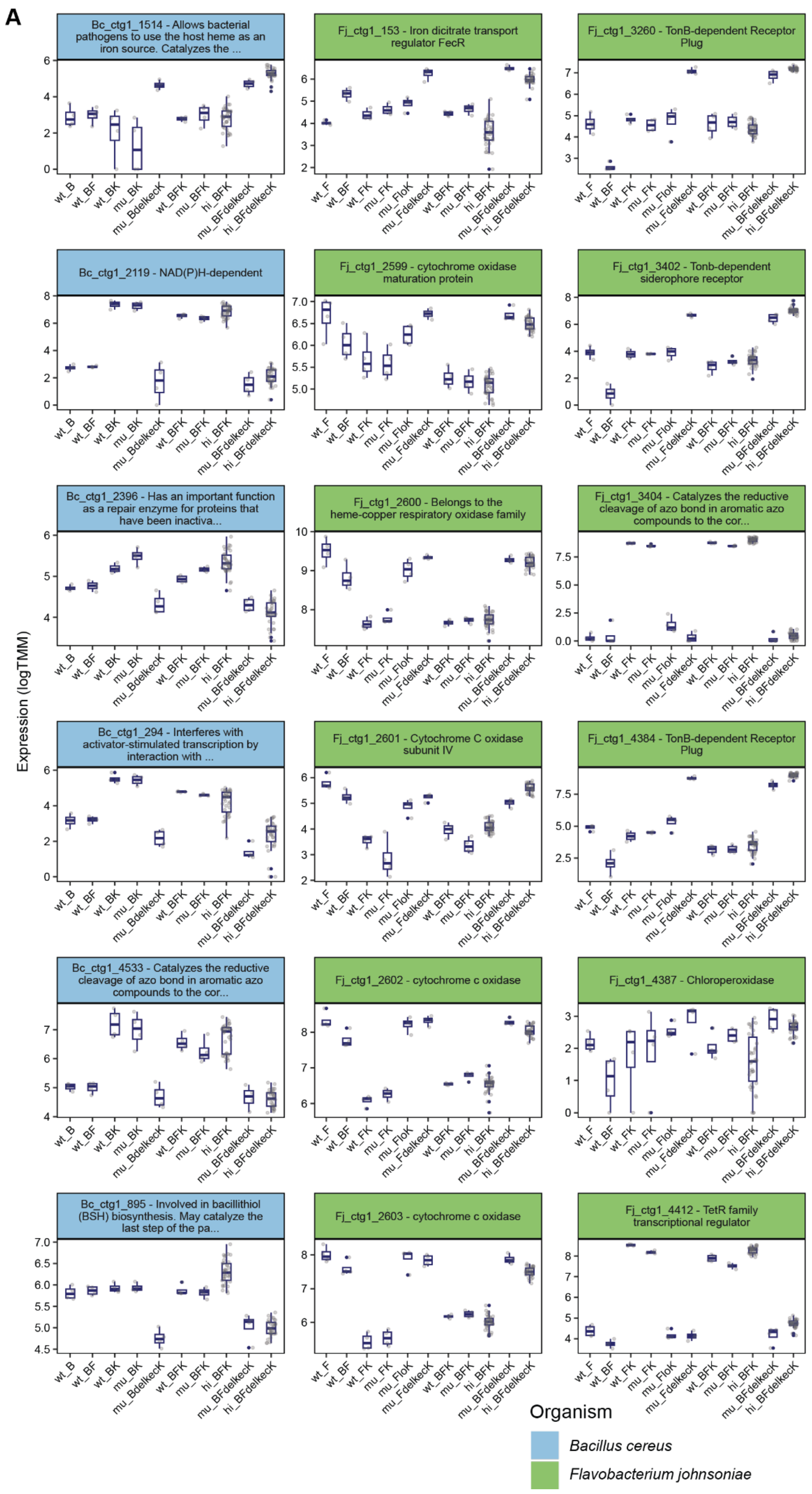
A
Bc_ctg1_1514 - Allows bacterial pathogens to use the host heme as an iron source. Catalyzes the ...
Fj_ctg1_153 - Iron dicitrate transport regulator FecR
Fj_ctg1_3260 - TonB-dependent Receptor Plug
Bc_ctg1_2119 - NAD(P)H-dependent
Fj_ctg1_2599 - cytochrome oxidase maturation protein
Fj_ctg1_3402 - Tonb-dependent siderophore receptor
Bc_ctg1_2396 - Has an important function as a repair enzyme for proteins that have been inactiva...
Fj_ctg1_2600 - Belongs to the heme-copper respiratory oxidase family
Fj_ctg1_3404 - Catalyzes the reductive cleavage of azo bond in aromatic azo compounds to the cor...
Bc_ctg1_294 - Interferes with activator-stimulated transcription by interaction with ...
Fj_ctg1_2601 - Cytochrome C oxidase subunit IV
Fj_ctg1_4384 - TonB-dependent Receptor Plug
Bc_ctg1_4533 - Catalyzes the reductive cleavage of azo bond in aromatic azo compounds to the cor...
Fj_ctg1_2602 - cytochrome c oxidase
Fj_ctg1_4387 - Chloroperoxidase
Bc_ctg1_895 - Involved in bacillithiol (BSH) biosynthesis. May catalyze the last step of the pa...
Fj_ctg1_2603 - cytochrome c oxidase
Fj_ctg1_4412 - TetR family transcriptional regulator
Expression (logTMM)
wt_B wt_BF wt_BK mu_BK mu_Bdelkeck wt_BFK mu_BFK hi_BFK mu_BFdelkecK hi_BFdelkecK
wt_F wt_BF wt_FK mu_FK mu_FloK mu_FdelkecK wt_BFK mu_BFK hi_BFK mu_BFdelkecK hi_BFdelkecK
Organism
Bacillus cereus
Flavobacterium johnsoniae

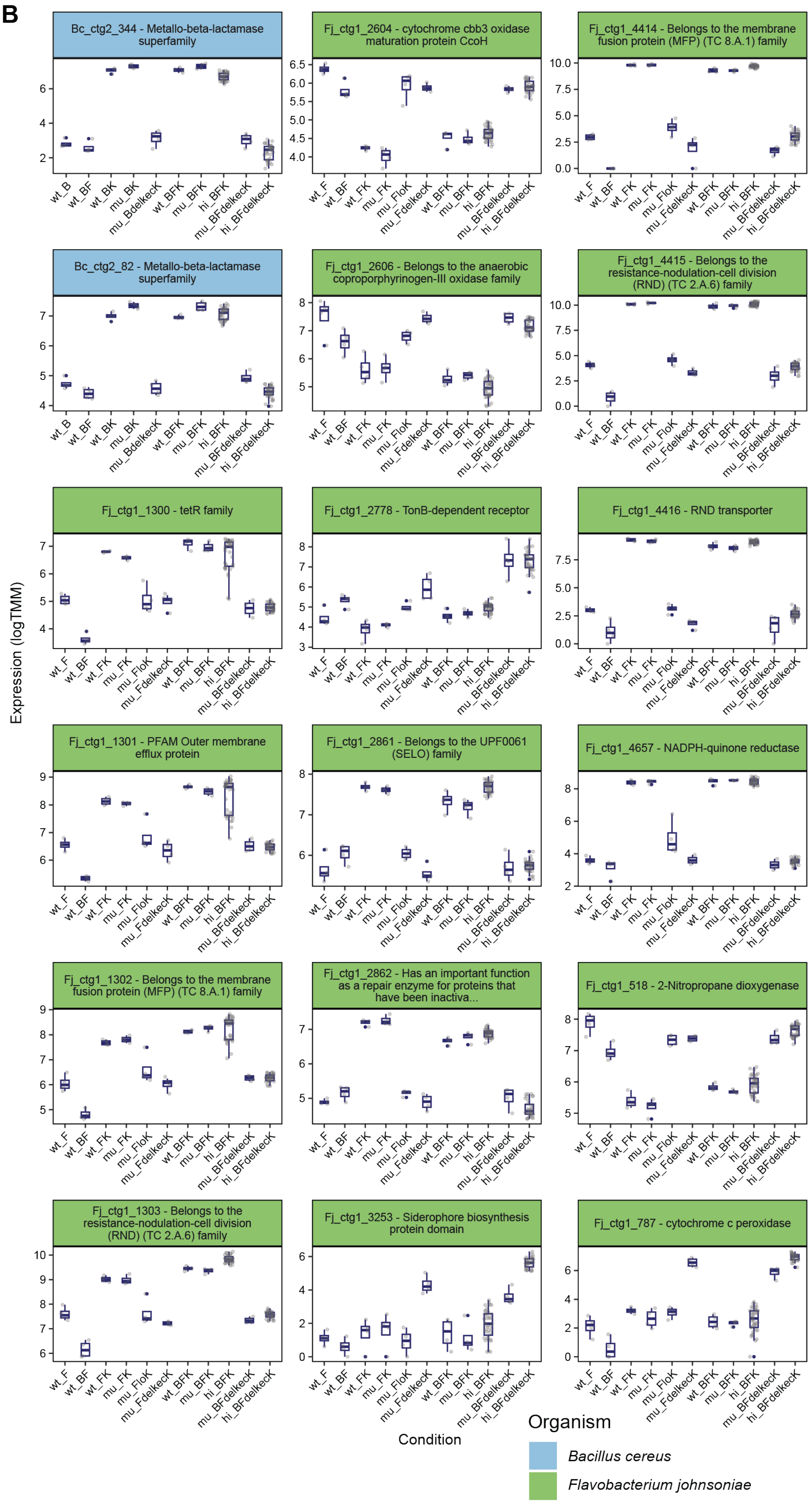
B
Bc_ctg2_344 - Metallo-beta-lactamase superfamily
Fj_ctg1_2604 - cytochrome cbb3 oxidase maturation protein CcoH
Fj_ctg1_4414 - Belongs to the membrane fusion protein (MFP) (TC 8.A.1) family
Bc_ctg2_82 - Metallo-beta-lactamase superfamily
Fj_ctg1_2606 - Belongs to the anaerobic coproporphyrinogen-III oxidase family
Fj_ctg1_4415 - Belongs to the resistance-nodulation-cell division (RND) (TC 2.A.6) family
Fj_ctg1_1300 - tetR family
Fj_ctg1_2778 - TonB-dependent receptor
Fj_ctg1_4416 - RND transporter
Fj_ctg1_1301 - PFAM Outer membrane efflux protein
Fj_ctg1_2861 - Belongs to the UPF0061 (SELO) family
Fj_ctg1_4657 - NADPH-quinone reductase
Fj_ctg1_1302 - Belongs to the membrane fusion protein (MFP) (TC 8.A.1) family
Fj_ctg1_2862 - Has an important function as a repair enzyme for proteins that have been inactiva...
Fj_ctg1_518 - 2-Nitropropane dioxygenase
Fj_ctg1_1303 - Belongs to the resistance-nodulation-cell division (RND) (TC 2.A.6) family
Fj_ctg1_3253 - Siderophore biosynthesis protein domain
Fj_ctg1_787 - cytochrome c peroxidase
Expression (logTMM)
Condition
wt_B, wt_BF, wt_BK, mu_BK, mu_Bdelkeck, wt_BFK, mu_BFK, hi_BFK, mu_BFdelkecK, hi_BFdelkecK
wt_F, wt_BF, wt_FK, mu_FK, mu_FloK, mu_FdelkecK, wt_BFK, mu_BFK, hi_BFK, mu_BFdelkecK, hi_BFdelkecK
Organism
Bacillus cereus
Flavobacterium johnsoniae

Fig S8 *Expression profiles of genes that are strongly co-expressed with the koreenceine BGC of Pseudomonas koreensis. The x-axis labels describe different community compositions, B = B. cereus, F = F. johnsoniae, K = P. koreensis, while prefixes describe different experiments, wt = wild type, no mutants were included in this round of experiments, mu = mutant, this experimental round introduced some conditions with P. koreensis koreenceine deletion mutants (delkecK) and a low inoculum condition (loK), hi = high replicate, this experimental round was characterized by conditions with very high replicate numbers.*

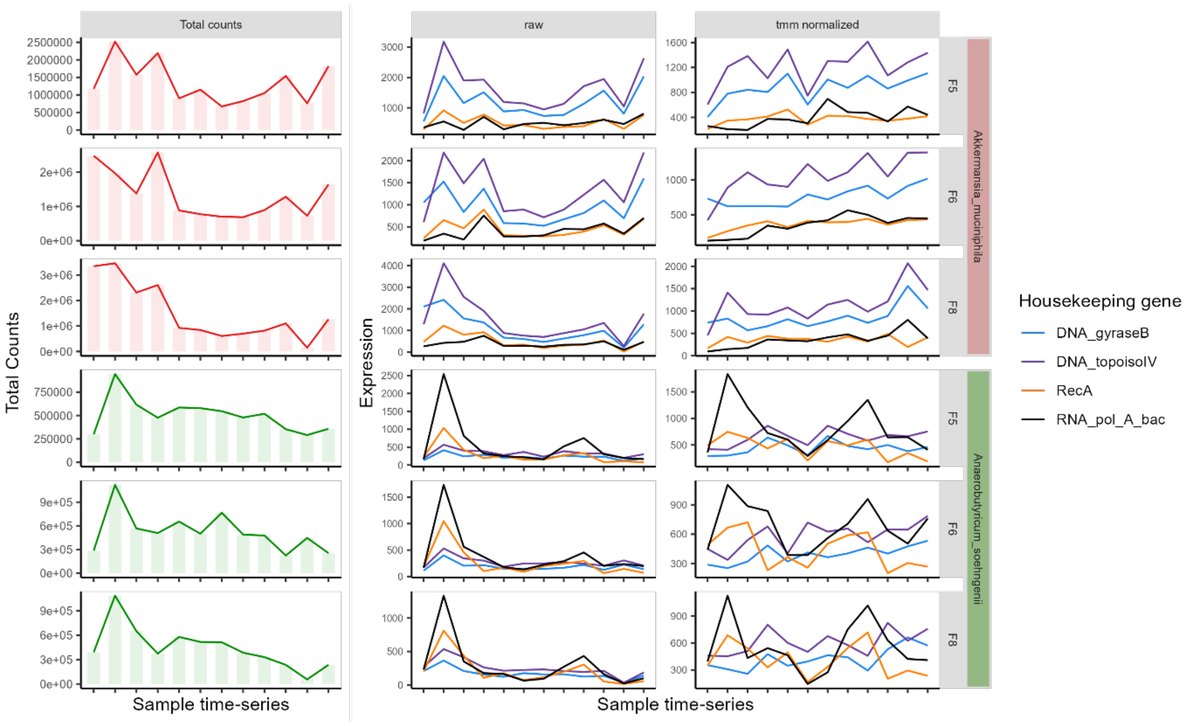

Fig S9 *Total RNA-seq counts for MDb-MM species Akkermansia muciniphila and Anaerobutyricum soehngenii, and expression profiles of a selection of their housekeeping genes. Before normalization, the raw gene expression values of both organism's housekeeping genes are highly correlated with the total counts, while after TMM normalization this relationship is corrected for.*

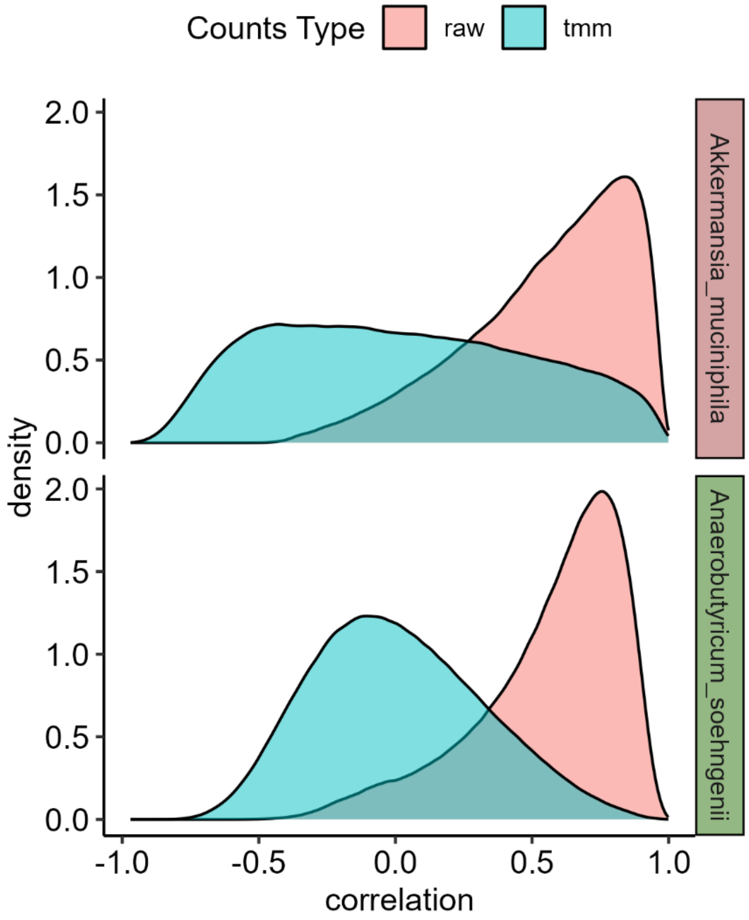

Fig S10 *Distribution of Pearson correlation values for all vs all gene co-expression of Akkermansia muciniphila and Anaerobutyricum soehngenii, before and after TMM normalization. Before normalization, all genes correlate strongly due to the differences in library sizes, as show in* ***Fig S9.***

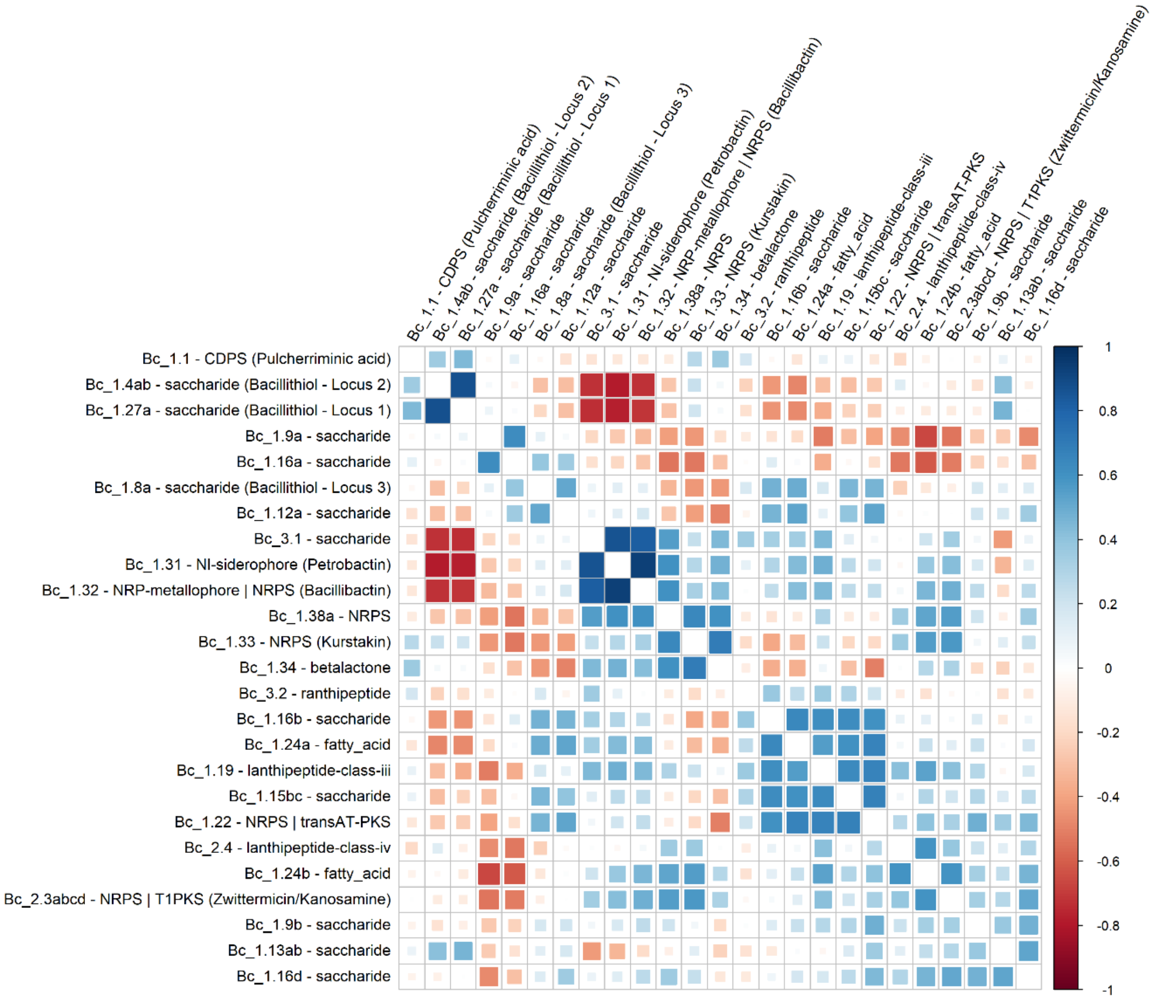

***Fig S11** Co-expression of Bacillus cereus BGCs.*

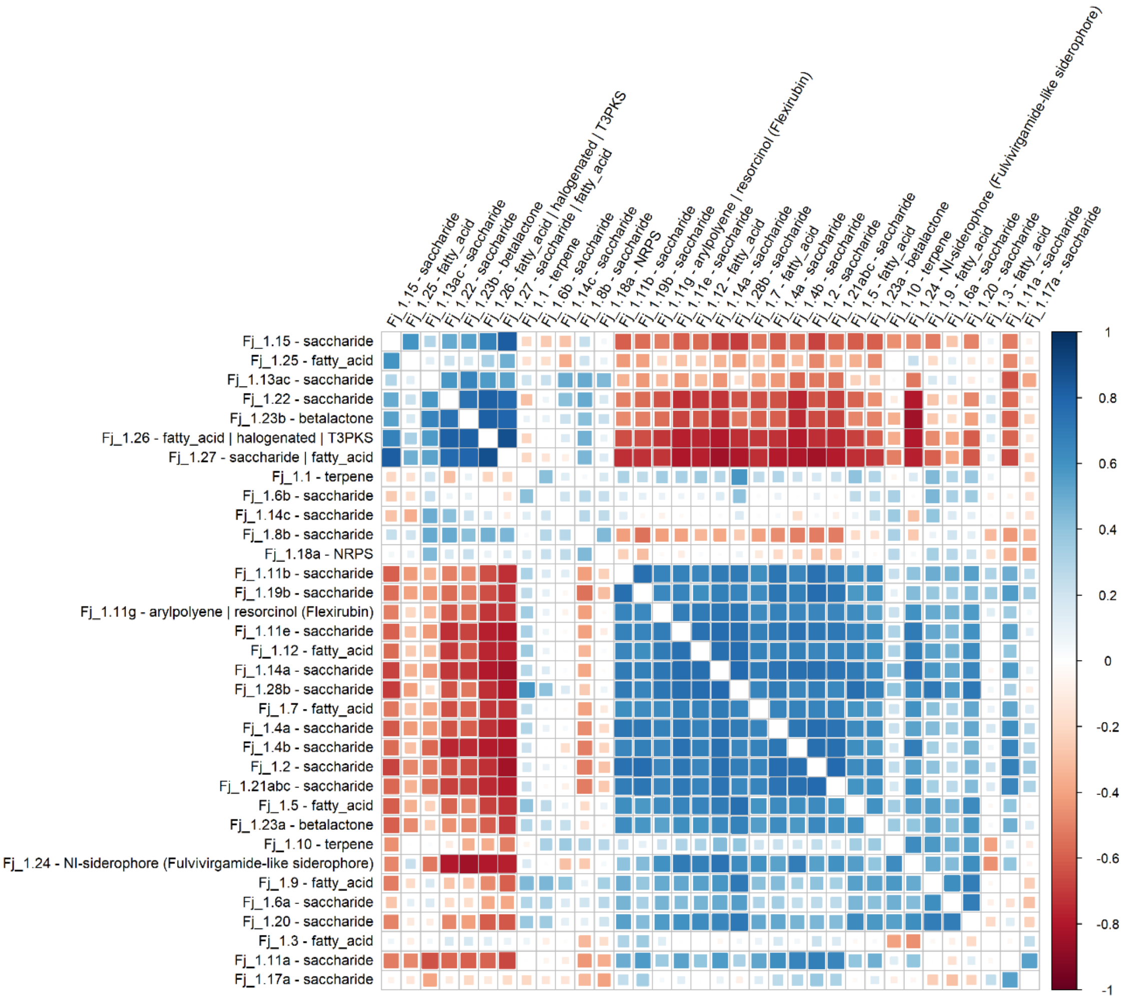

***Fig S12** Co-expression of Flavobacterium johnsoniae BGCs.*
