# Supplementary material for: Using cross-species co-expression to predict metabolic interactions in microbiomes
Source: mSystems. 2025 Dec 9;11(1):e01321-25. doi: 10.1128/msystems.01321-25 (PMC12817932; doi:10.1128/msystems.01321-25)
Supplement: Supplemental File 1 — An overview of the genes that make up the final, refined BGCs investigated in the co-expression analyses. The heatmaps show the co-expression (Pearson correlation) between all the genes of the BGC, 'x' is shown in a cell if a gene was removed from the analysis due to low expression. Letters on the diagonal correspond to the roles that antiSMASH has assigned to the genes of this BGC, B = core biosynthetic gene, A = Additional biosynthetic gene, T = transport-related gene, R = regulatory gene. Black squares around the cells indicate the genes that compose the refined BGC. In the title of the plots, abbreviations show the BGC's organism, Bc = Bacillus cereus, Fj = Flavobacterium johnsoniae, Pk = Pseudomonas koreensis. If two columns of heatmaps are shown, the black square on the left heatmap will indicate what genes were finally used for the BGC, while the black square on the right heatmap indicates which genes are known to be involved with the synthesis of the metabolite based on literature. [file msystems.01321-25-s0003.pdf]

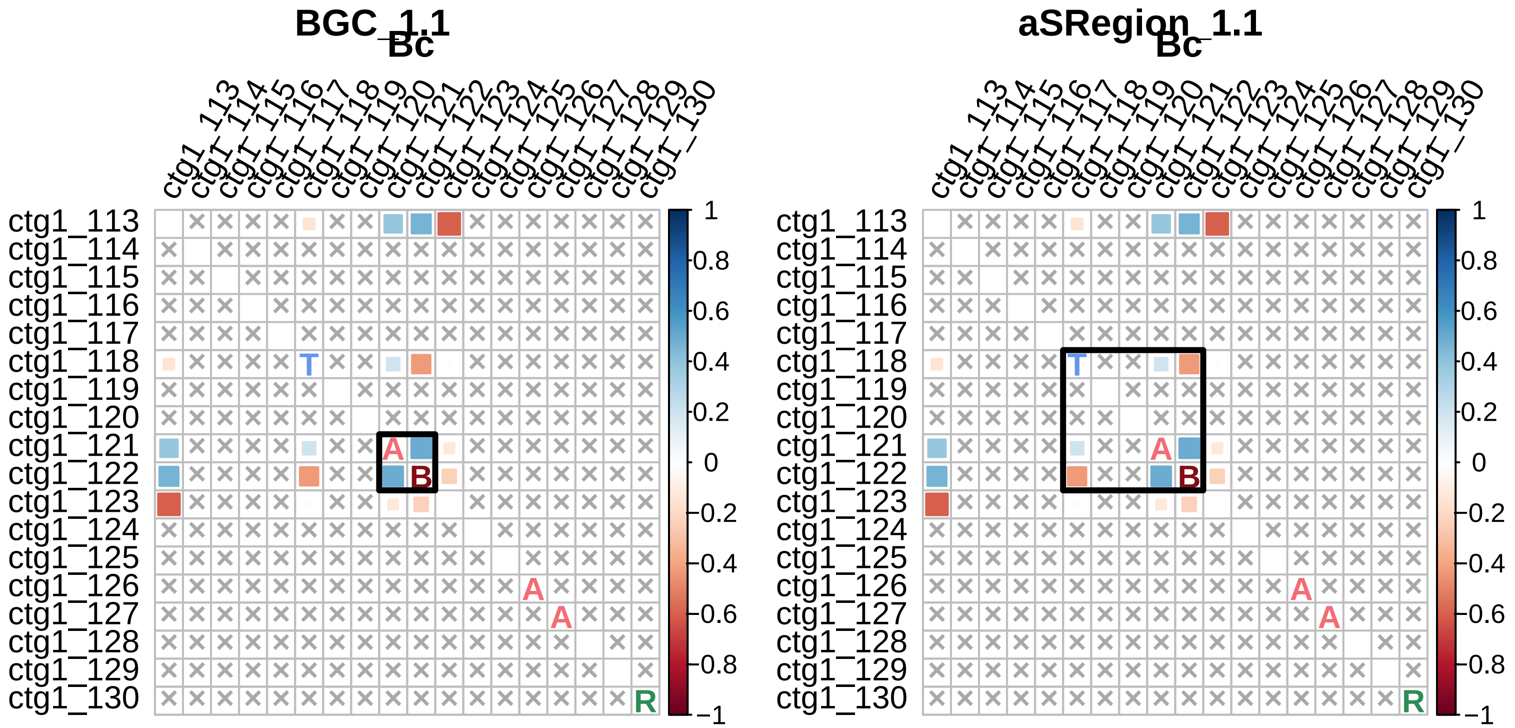

BGC_1.1
Bc
aSRegion_1.1
Bc
ctg1_113
ctg1_114
ctg1_115
ctg1_116
ctg1_117
ctg1_118
ctg1_119
ctg1_120
ctg1_121
ctg1_122
ctg1_123
ctg1_124
ctg1_125
ctg1_126
ctg1_127
ctg1_128
ctg1_129
ctg1_130
T
A
B
R
1
0.8
0.6
0.4
0.2
0
−0.2
−0.4
−0.6
−0.8
−1

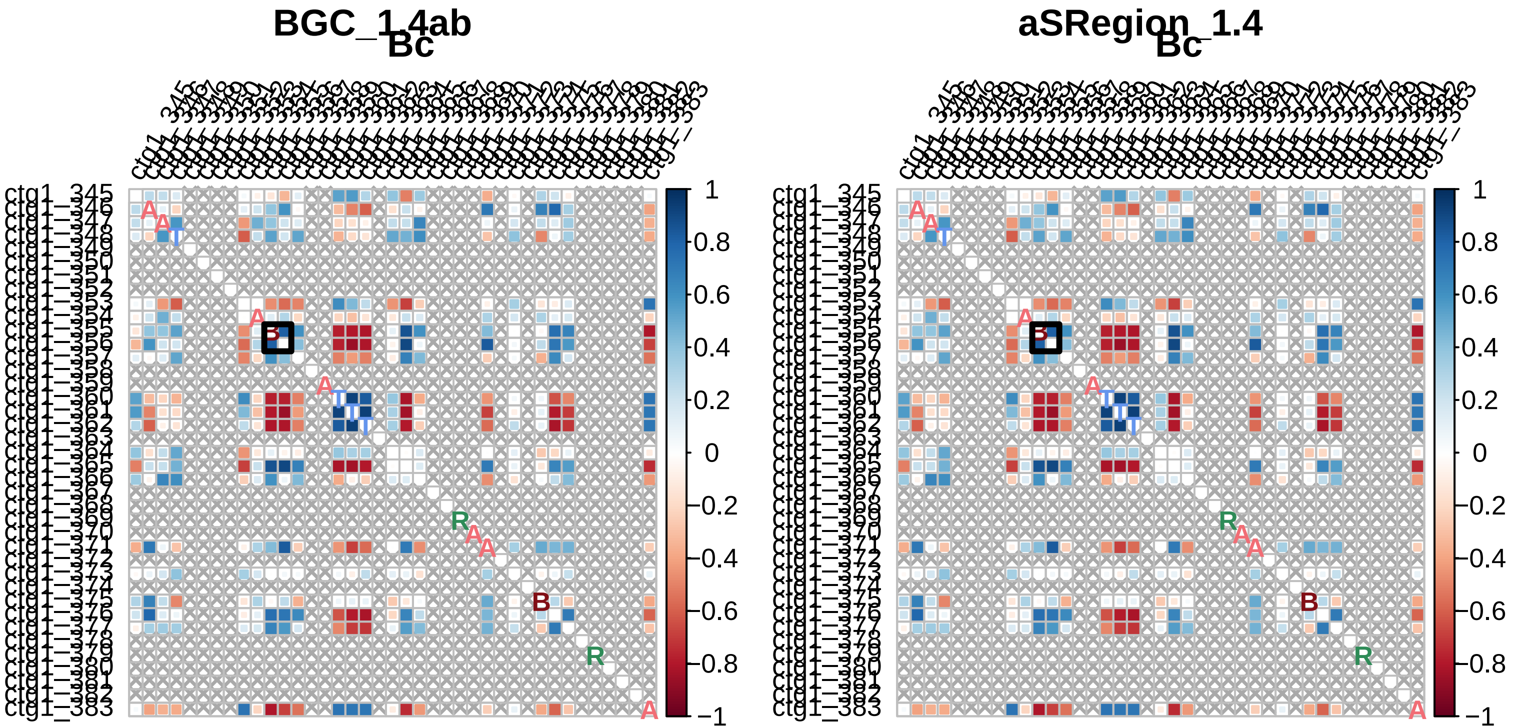

BGC_1.4ab
Bc
aSRegion_1.4
Bc
ctg1_345
ctg1_383
1
0.8
0.6
0.4
0.2
0
−0.2
−0.4
−0.6
−0.8
−1

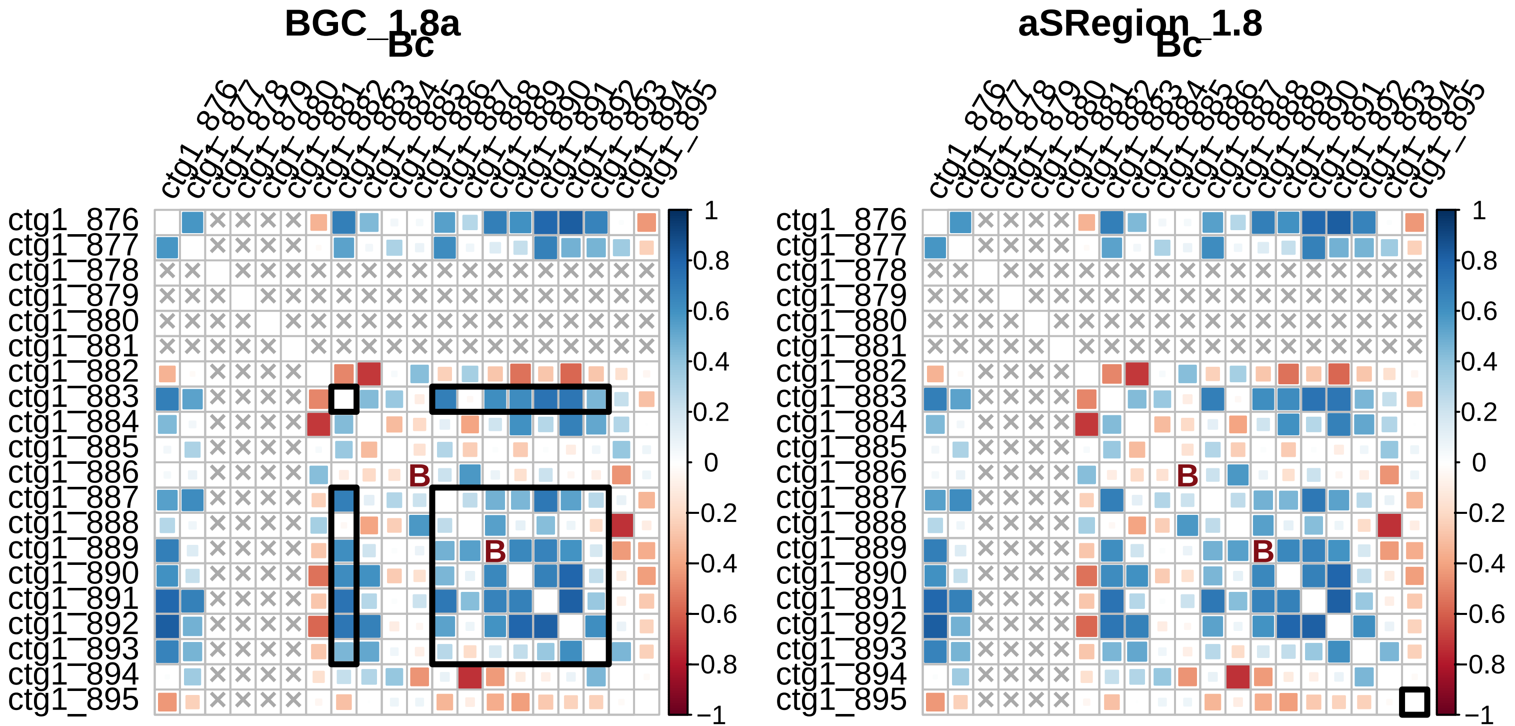

BGC_1.8a
Bc
aSRegion_1.8
Bc
ctg1_876
ctg1_877
ctg1_878
ctg1_879
ctg1_880
ctg1_881
ctg1_882
ctg1_883
ctg1_884
ctg1_885
ctg1_886
ctg1_887
ctg1_888
ctg1_889
ctg1_890
ctg1_891
ctg1_892
ctg1_893
ctg1_894
ctg1_895
B
B
1
0.8
0.6
0.4
0.2
0
−0.2
−0.4
−0.6
−0.8
−1

BGC_1.9a

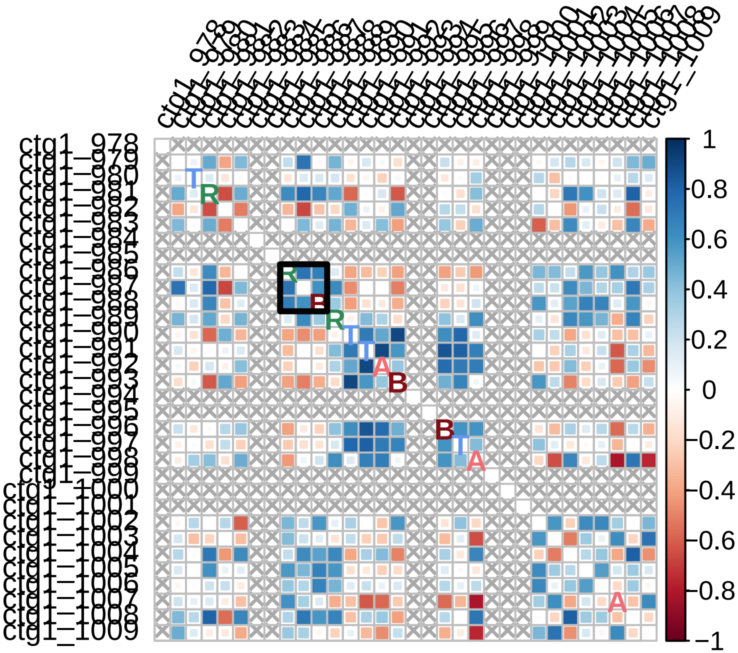

BGC_1.9b

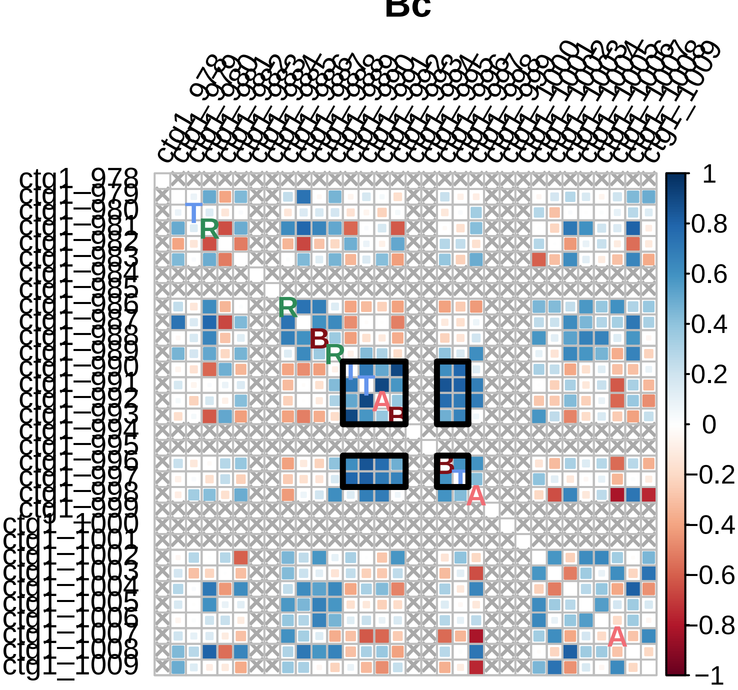

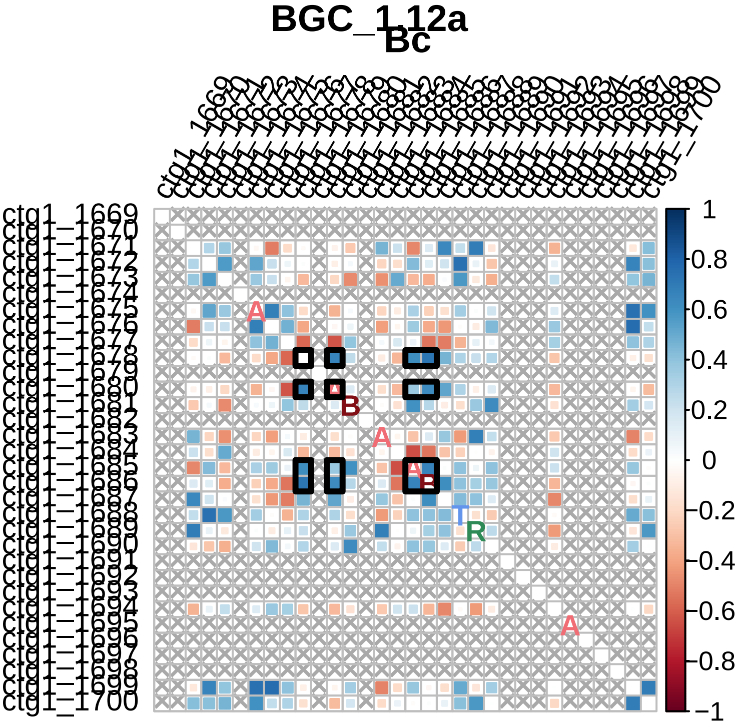

BGC_1.12a
Bc
ctg1_1669
ctg1_1670
ctg1_1671
ctg1_1672
ctg1_1673
ctg1_1674
ctg1_1675
ctg1_1676
ctg1_1677
ctg1_1678
ctg1_1679
ctg1_1680
ctg1_1681
ctg1_1682
ctg1_1683
ctg1_1684
ctg1_1685
ctg1_1686
ctg1_1687
ctg1_1688
ctg1_1689
ctg1_1690
ctg1_1691
ctg1_1692
ctg1_1693
ctg1_1694
ctg1_1695
ctg1_1696
ctg1_1697
ctg1_1698
ctg1_1699
ctg1_1700
A
B
T
R
1
0.8
0.6
0.4
0.2
0
−0.2
−0.4
−0.6
−0.8
−1

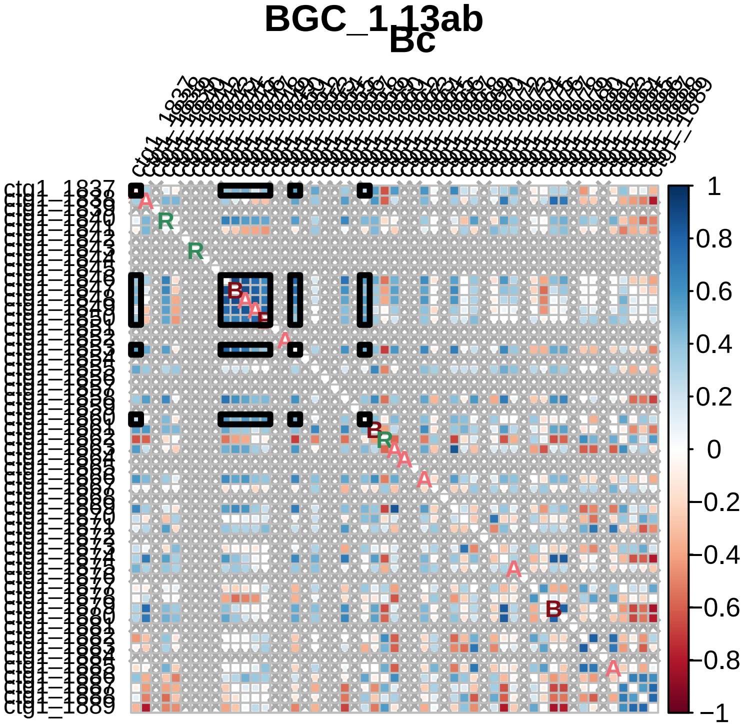
BGC_1_13ab
Bc
1
0.8
0.6
0.4
0.2
0
−0.2
−0.4
−0.6
−0.8
−1

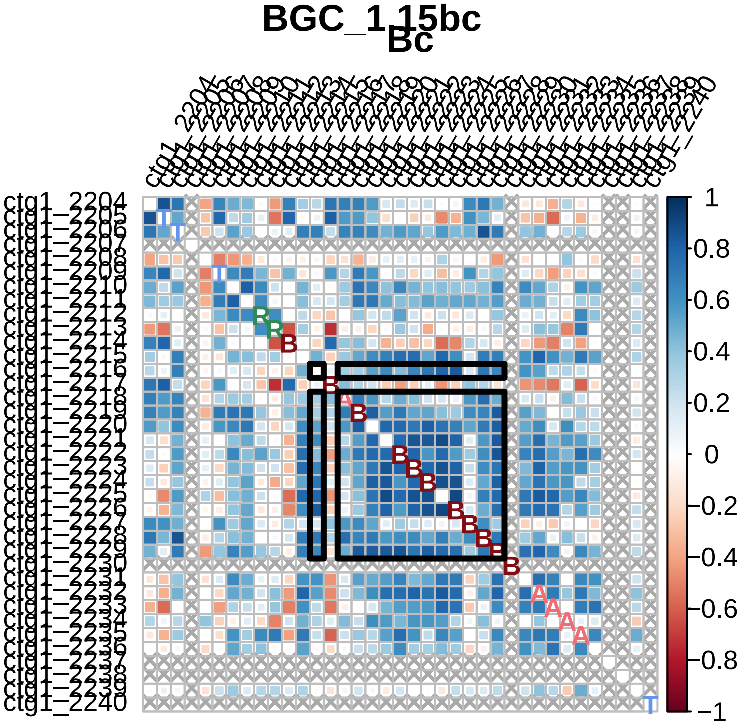
BGC_1 15bc
Bc
ctg1_2204
ctg1_2240
1
0.8
0.6
0.4
0.2
0
−0.2
−0.4
−0.6
−0.8
−1

BGC_1.16a

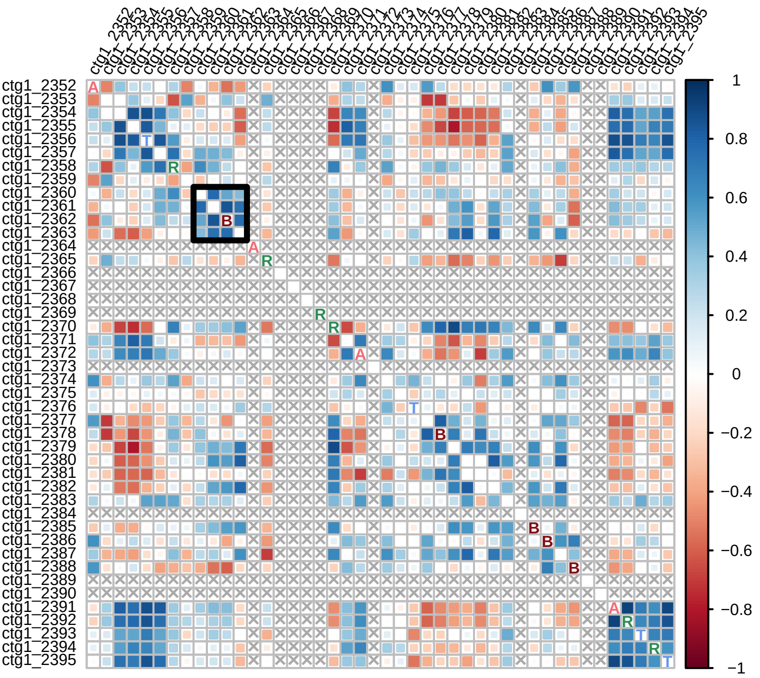

BGC_1.16b

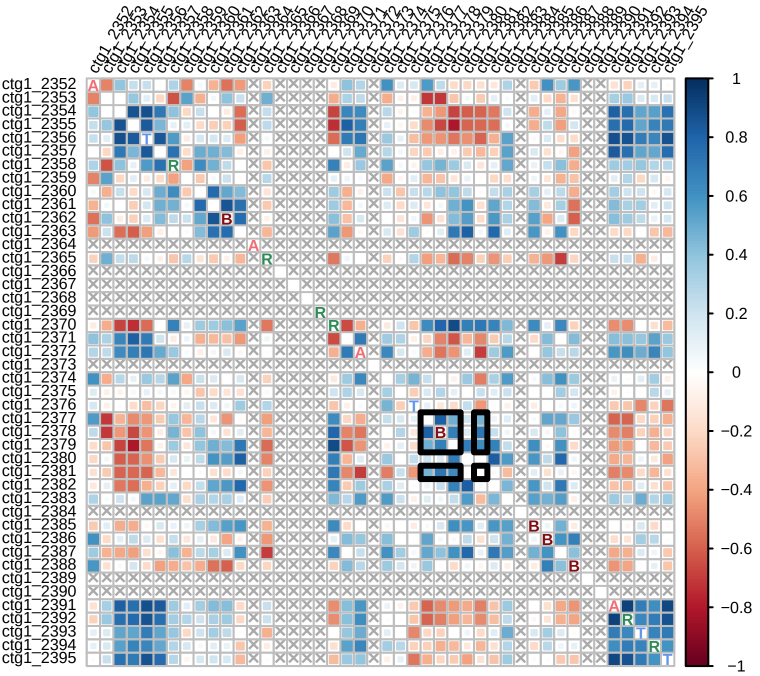

BGC_1.16d
Bc

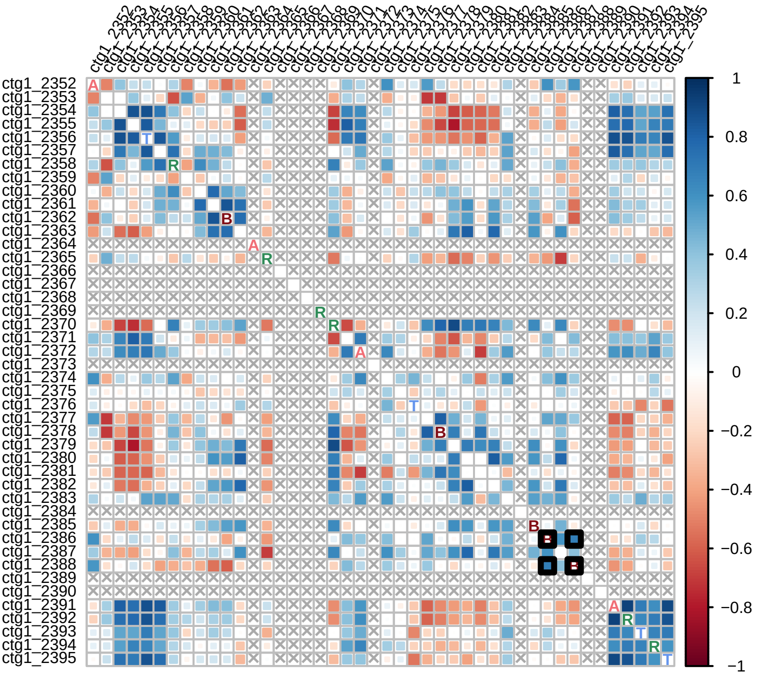

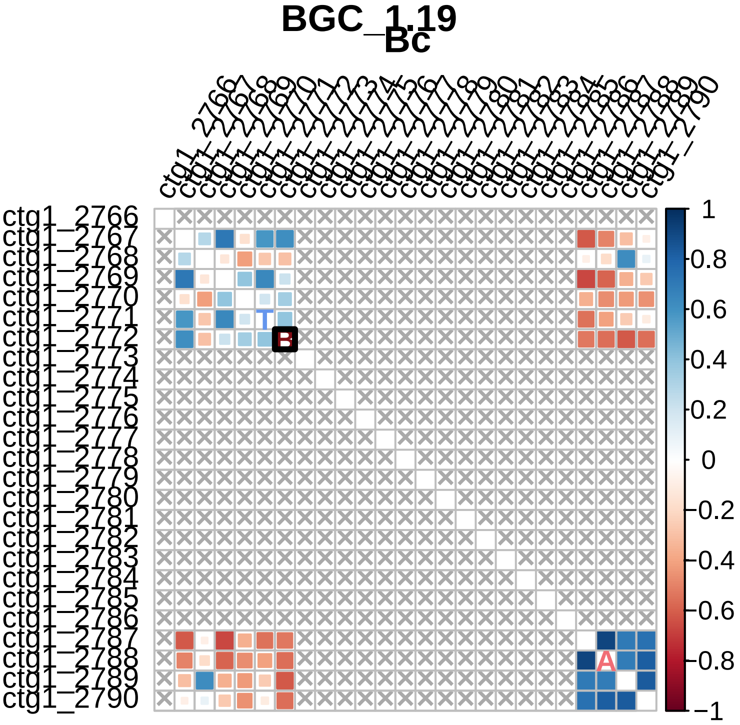
BGC_1.19
_Bc
ctg1_2766
ctg1_2767
ctg1_2768
ctg1_2769
ctg1_2770
ctg1_2771
ctg1_2772
ctg1_2773
ctg1_2774
ctg1_2775
ctg1_2776
ctg1_2777
ctg1_2778
ctg1_2779
ctg1_2780
ctg1_2781
ctg1_2782
ctg1_2783
ctg1_2784
ctg1_2785
ctg1_2786
ctg1_2787
ctg1_2788
ctg1_2789
ctg1_2790
T
B
A
1
0.8
0.6
0.4
0.2
0
−0.2
−0.4
−0.6
−0.8
−1

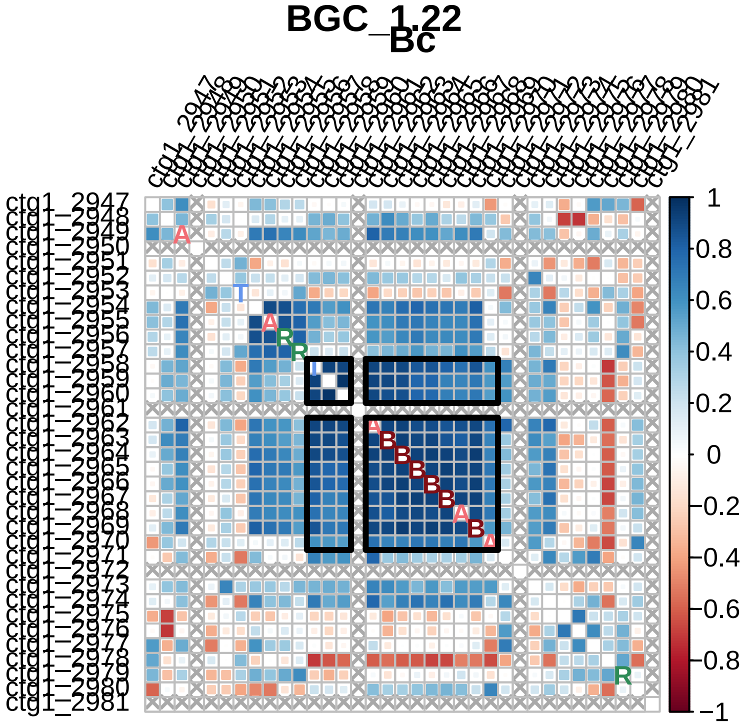

BGC_1.22
_Bc
ctg1_2947
ctg1_2948
ctg1_2949
ctg1_2950
ctg1_2951
ctg1_2952
ctg1_2953
ctg1_2954
ctg1_2955
ctg1_2956
ctg1_2957
ctg1_2958
ctg1_2959
ctg1_2960
ctg1_2961
ctg1_2962
ctg1_2963
ctg1_2964
ctg1_2965
ctg1_2966
ctg1_2967
ctg1_2968
ctg1_2969
ctg1_2970
ctg1_2971
ctg1_2972
ctg1_2973
ctg1_2974
ctg1_2975
ctg1_2976
ctg1_2977
ctg1_2978
ctg1_2979
ctg1_2980
ctg1_2981
1
0.8
0.6
0.4
0.2
0
−0.2
−0.4
−0.6
−0.8
−1

BGC_1.24a

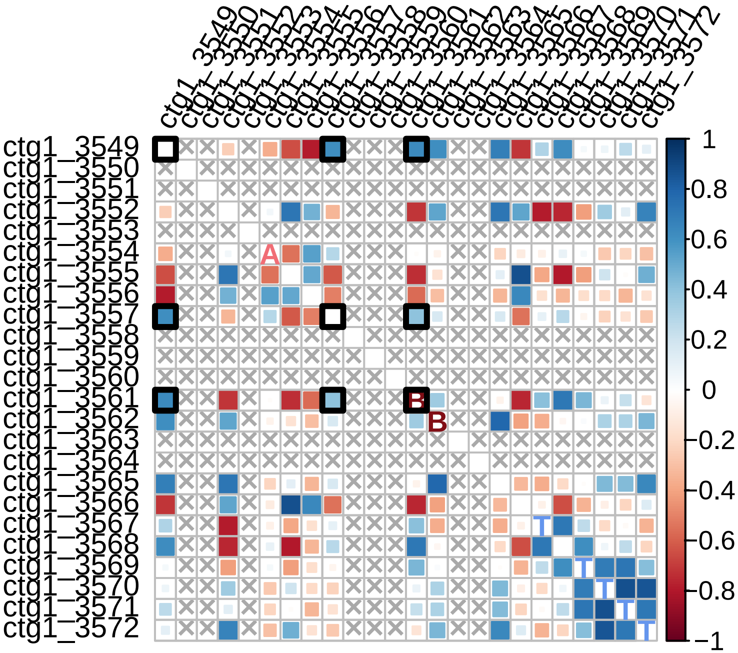

BGC_1.24b
Bc

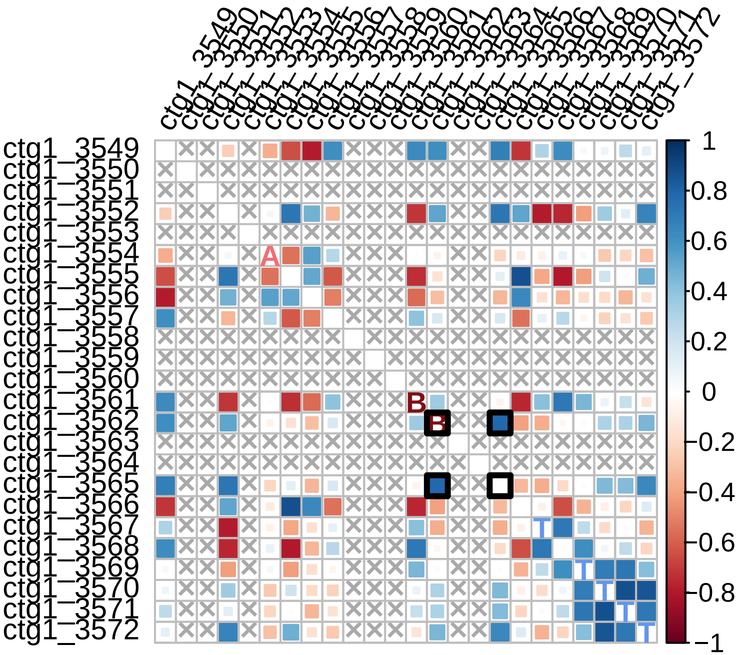

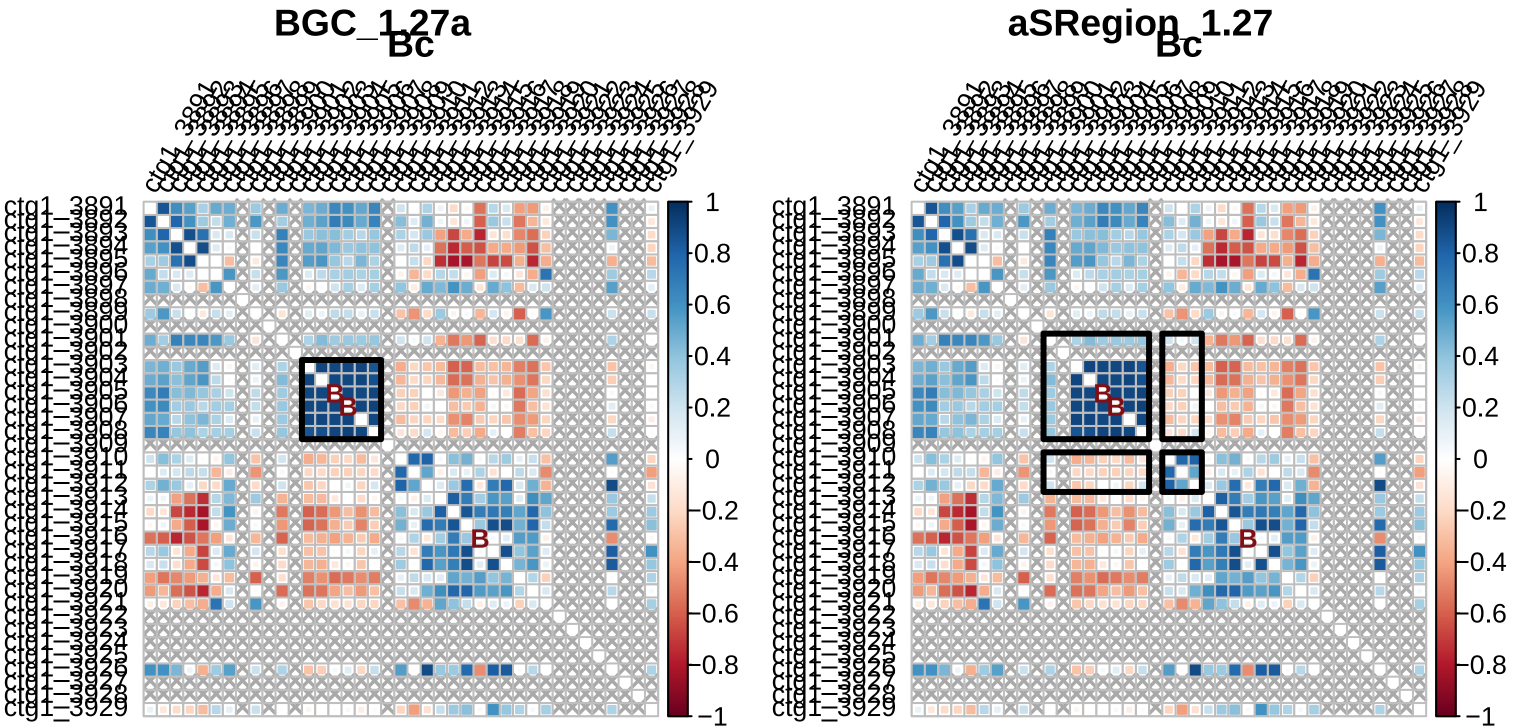

BGC_1.27a
Bc
aSRegion_1.27
Bc
ctg1_3891
ctg1_3929
1
0.8
0.6
0.4
0.2
0
−0.2
−0.4
−0.6
−0.8
−1
B

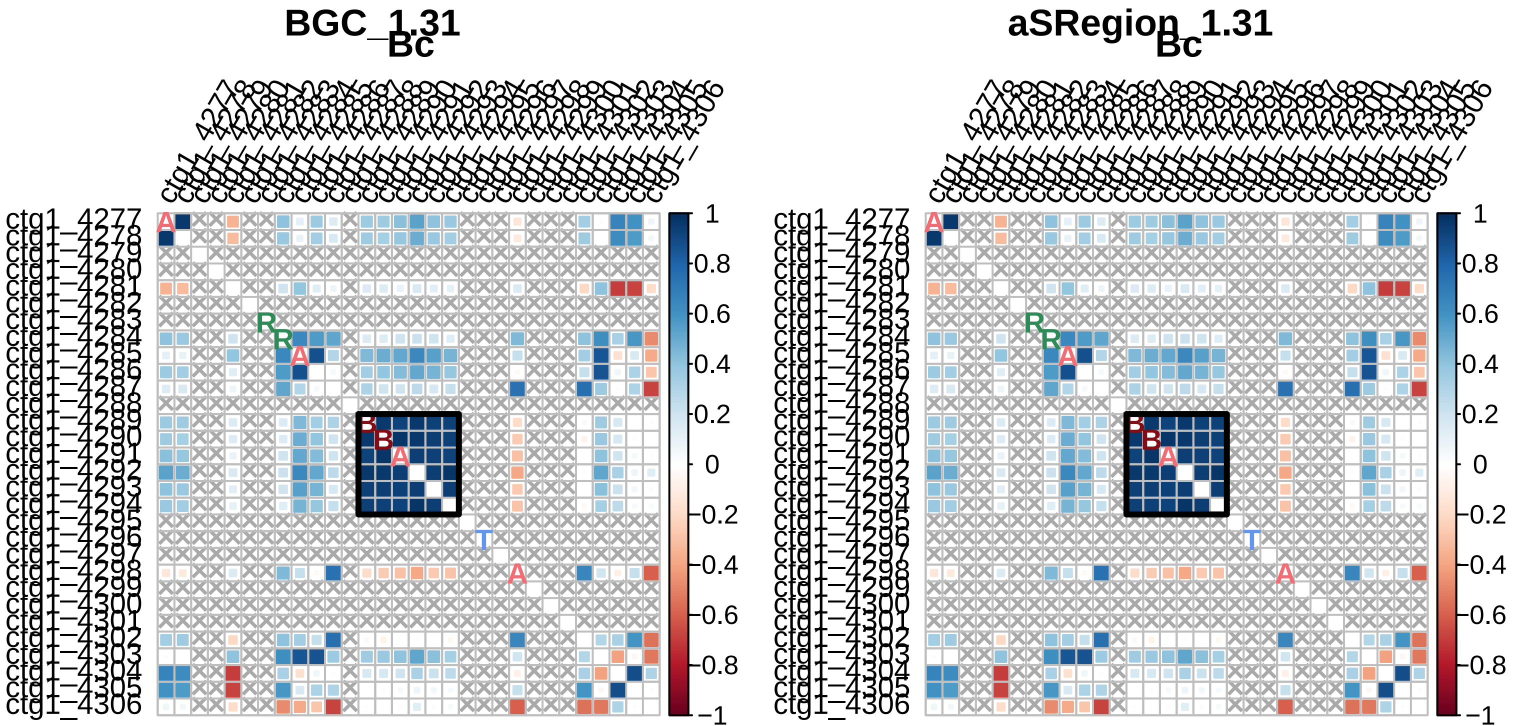
BGC_1.31 Bc
aSRegion_1.31 Bc
ctg1_4277
ctg1_4278
ctg1_4279
ctg1_4280
ctg1_4281
ctg1_4282
ctg1_4283
ctg1_4284
ctg1_4285
ctg1_4286
ctg1_4287
ctg1_4288
ctg1_4289
ctg1_4290
ctg1_4291
ctg1_4292
ctg1_4293
ctg1_4294
ctg1_4295
ctg1_4296
ctg1_4297
ctg1_4298
ctg1_4299
ctg1_4300
ctg1_4301
ctg1_4302
ctg1_4303
ctg1_4304
ctg1_4305
ctg1_4306
1
0.8
0.6
0.4
0.2
0
−0.2
−0.4
−0.6
−0.8
−1

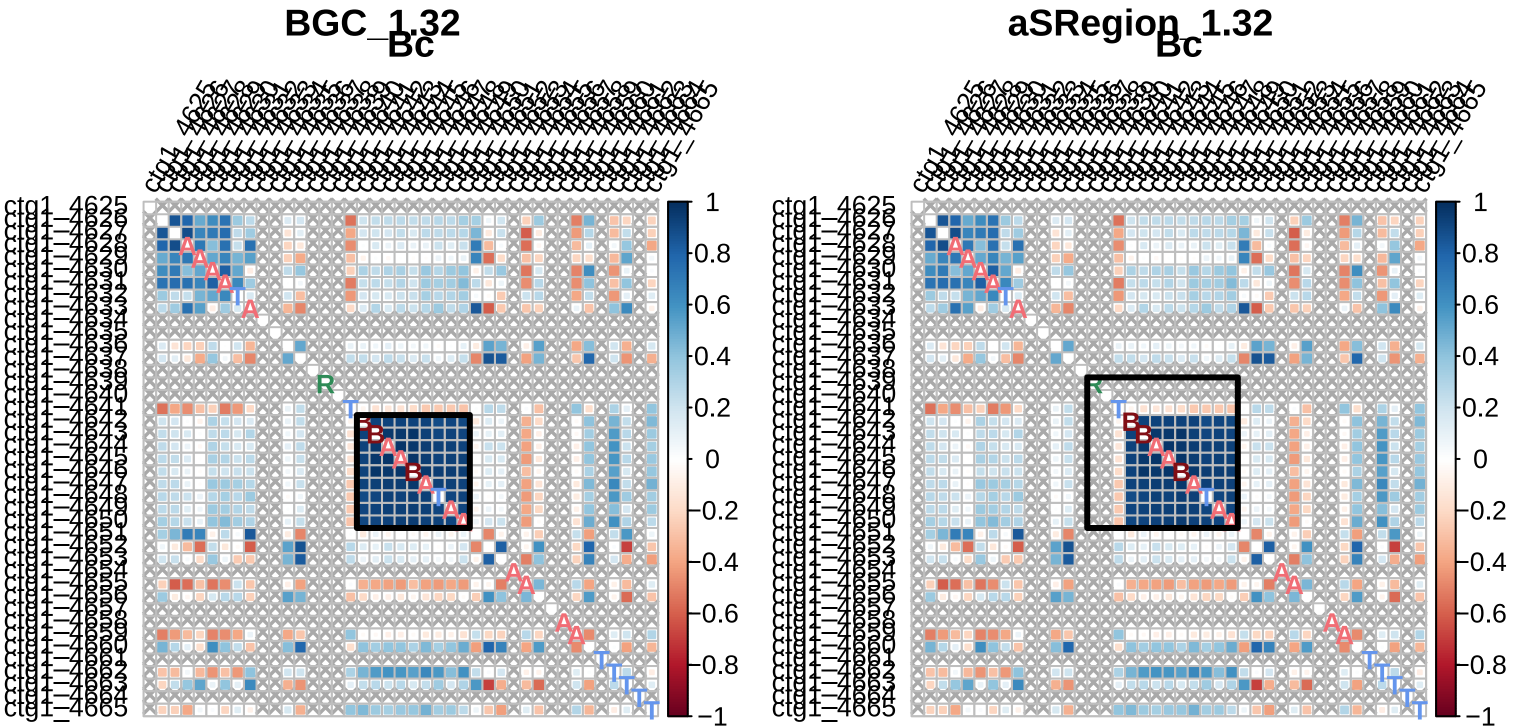
BGC_1.32
Bc
aSRegion_1.32
Bc
ctg1_4625
ctg1_4665
1
0.8
0.6
0.4
0.2
0
-0.2
-0.4
-0.6
-0.8
-1
R
T
B
A

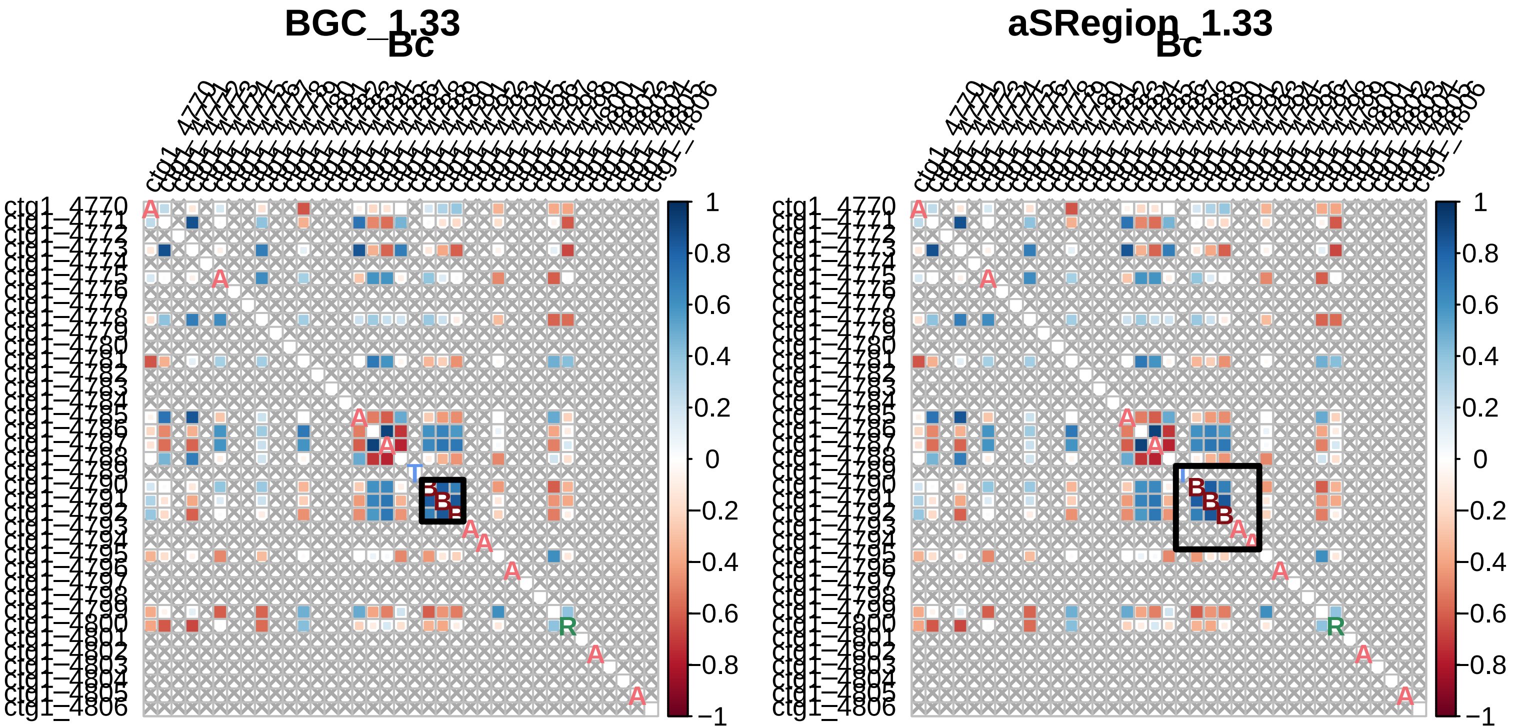
BGC_1.33
Bc
aSRegion_1.33
Bc
ctg1_4770
ctg1_4771
ctg1_4772
ctg1_4773
ctg1_4774
ctg1_4775
ctg1_4776
ctg1_4777
ctg1_4778
ctg1_4779
ctg1_4780
ctg1_4781
ctg1_4782
ctg1_4783
ctg1_4784
ctg1_4785
ctg1_4786
ctg1_4787
ctg1_4788
ctg1_4789
ctg1_4790
ctg1_4791
ctg1_4792
ctg1_4793
ctg1_4794
ctg1_4795
ctg1_4796
ctg1_4797
ctg1_4798
ctg1_4799
ctg1_4800
ctg1_4801
ctg1_4802
ctg1_4803
ctg1_4804
ctg1_4805
ctg1_4806
1
0.8
0.6
0.4
0.2
0
-0.2
-0.4
-0.6
-0.8
-1

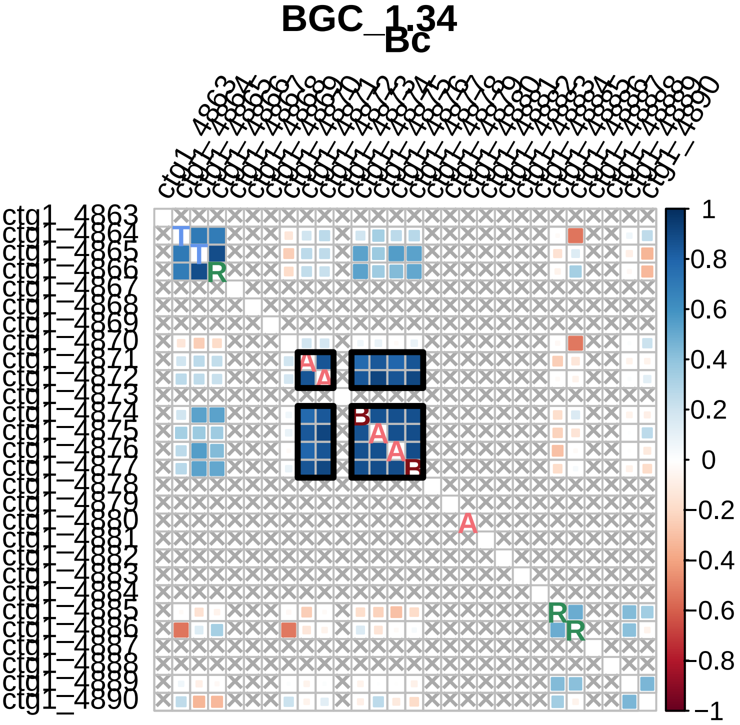
BGC_1.34_Bc
ctg1_4863
ctg1_4864
ctg1_4865
ctg1_4866
ctg1_4867
ctg1_4868
ctg1_4869
ctg1_4870
ctg1_4871
ctg1_4872
ctg1_4873
ctg1_4874
ctg1_4875
ctg1_4876
ctg1_4877
ctg1_4878
ctg1_4879
ctg1_4880
ctg1_4881
ctg1_4882
ctg1_4883
ctg1_4884
ctg1_4885
ctg1_4886
ctg1_4887
ctg1_4888
ctg1_4889
ctg1_4890
T
R
A
B
1
0.8
0.6
0.4
0.2
0
−0.2
−0.4
−0.6
−0.8
−1

BGC_1_38a

Bc

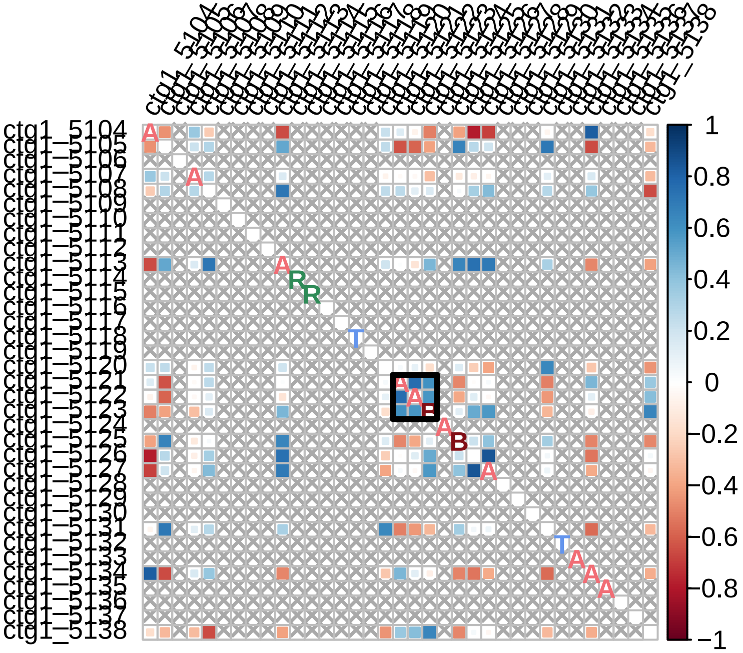

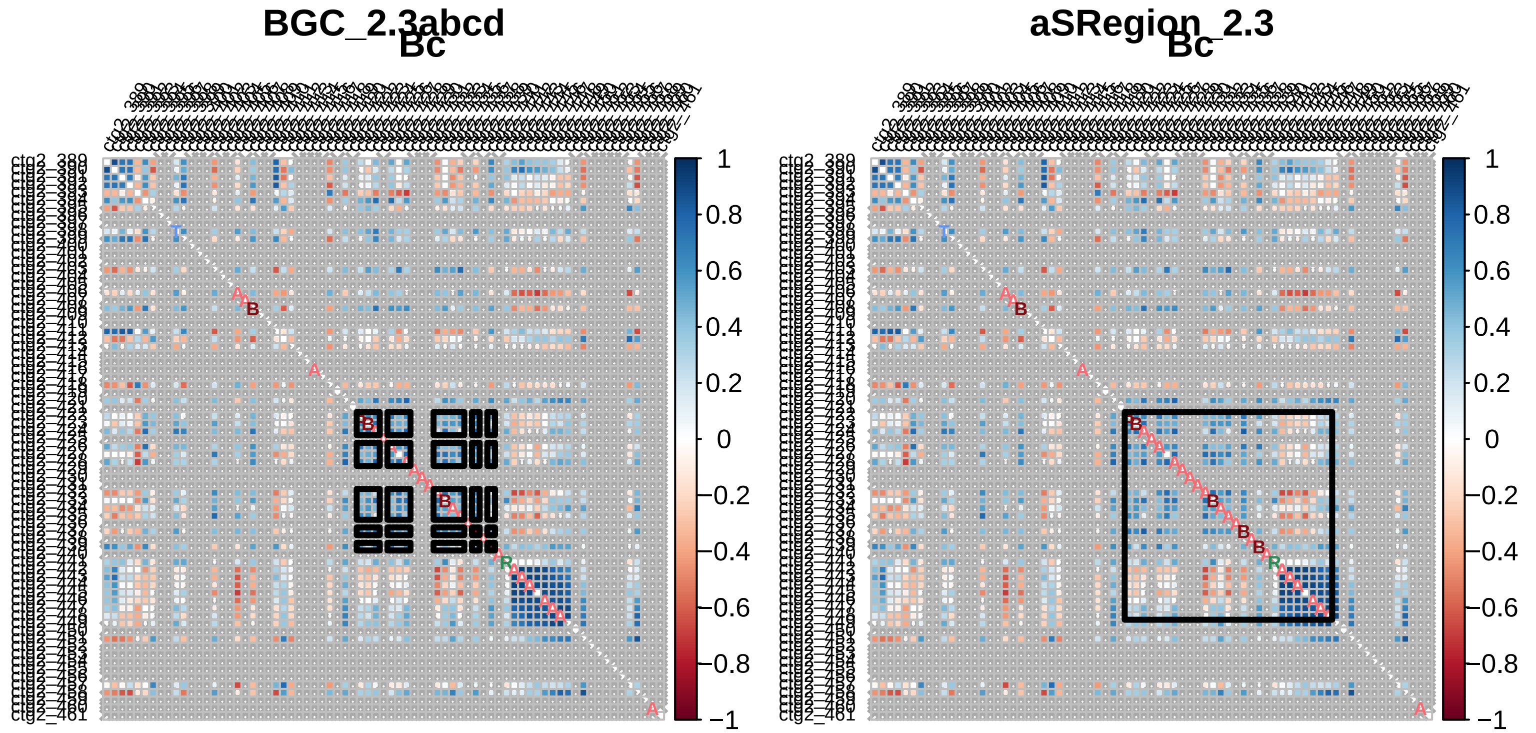
BGC_2.3abcd
Bc
aSRegion_2.3
Bc
1
0.8
0.6
0.4
0.2
0
−0.2
−0.4
−0.6
−0.8
−1

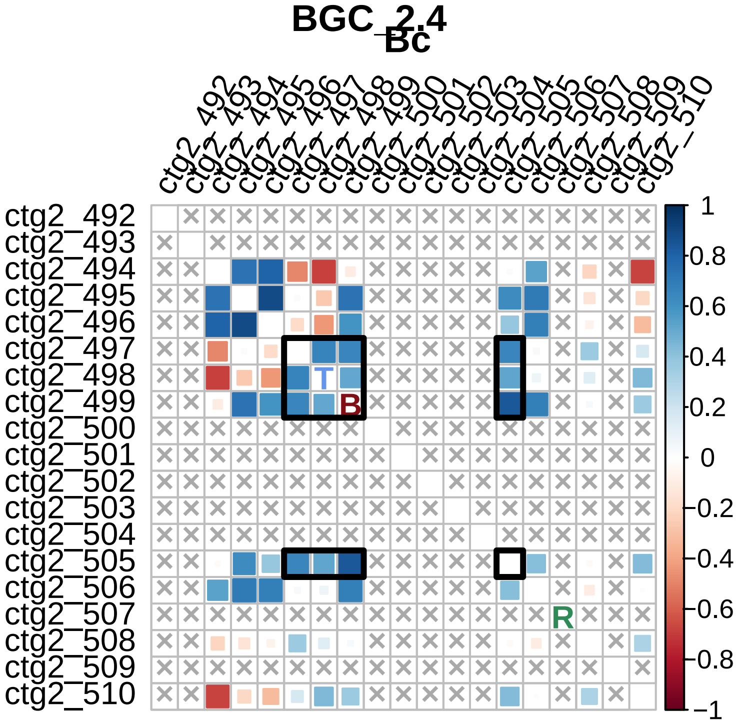
BGC_2.4
Bc
ctg2_492
ctg2_493
ctg2_494
ctg2_495
ctg2_496
ctg2_497
ctg2_498
ctg2_499
ctg2_500
ctg2_501
ctg2_502
ctg2_503
ctg2_504
ctg2_505
ctg2_506
ctg2_507
ctg2_508
ctg2_509
ctg2_510
T
B
R
1
0.8
0.6
0.4
0.2
0
−0.2
−0.4
−0.6
−0.8
−1

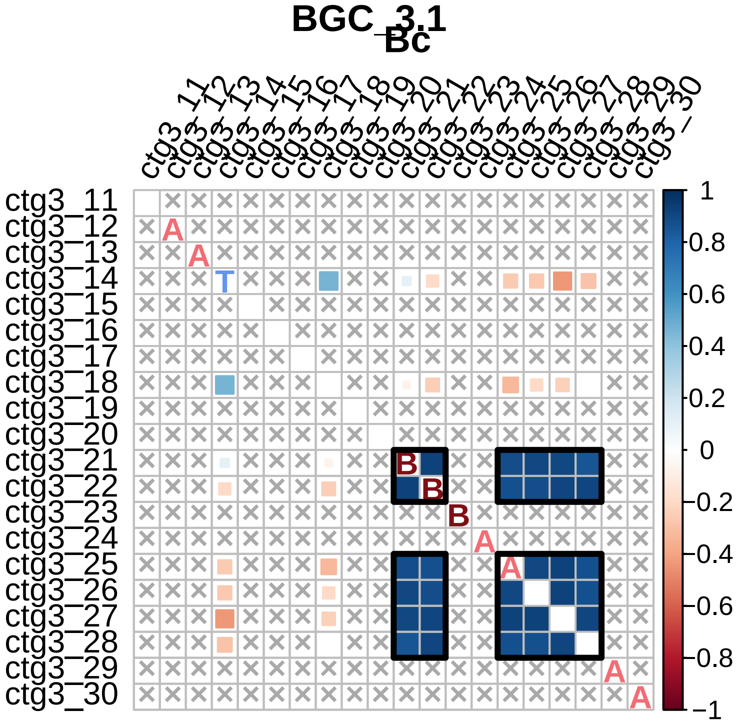
BGC_3.1_Bc
ctg3_11
ctg3_12
ctg3_13
ctg3_14
ctg3_15
ctg3_16
ctg3_17
ctg3_18
ctg3_19
ctg3_20
ctg3_21
ctg3_22
ctg3_23
ctg3_24
ctg3_25
ctg3_26
ctg3_27
ctg3_28
ctg3_29
ctg3_30
A
T
B
1
0.8
0.6
0.4
0.2
0
−0.2
−0.4
−0.6
−0.8
−1

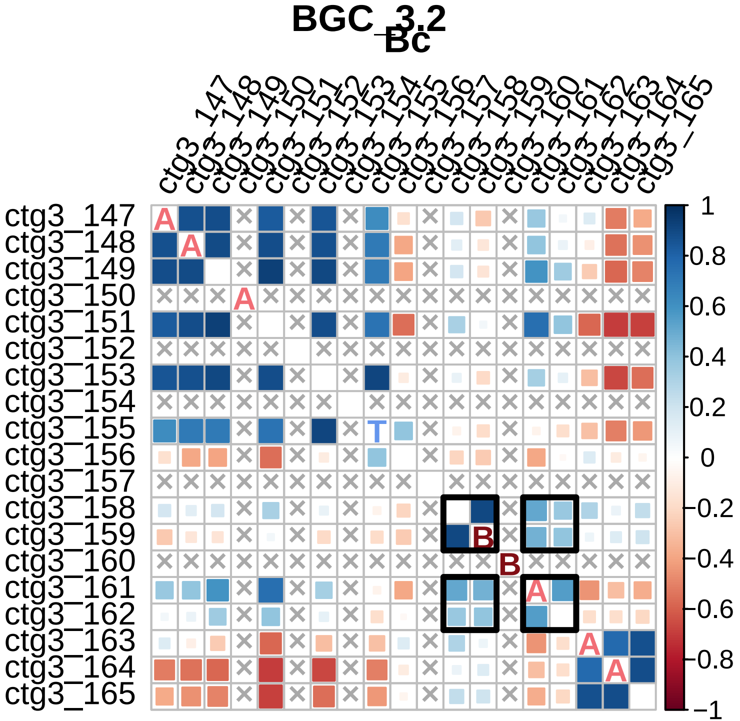
BGC_3.2
_Bc
ctg3_147
ctg3_148
ctg3_149
ctg3_150
ctg3_151
ctg3_152
ctg3_153
ctg3_154
ctg3_155
ctg3_156
ctg3_157
ctg3_158
ctg3_159
ctg3_160
ctg3_161
ctg3_162
ctg3_163
ctg3_164
ctg3_165
A
T
B
1
0.8
0.6
0.4
0.2
0
−0.2
−0.4
−0.6
−0.8
−1

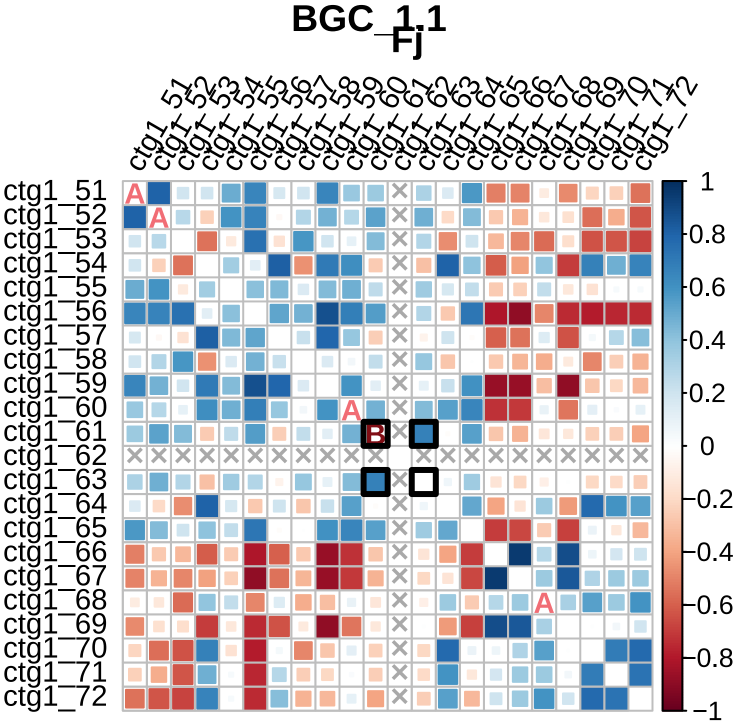

BGC_1.1
Fj
ctg1_51
ctg1_52
ctg1_53
ctg1_54
ctg1_55
ctg1_56
ctg1_57
ctg1_58
ctg1_59
ctg1_60
ctg1_61
ctg1_62
ctg1_63
ctg1_64
ctg1_65
ctg1_66
ctg1_67
ctg1_68
ctg1_69
ctg1_70
ctg1_71
ctg1_72
A
B
1
0.8
0.6
0.4
0.2
0
−0.2
−0.4
−0.6
−0.8
−1

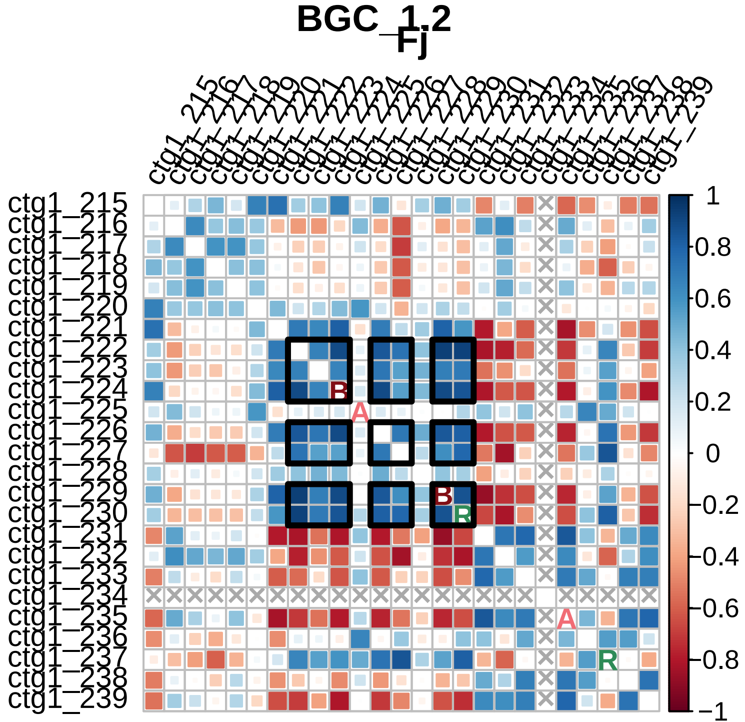
BGC_1.2_Fj
ctg1_215
ctg1_216
ctg1_217
ctg1_218
ctg1_219
ctg1_220
ctg1_221
ctg1_222
ctg1_223
ctg1_224
ctg1_225
ctg1_226
ctg1_227
ctg1_228
ctg1_229
ctg1_230
ctg1_231
ctg1_232
ctg1_233
ctg1_234
ctg1_235
ctg1_236
ctg1_237
ctg1_238
ctg1_239
A
B
R
1
0.8
0.6
0.4
0.2
0
−0.2
−0.4
−0.6
−0.8
−1

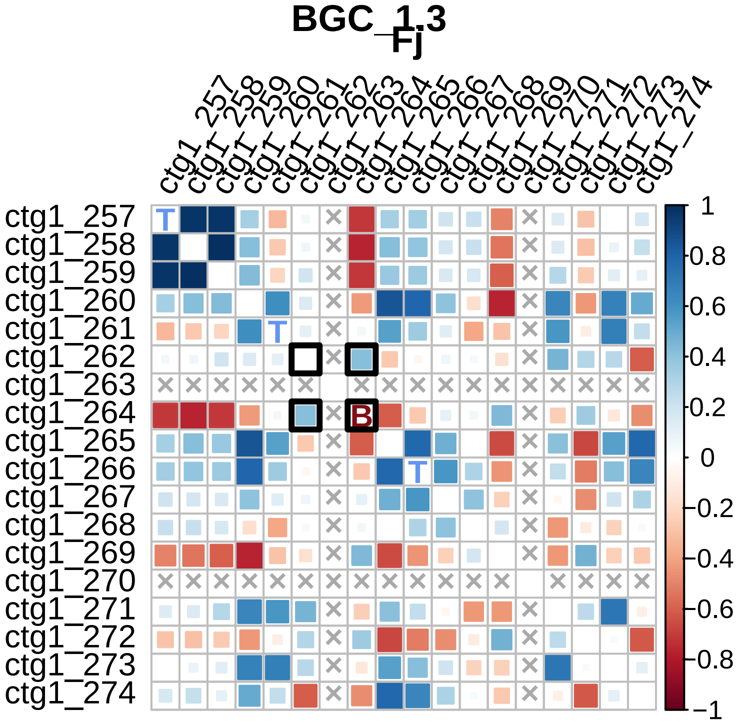
BGC_1.3
_Fj
ctg1_257
ctg1_258
ctg1_259
ctg1_260
ctg1_261
ctg1_262
ctg1_263
ctg1_264
ctg1_265
ctg1_266
ctg1_267
ctg1_268
ctg1_269
ctg1_270
ctg1_271
ctg1_272
ctg1_273
ctg1_274
T
B
1
0.8
0.6
0.4
0.2
0
−0.2
−0.4
−0.6
−0.8
−1

BGC_1.4a

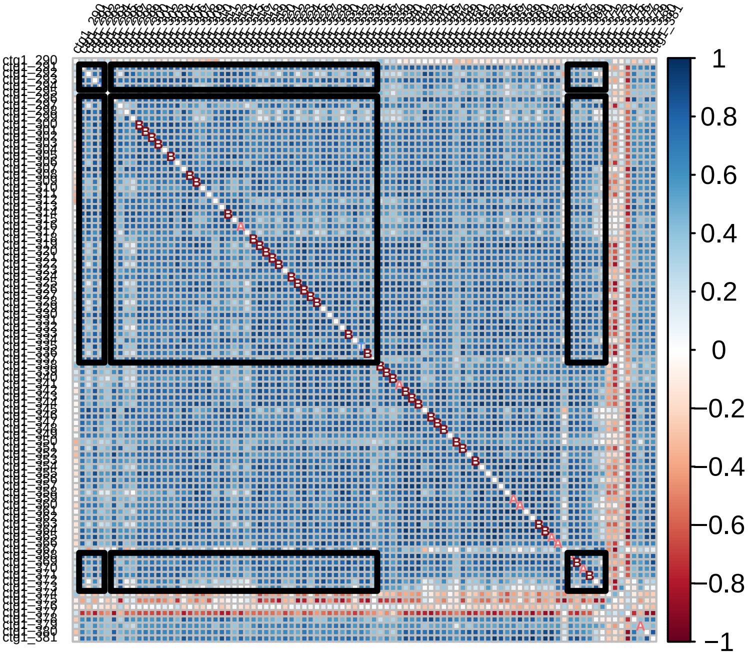

BGC_1.4b
Fj

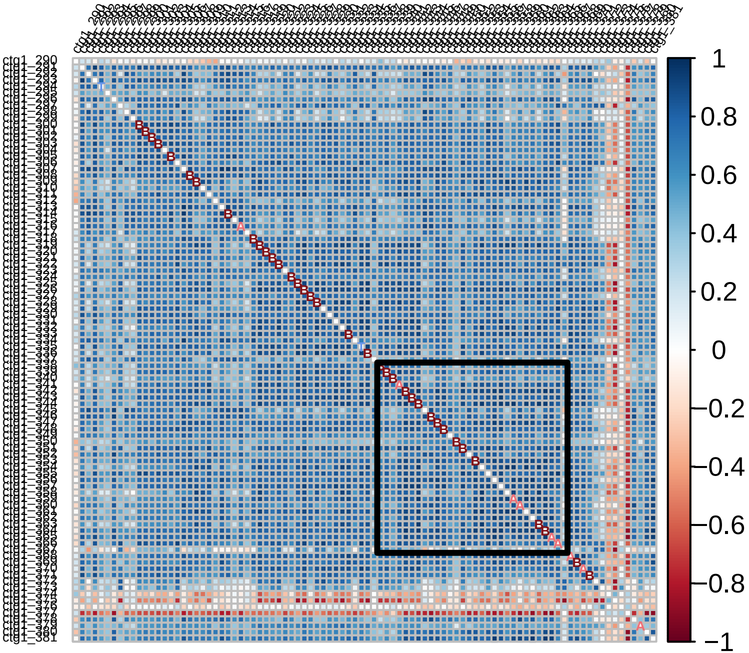

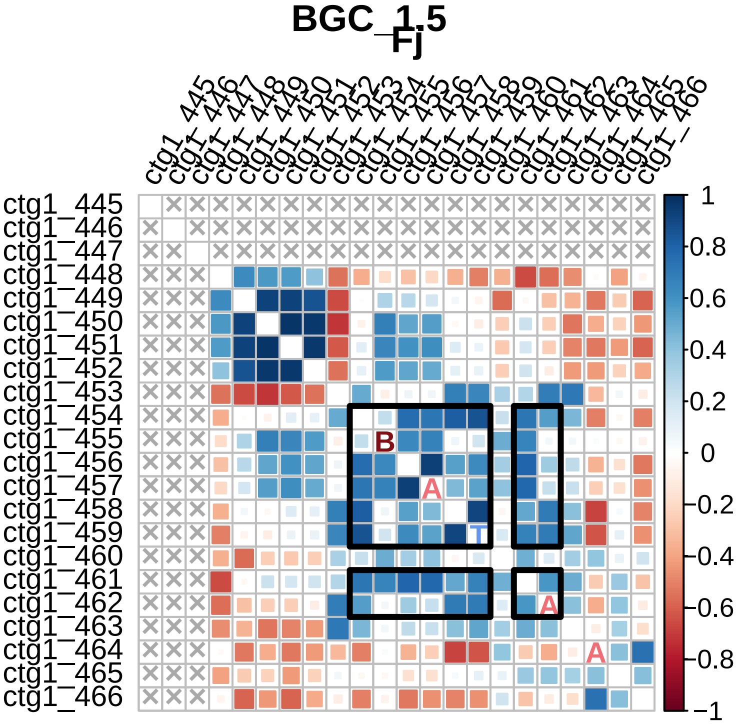

BGC_1.5
Fj
ctg1_445
ctg1_446
ctg1_447
ctg1_448
ctg1_449
ctg1_450
ctg1_451
ctg1_452
ctg1_453
ctg1_454
ctg1_455
ctg1_456
ctg1_457
ctg1_458
ctg1_459
ctg1_460
ctg1_461
ctg1_462
ctg1_463
ctg1_464
ctg1_465
ctg1_466
B
A
T
A
A
1
0.8
0.6
0.4
0.2
0
−0.2
−0.4
−0.6
−0.8
−1

BGC_1.6a

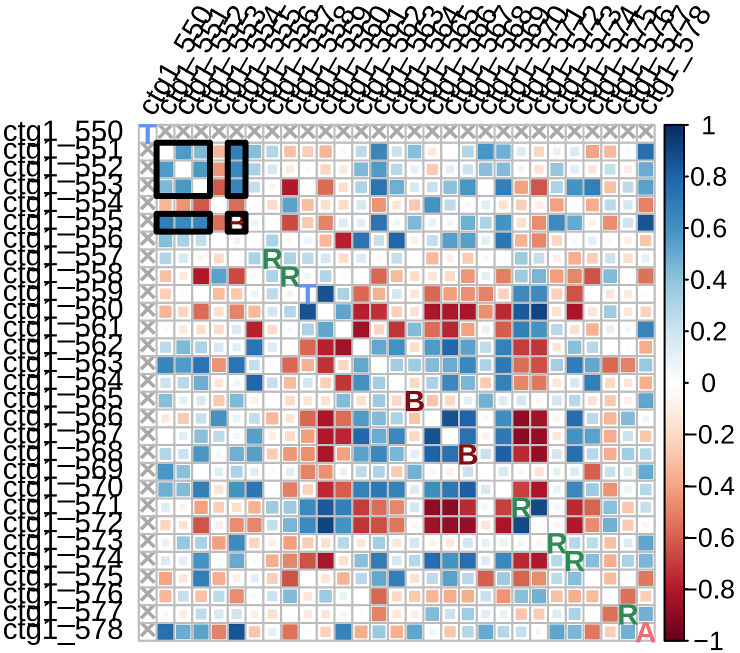

BGC_1.6b

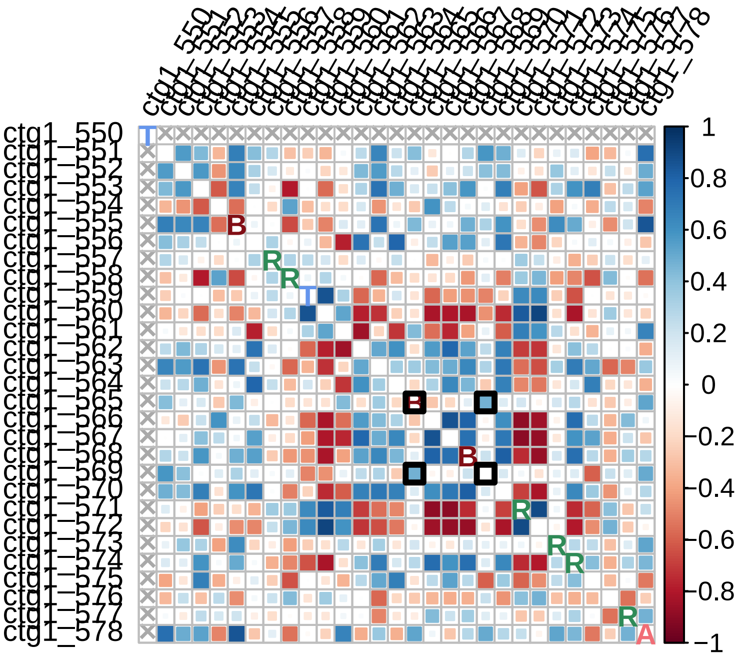

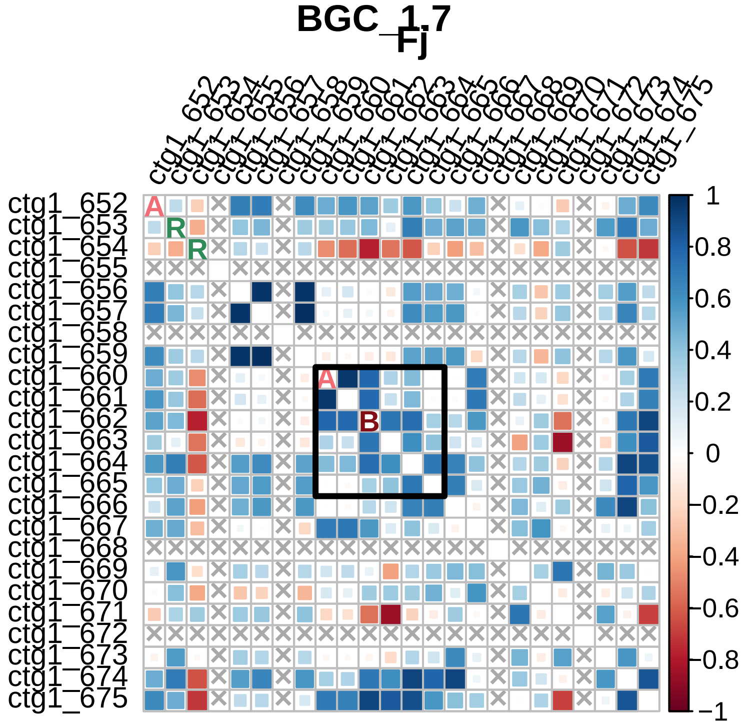

BGC_1.7_Fj
ctg1_652
ctg1_653
ctg1_654
ctg1_655
ctg1_656
ctg1_657
ctg1_658
ctg1_659
ctg1_660
ctg1_661
ctg1_662
ctg1_663
ctg1_664
ctg1_665
ctg1_666
ctg1_667
ctg1_668
ctg1_669
ctg1_670
ctg1_671
ctg1_672
ctg1_673
ctg1_674
ctg1_675
A
R
R
A
B
1
0.8
0.6
0.4
0.2
0
−0.2
−0.4
−0.6
−0.8
−1

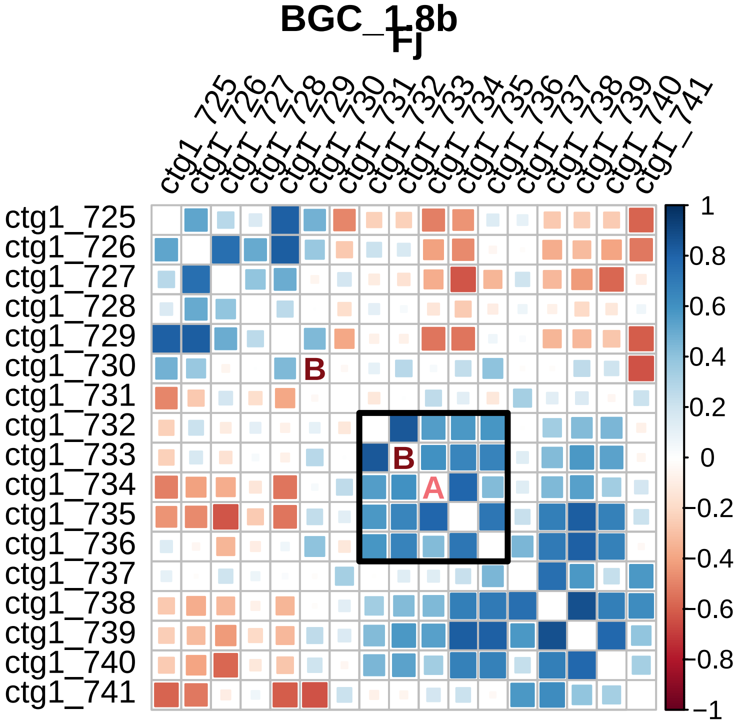
BGC_1.8b
Fj
ctg1_725
ctg1_726
ctg1_727
ctg1_728
ctg1_729
ctg1_730
ctg1_731
ctg1_732
ctg1_733
ctg1_734
ctg1_735
ctg1_736
ctg1_737
ctg1_738
ctg1_739
ctg1_740
ctg1_741
B
B
A
1
0.8
0.6
0.4
0.2
0
−0.2
−0.4
−0.6
−0.8
−1

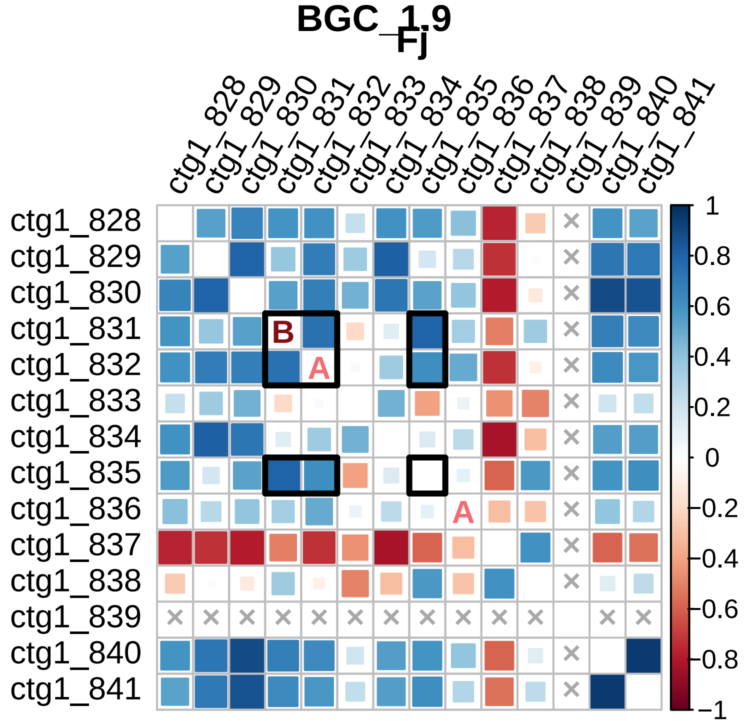

BGC_1.9
_Fj
ctg1_828
ctg1_829
ctg1_830
ctg1_831
ctg1_832
ctg1_833
ctg1_834
ctg1_835
ctg1_836
ctg1_837
ctg1_838
ctg1_839
ctg1_840
ctg1_841
B
A
A
1
0.8
0.6
0.4
0.2
0
−0.2
−0.4
−0.6
−0.8
−1

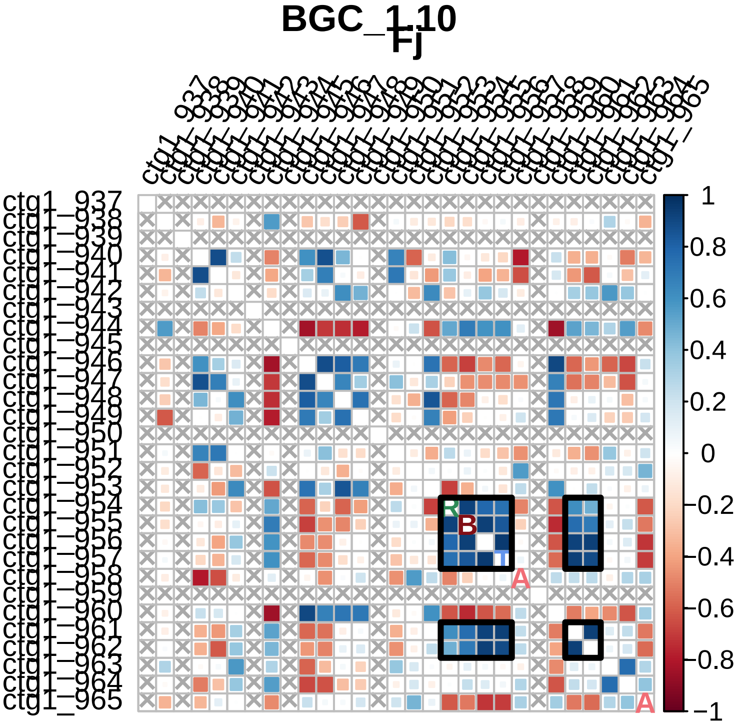

BGC_1.10
Fj
ctg1_937
ctg1_965
R
B
T
A
A
1
0.8
0.6
0.4
0.2
0
−0.2
−0.4
−0.6
−0.8
−1

BGC_1.11a

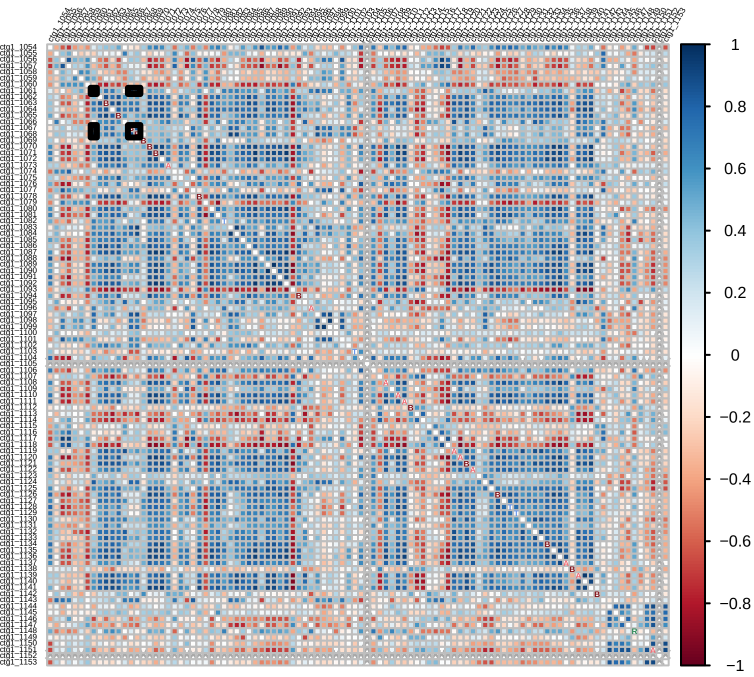

aSRegion_1.11
Fj

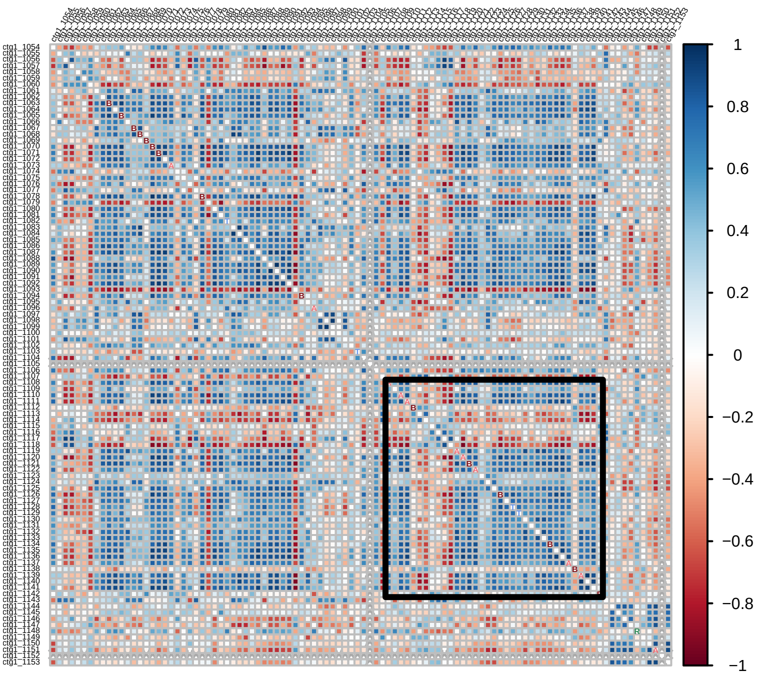

BGC_1.11b

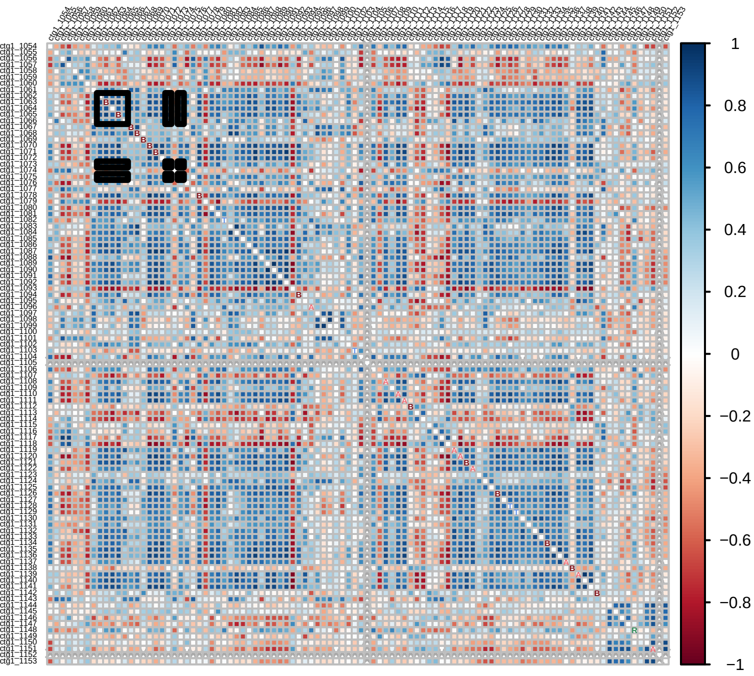

BGC_1.11e

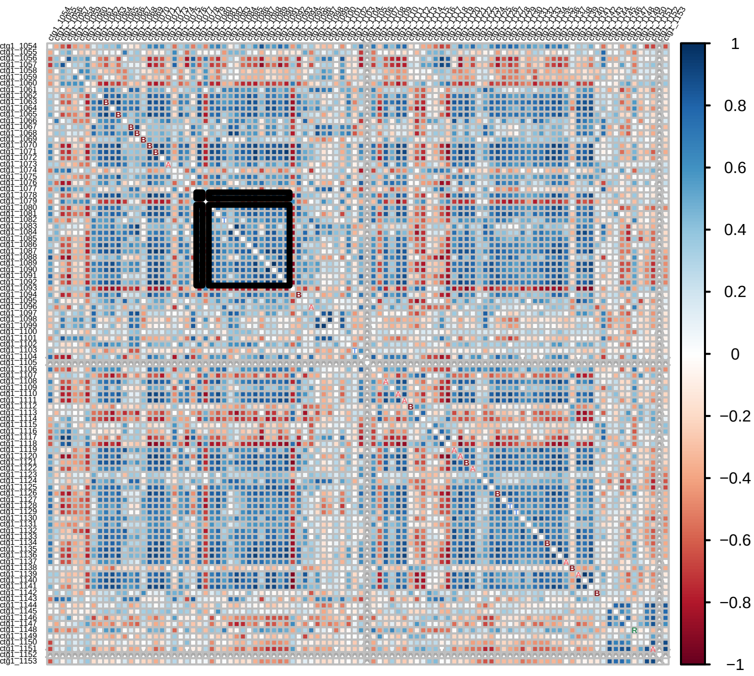

BGC_1.11g
Fj

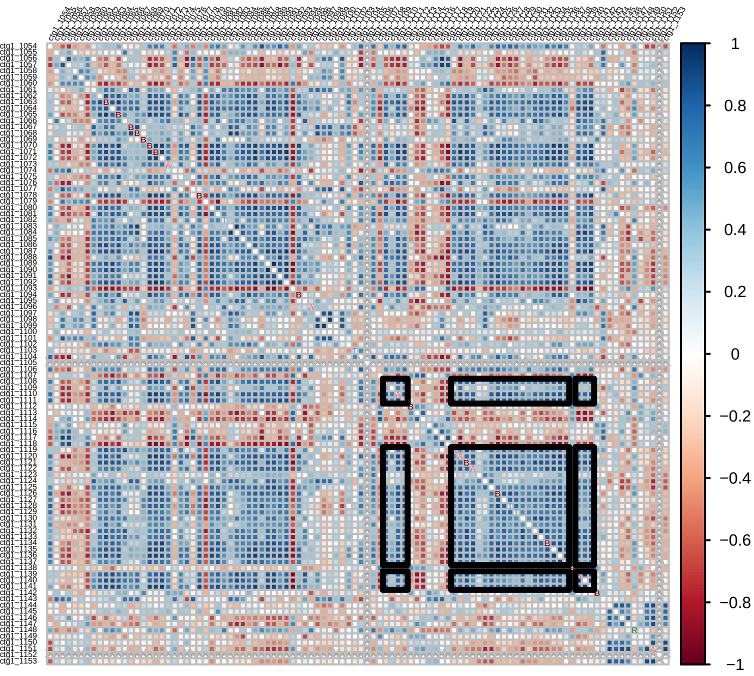

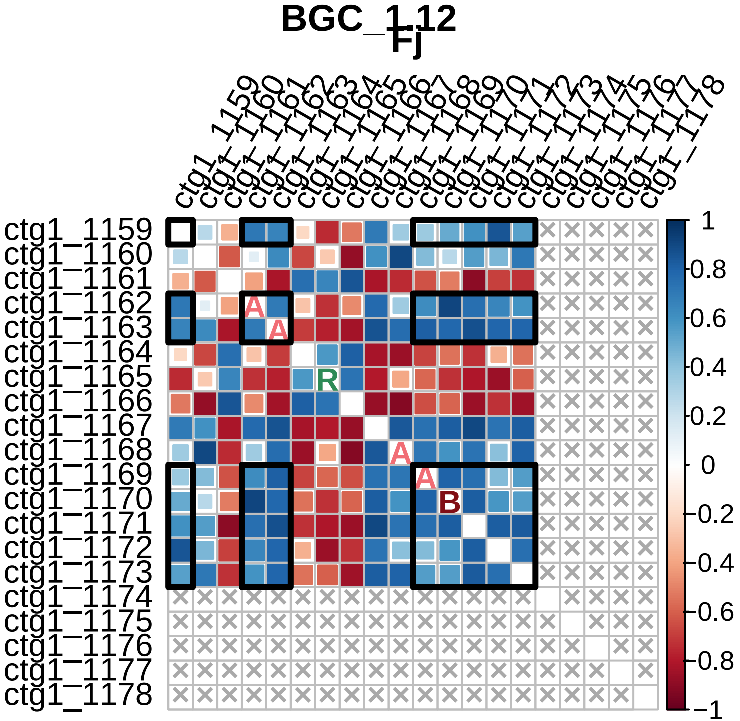

BGC_1.12
Fj
ctg1_1159
ctg1_1160
ctg1_1161
ctg1_1162
ctg1_1163
ctg1_1164
ctg1_1165
ctg1_1166
ctg1_1167
ctg1_1168
ctg1_1169
ctg1_1170
ctg1_1171
ctg1_1172
ctg1_1173
ctg1_1174
ctg1_1175
ctg1_1176
ctg1_1177
ctg1_1178
A
A
R
A
A
B
1
0.8
0.6
0.4
0.2
0
−0.2
−0.4
−0.6
−0.8
−1

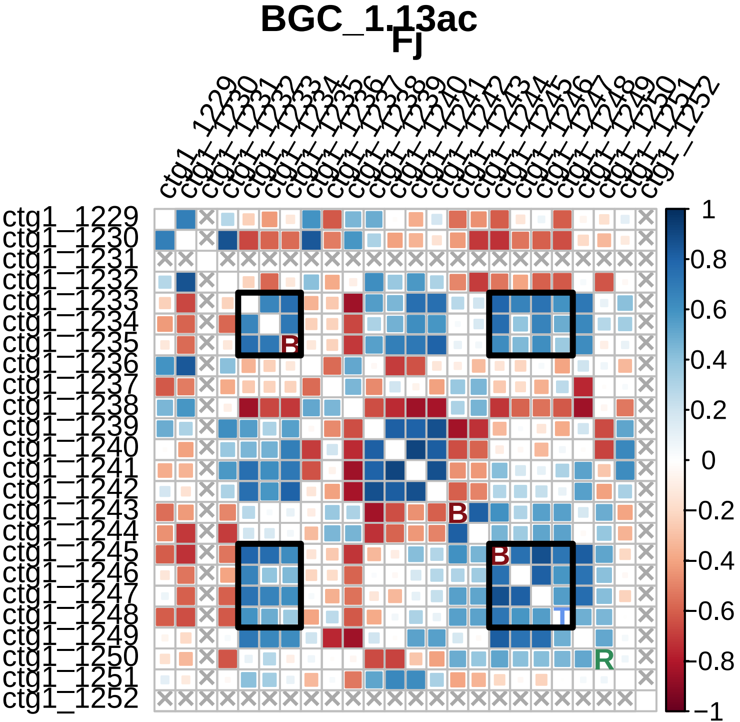

BGC_1.13ac
Fj
ctg1_1229
ctg1_1230
ctg1_1231
ctg1_1232
ctg1_1233
ctg1_1234
ctg1_1235
ctg1_1236
ctg1_1237
ctg1_1238
ctg1_1239
ctg1_1240
ctg1_1241
ctg1_1242
ctg1_1243
ctg1_1244
ctg1_1245
ctg1_1246
ctg1_1247
ctg1_1248
ctg1_1249
ctg1_1250
ctg1_1251
ctg1_1252
B
B
B
T
R
1
0.8
0.6
0.4
0.2
0
−0.2
−0.4
−0.6
−0.8
−1

BGC_1.14a

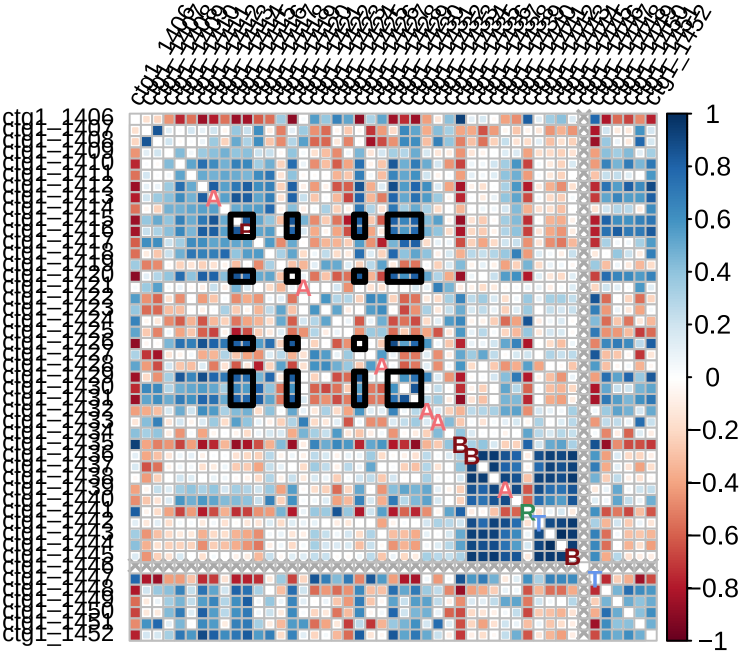

BGC_1.14c

FJ

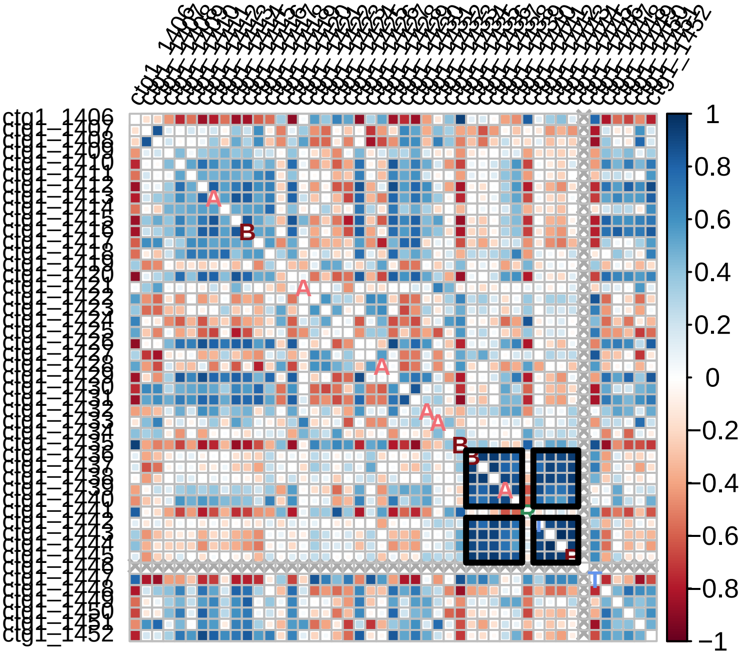

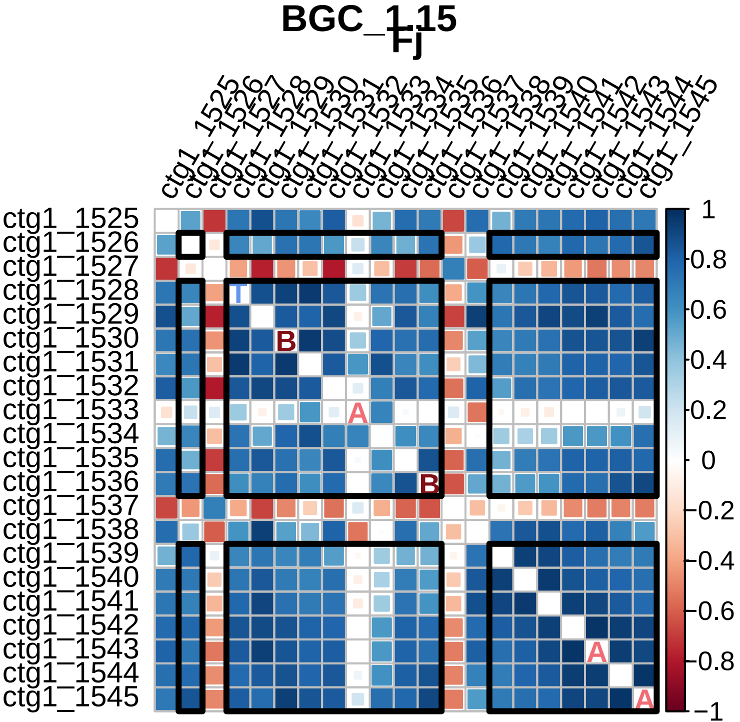
BGC_1.15
_Fj
ctg1_1525
ctg1_1526
ctg1_1527
ctg1_1528
ctg1_1529
ctg1_1530
ctg1_1531
ctg1_1532
ctg1_1533
ctg1_1534
ctg1_1535
ctg1_1536
ctg1_1537
ctg1_1538
ctg1_1539
ctg1_1540
ctg1_1541
ctg1_1542
ctg1_1543
ctg1_1544
ctg1_1545
T
B
A
B
A
A
1
0.8
0.6
0.4
0.2
0
−0.2
−0.4
−0.6
−0.8
−1

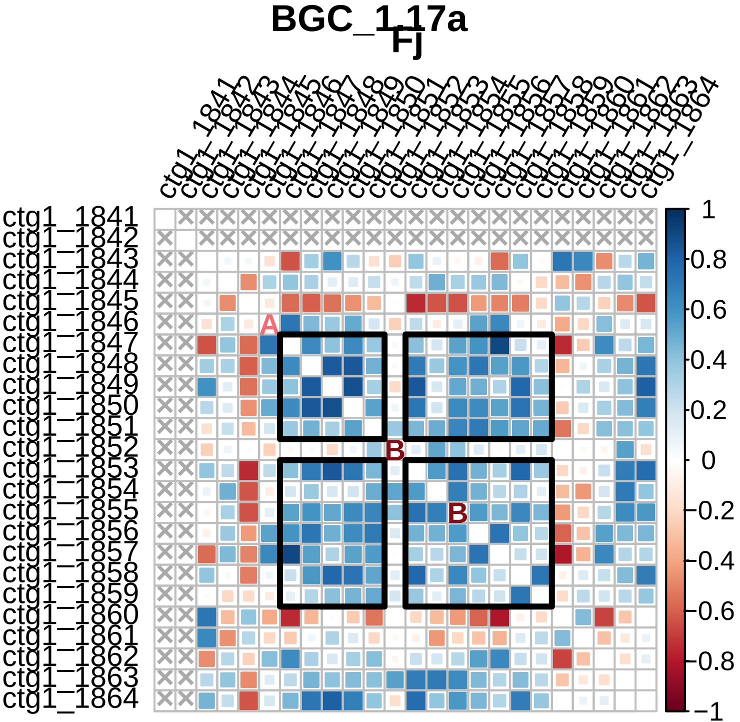

BGC_1_17a
Fj
ctg1_1841
ctg1_1842
ctg1_1843
ctg1_1844
ctg1_1845
ctg1_1846
ctg1_1847
ctg1_1848
ctg1_1849
ctg1_1850
ctg1_1851
ctg1_1852
ctg1_1853
ctg1_1854
ctg1_1855
ctg1_1856
ctg1_1857
ctg1_1858
ctg1_1859
ctg1_1860
ctg1_1861
ctg1_1862
ctg1_1863
ctg1_1864
A
B
B
1
0.8
0.6
0.4
0.2
0
−0.2
−0.4
−0.6
−0.8
−1

BGC_1_18a
FJ

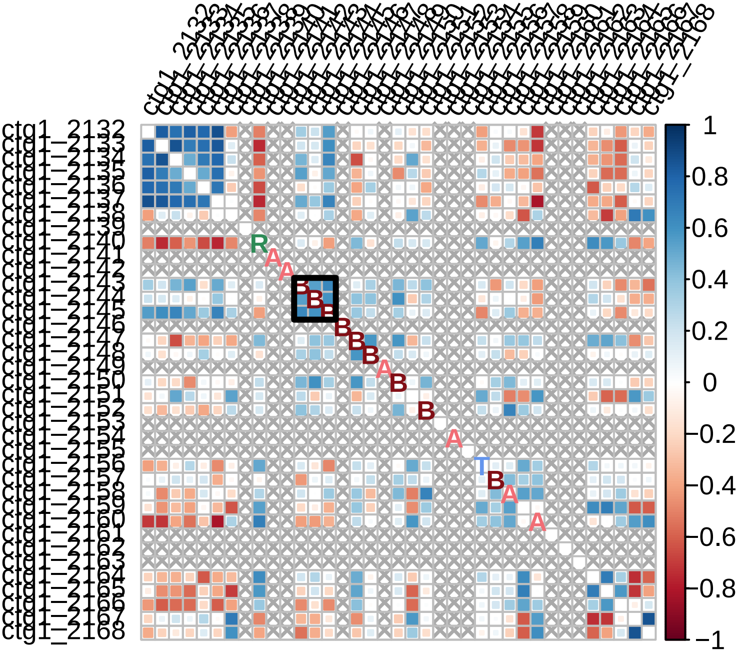
ctg1_2132
ctg1_2133
ctg1_2134
ctg1_2135
ctg1_2136
ctg1_2137
ctg1_2138
ctg1_2139
ctg1_2140
ctg1_2141
ctg1_2142
ctg1_2143
ctg1_2144
ctg1_2145
ctg1_2146
ctg1_2147
ctg1_2148
ctg1_2149
ctg1_2150
ctg1_2151
ctg1_2152
ctg1_2153
ctg1_2154
ctg1_2155
ctg1_2156
ctg1_2157
ctg1_2158
ctg1_2159
ctg1_2160
ctg1_2161
ctg1_2162
ctg1_2163
ctg1_2164
ctg1_2165
ctg1_2166
ctg1_2167
ctg1_2168
R
A
A
B
B
B
B
B
B
A
B
B
A
T
B
A
A
1
0.8
0.6
0.4
0.2
0
−0.2
−0.4
−0.6
−0.8
−1

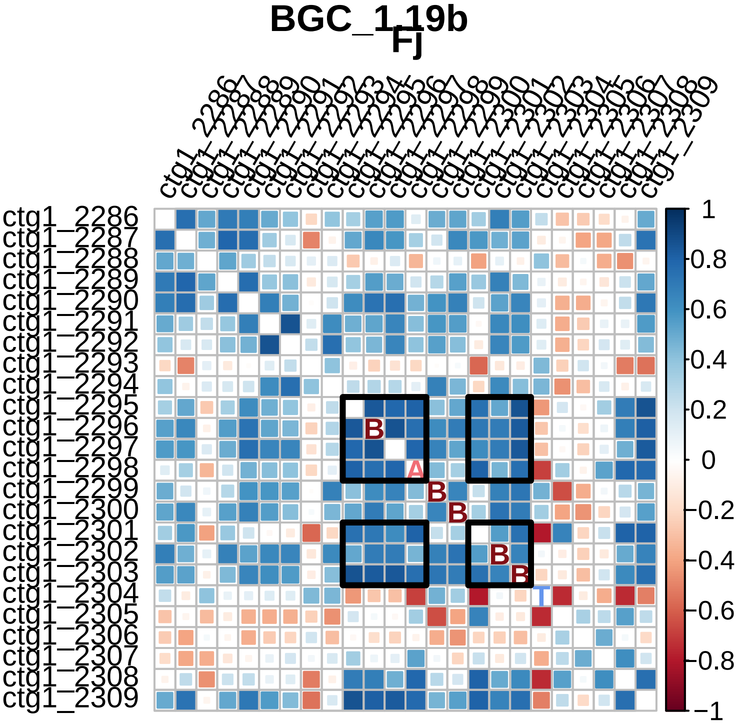

BGC_1_19b
Fj
ctg1_2286
ctg1_2287
ctg1_2288
ctg1_2289
ctg1_2290
ctg1_2291
ctg1_2292
ctg1_2293
ctg1_2294
ctg1_2295
ctg1_2296
ctg1_2297
ctg1_2298
ctg1_2299
ctg1_2300
ctg1_2301
ctg1_2302
ctg1_2303
ctg1_2304
ctg1_2305
ctg1_2306
ctg1_2307
ctg1_2308
ctg1_2309
B
A
B
B
B
B
T
1
0.8
0.6
0.4
0.2
0
−0.2
−0.4
−0.6
−0.8
−1

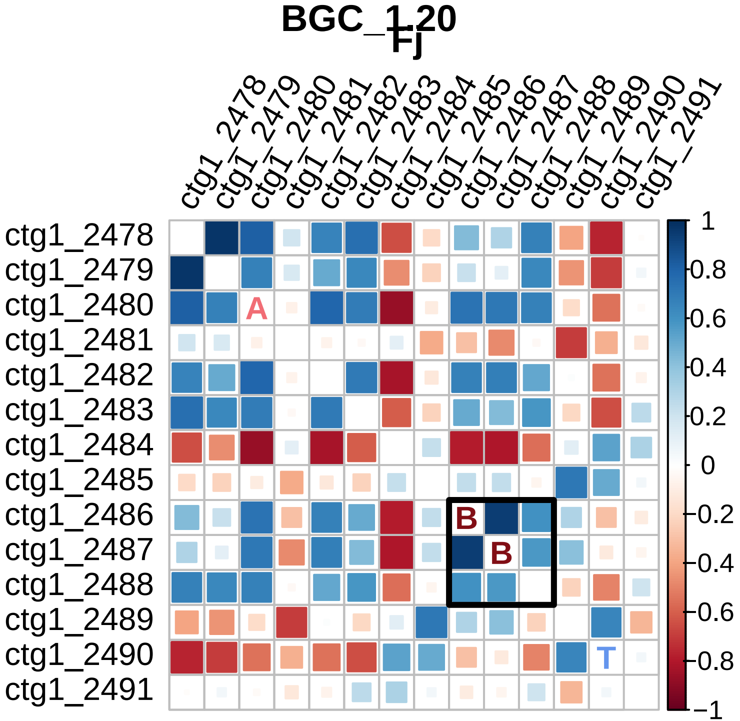
BGC_1.20
Fj
ctg1_2478
ctg1_2479
ctg1_2480
ctg1_2481
ctg1_2482
ctg1_2483
ctg1_2484
ctg1_2485
ctg1_2486
ctg1_2487
ctg1_2488
ctg1_2489
ctg1_2490
ctg1_2491
A
B
B
T
1
0.8
0.6
0.4
0.2
0
−0.2
−0.4
−0.6
−0.8
−1

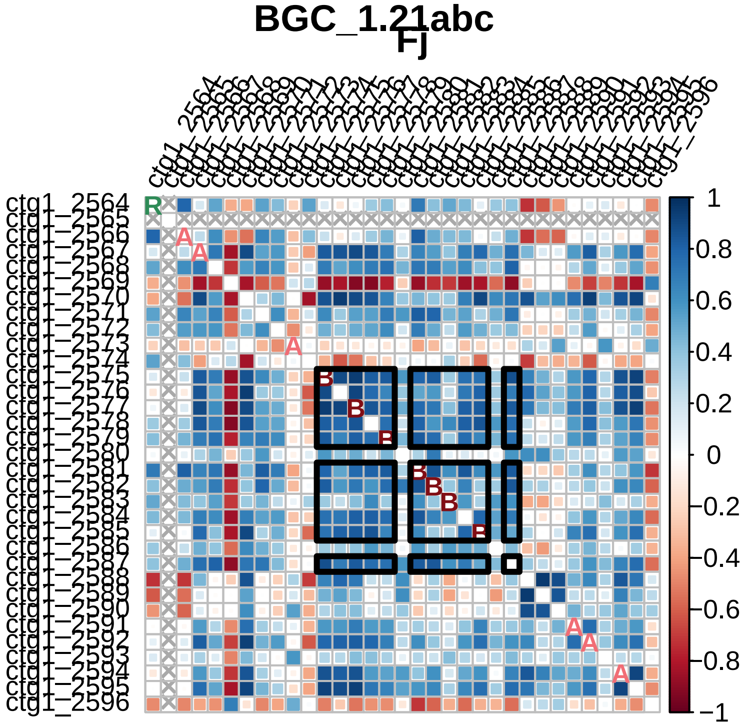

BGC_1.21abc
FJ
ctg1_2564
ctg1_2565
ctg1_2566
ctg1_2567
ctg1_2568
ctg1_2569
ctg1_2570
ctg1_2571
ctg1_2572
ctg1_2573
ctg1_2574
ctg1_2575
ctg1_2576
ctg1_2577
ctg1_2578
ctg1_2579
ctg1_2580
ctg1_2581
ctg1_2582
ctg1_2583
ctg1_2584
ctg1_2585
ctg1_2586
ctg1_2587
ctg1_2588
ctg1_2589
ctg1_2590
ctg1_2591
ctg1_2592
ctg1_2593
ctg1_2594
ctg1_2595
ctg1_2596
R
A
B
1
0.8
0.6
0.4
0.2
0
−0.2
−0.4
−0.6
−0.8
−1

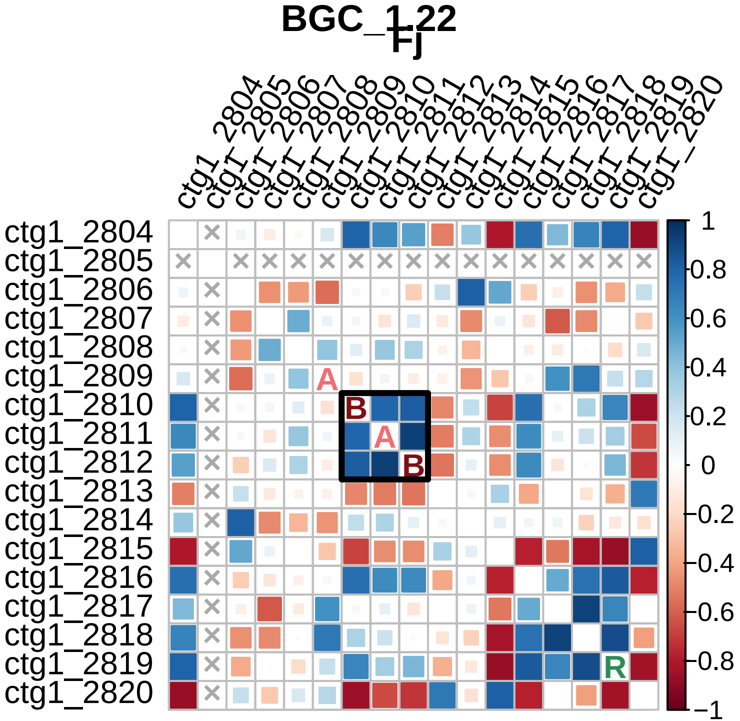
BGC_1.22
Fj
ctg1_2804
ctg1_2805
ctg1_2806
ctg1_2807
ctg1_2808
ctg1_2809
ctg1_2810
ctg1_2811
ctg1_2812
ctg1_2813
ctg1_2814
ctg1_2815
ctg1_2816
ctg1_2817
ctg1_2818
ctg1_2819
ctg1_2820
A
B
A
B
R
1
0.8
0.6
0.4
0.2
0
−0.2
−0.4
−0.6
−0.8
−1

BGC_1.23a

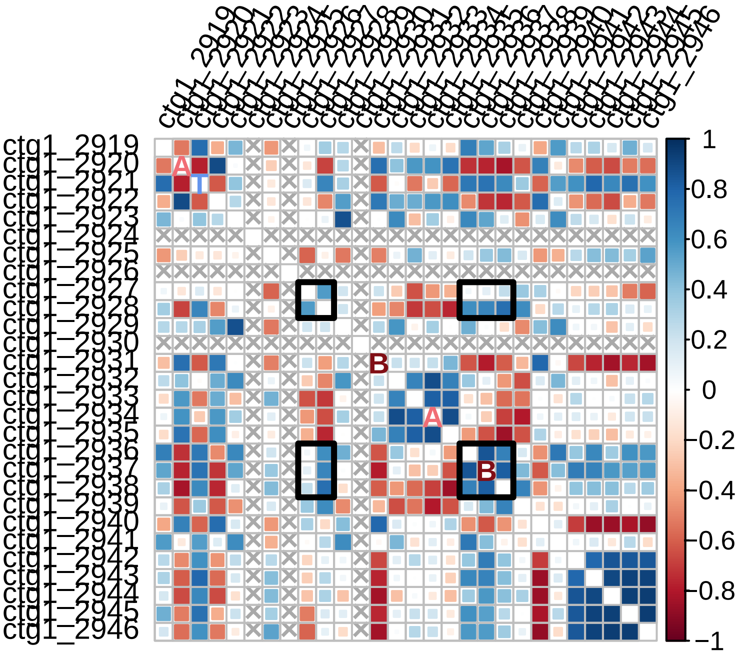

BGC_1.23b

Fj

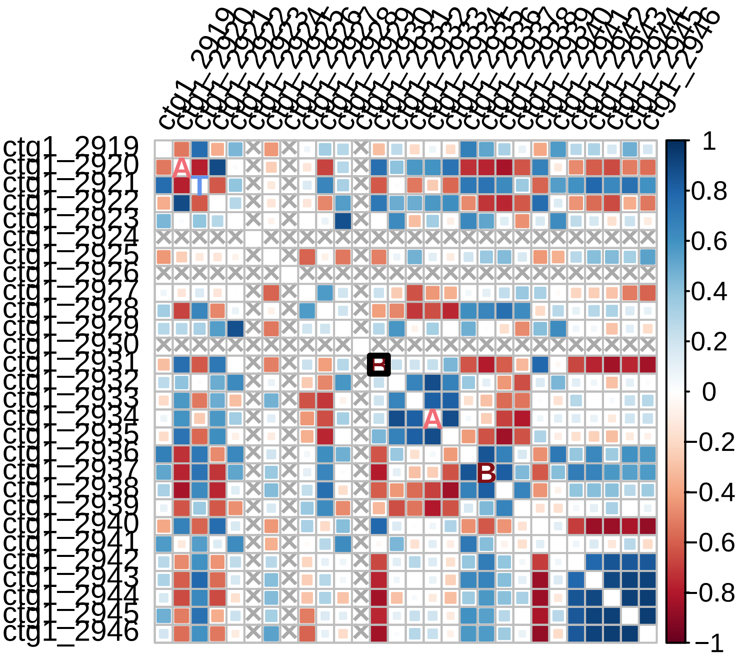

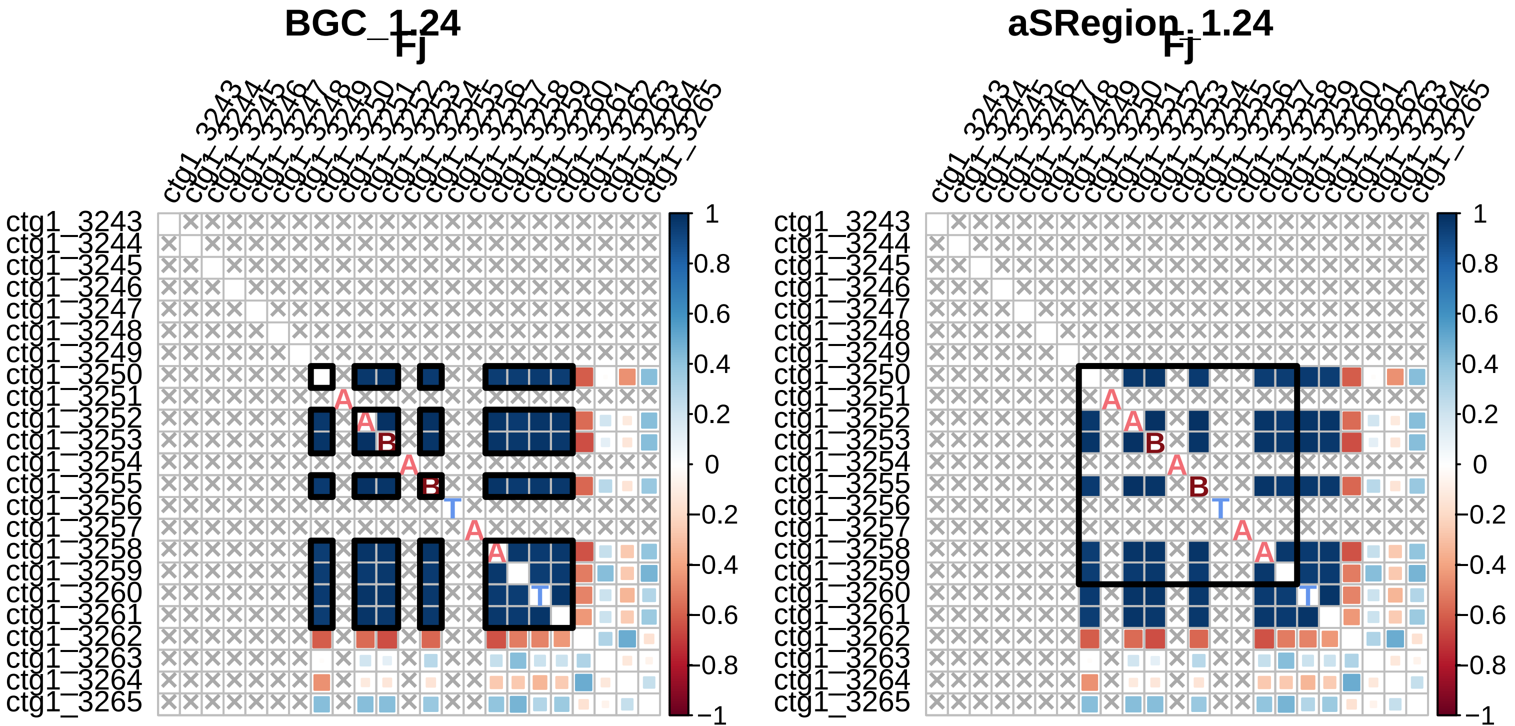

BGC_1.24
Fj
aSRegion_1.24
Fj
ctg1_3243
ctg1_3244
ctg1_3245
ctg1_3246
ctg1_3247
ctg1_3248
ctg1_3249
ctg1_3250
ctg1_3251
ctg1_3252
ctg1_3253
ctg1_3254
ctg1_3255
ctg1_3256
ctg1_3257
ctg1_3258
ctg1_3259
ctg1_3260
ctg1_3261
ctg1_3262
ctg1_3263
ctg1_3264
ctg1_3265
1
0.8
0.6
0.4
0.2
0
−0.2
−0.4
−0.6
−0.8
−1

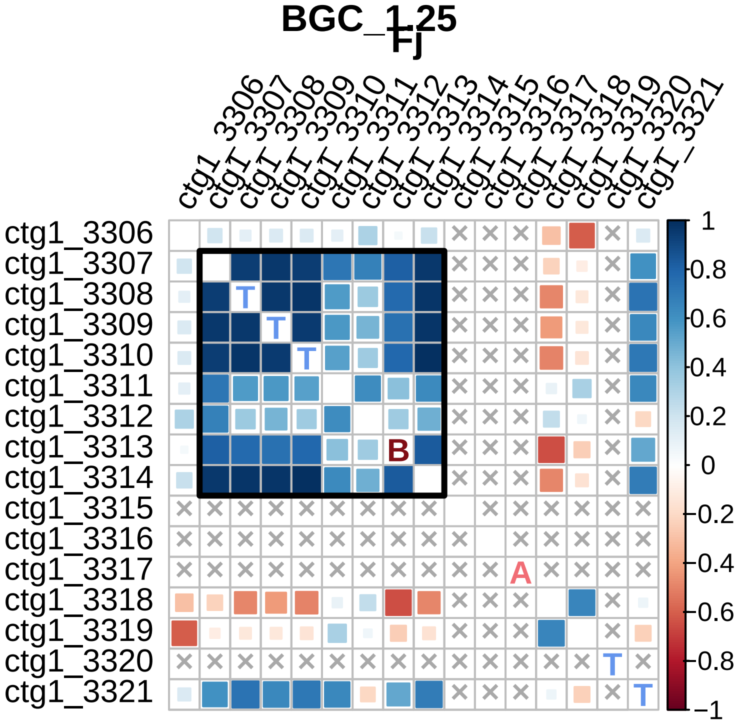

BGC_1.25
Fj
ctg1_3306
ctg1_3307
ctg1_3308
ctg1_3309
ctg1_3310
ctg1_3311
ctg1_3312
ctg1_3313
ctg1_3314
ctg1_3315
ctg1_3316
ctg1_3317
ctg1_3318
ctg1_3319
ctg1_3320
ctg1_3321
T
B
A
1
0.8
0.6
0.4
0.2
0
−0.2
−0.4
−0.6
−0.8
−1

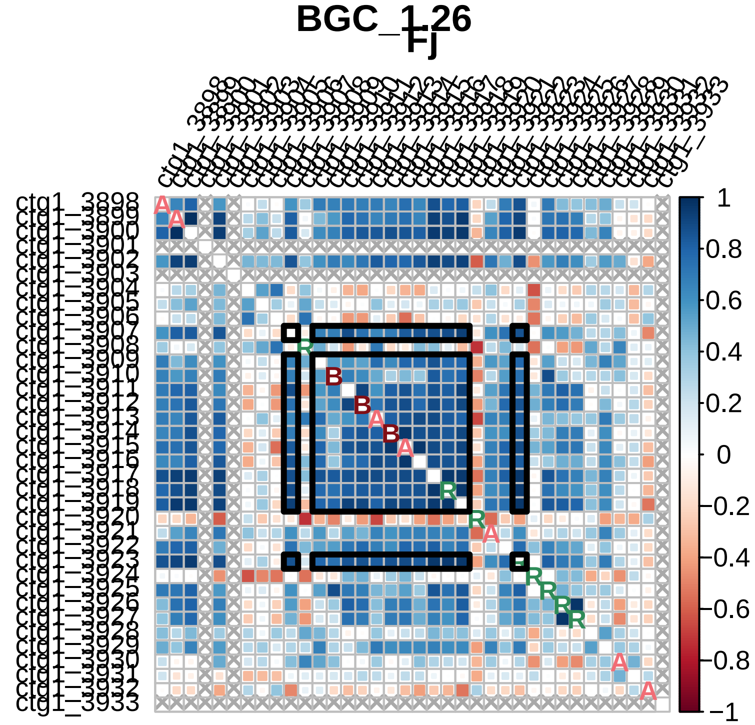
BGC_1_26
Fj
ctg1_3898
ctg1_3933
A
B
R
1
0.8
0.6
0.4
0.2
0
−0.2
−0.4
−0.6
−0.8
−1

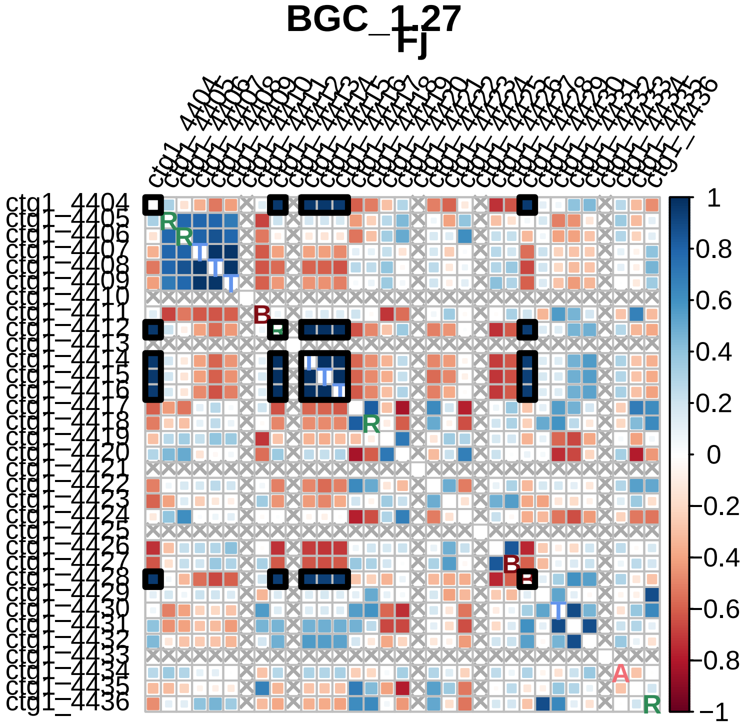

BGC_1_27
Fj
ctg1_4404
ctg1_4405
ctg1_4406
ctg1_4407
ctg1_4408
ctg1_4409
ctg1_4410
ctg1_4411
ctg1_4412
ctg1_4413
ctg1_4414
ctg1_4415
ctg1_4416
ctg1_4417
ctg1_4418
ctg1_4419
ctg1_4420
ctg1_4421
ctg1_4422
ctg1_4423
ctg1_4424
ctg1_4425
ctg1_4426
ctg1_4427
ctg1_4428
ctg1_4429
ctg1_4430
ctg1_4431
ctg1_4432
ctg1_4433
ctg1_4434
ctg1_4435
ctg1_4436
1
0.8
0.6
0.4
0.2
0
−0.2
−0.4
−0.6
−0.8
−1

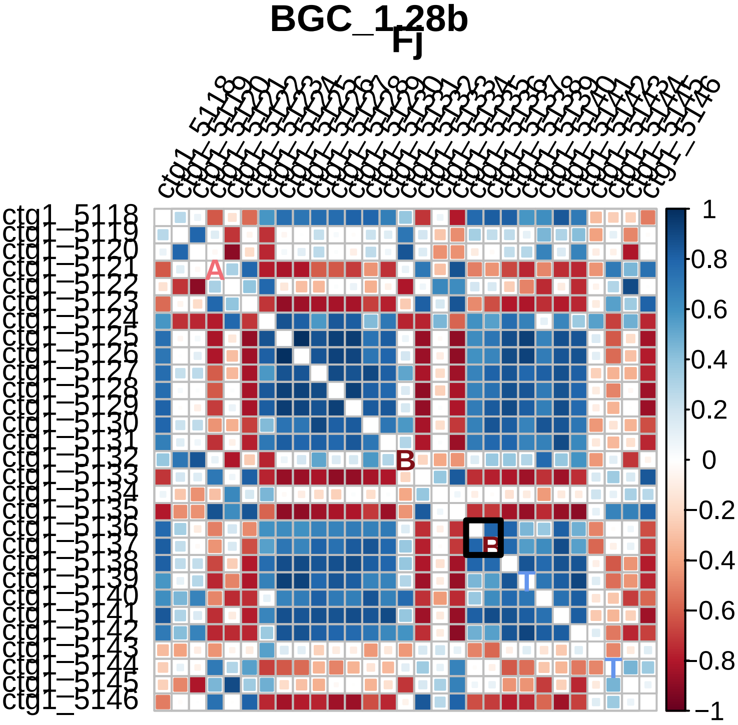
BGC_1.28b
Fj
ctg1_5118
ctg1_5119
ctg1_5120
ctg1_5121
ctg1_5122
ctg1_5123
ctg1_5124
ctg1_5125
ctg1_5126
ctg1_5127
ctg1_5128
ctg1_5129
ctg1_5130
ctg1_5131
ctg1_5132
ctg1_5133
ctg1_5134
ctg1_5135
ctg1_5136
ctg1_5137
ctg1_5138
ctg1_5139
ctg1_5140
ctg1_5141
ctg1_5142
ctg1_5143
ctg1_5144
ctg1_5145
ctg1_5146
A
B
B
T
T
1
0.8
0.6
0.4
0.2
0
−0.2
−0.4
−0.6
−0.8
−1

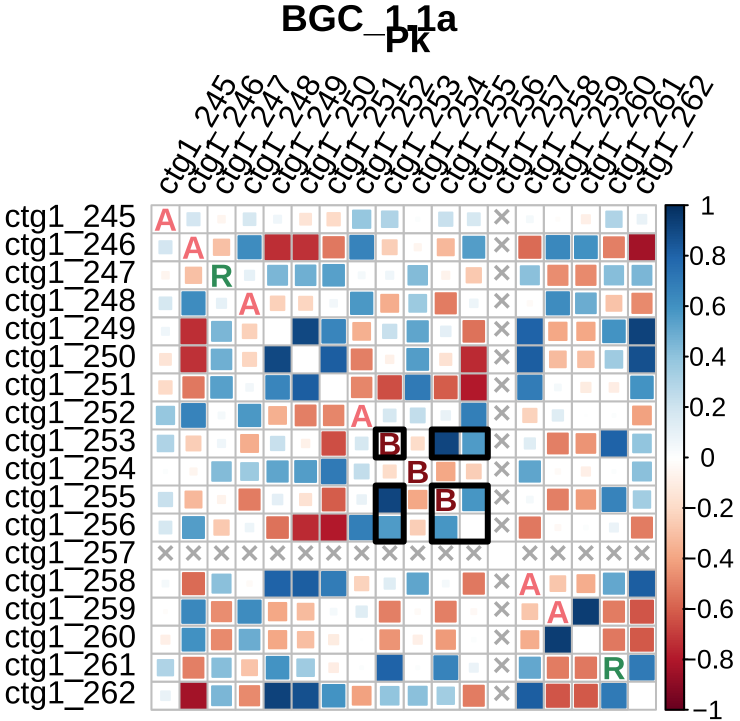

BGC_1.1a
_Pk
ctg1_245
ctg1_246
ctg1_247
ctg1_248
ctg1_249
ctg1_250
ctg1_251
ctg1_252
ctg1_253
ctg1_254
ctg1_255
ctg1_256
ctg1_257
ctg1_258
ctg1_259
ctg1_260
ctg1_261
ctg1_262
1
0.8
0.6
0.4
0.2
0
−0.2
−0.4
−0.6
−0.8
−1

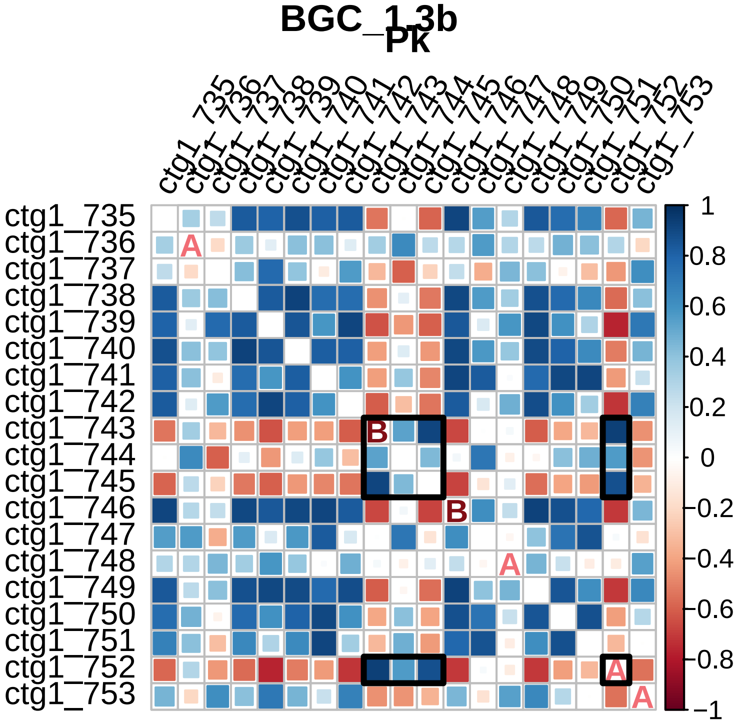
BGC_1.3b
_Pk
ctg1_735
ctg1_736
ctg1_737
ctg1_738
ctg1_739
ctg1_740
ctg1_741
ctg1_742
ctg1_743
ctg1_744
ctg1_745
ctg1_746
ctg1_747
ctg1_748
ctg1_749
ctg1_750
ctg1_751
ctg1_752
ctg1_753
A
B
1
0.8
0.6
0.4
0.2
0
−0.2
−0.4
−0.6
−0.8
−1

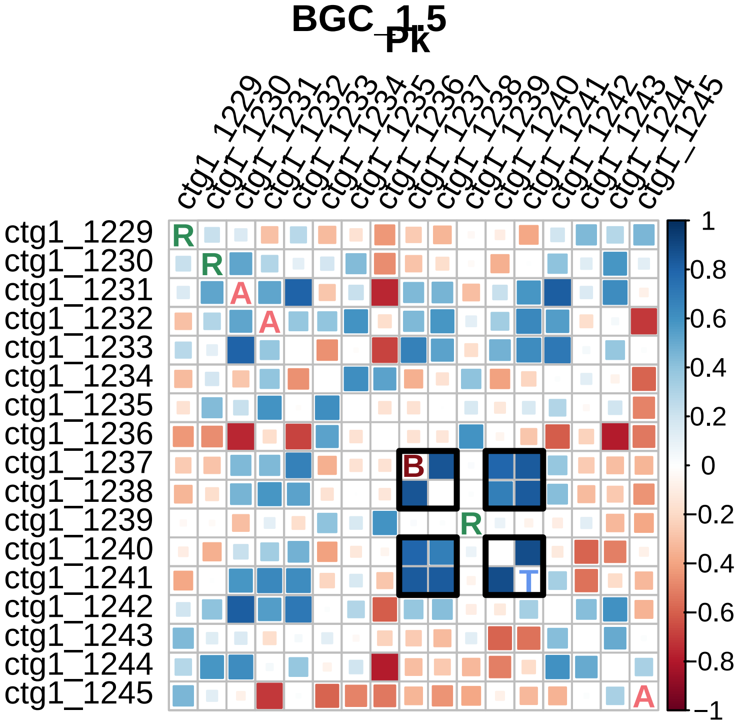

BGC_1.5
_Pk
ctg1_1229
ctg1_1230
ctg1_1231
ctg1_1232
ctg1_1233
ctg1_1234
ctg1_1235
ctg1_1236
ctg1_1237
ctg1_1238
ctg1_1239
ctg1_1240
ctg1_1241
ctg1_1242
ctg1_1243
ctg1_1244
ctg1_1245
R
R
A
A
B
R
T
A
1
0.8
0.6
0.4
0.2
0
−0.2
−0.4
−0.6
−0.8
−1

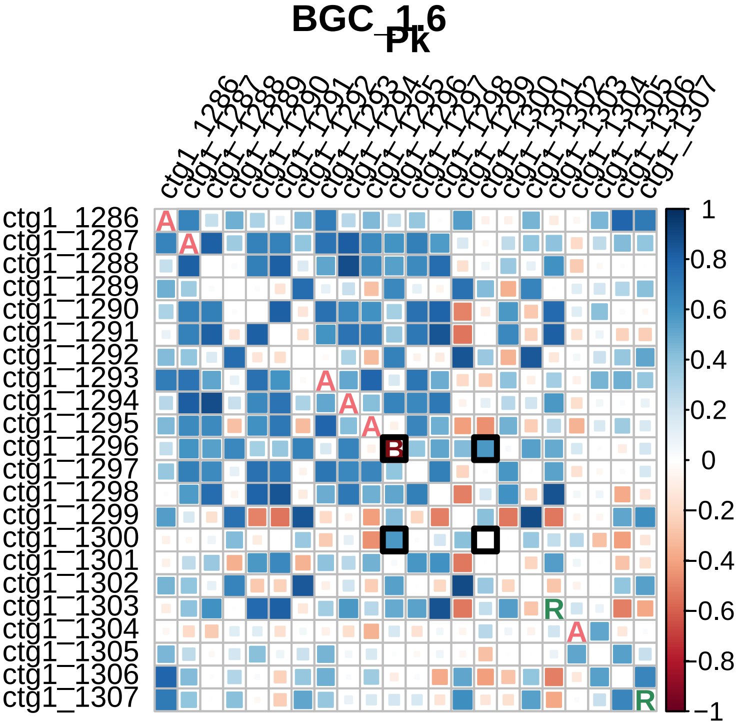
BGC_1.6
Pk
ctg1_1286
ctg1_1287
ctg1_1288
ctg1_1289
ctg1_1290
ctg1_1291
ctg1_1292
ctg1_1293
ctg1_1294
ctg1_1295
ctg1_1296
ctg1_1297
ctg1_1298
ctg1_1299
ctg1_1300
ctg1_1301
ctg1_1302
ctg1_1303
ctg1_1304
ctg1_1305
ctg1_1306
ctg1_1307
A
B
R
1
0.8
0.6
0.4
0.2
0
−0.2
−0.4
−0.6
−0.8
−1

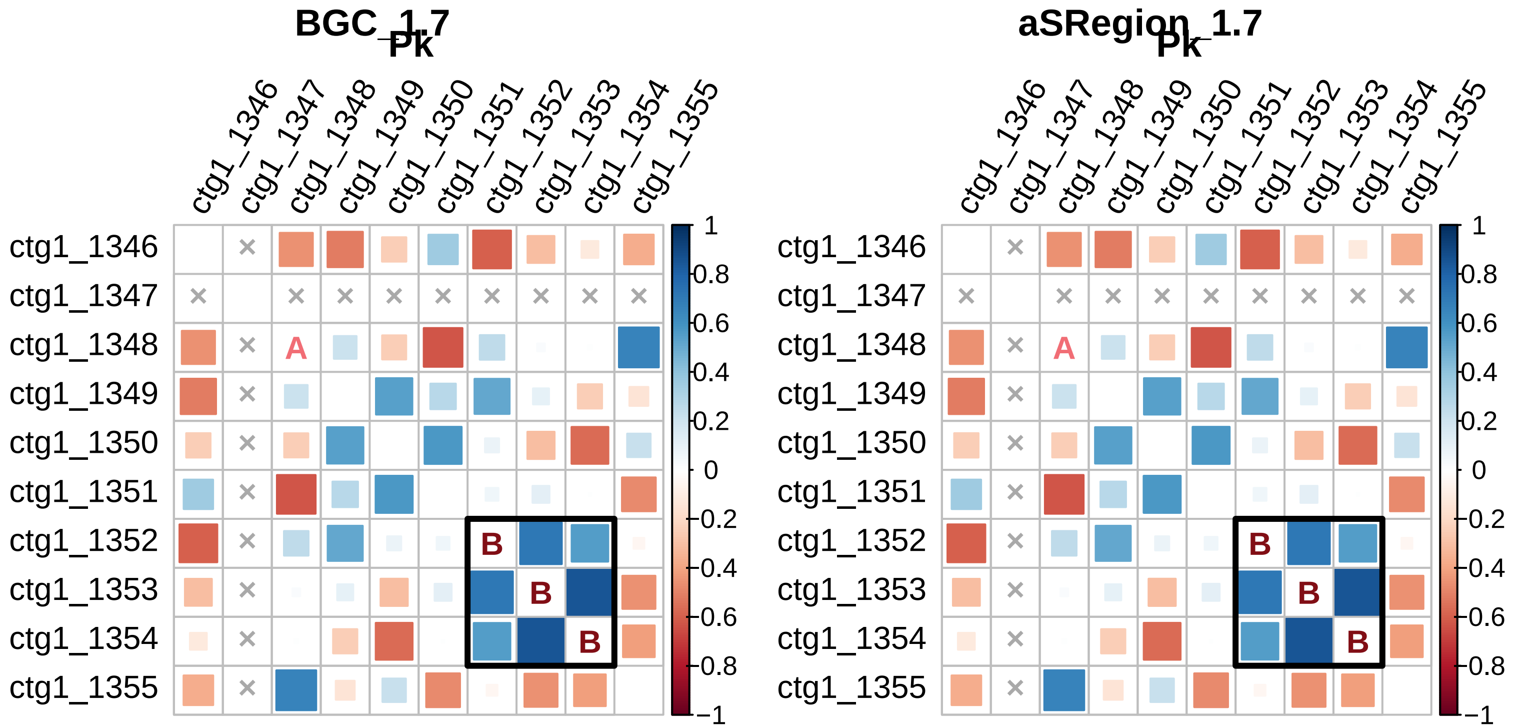

BGC_1.7_Pk
aSRegion_1.7_Pk
ctg1_1346
ctg1_1347
ctg1_1348
ctg1_1349
ctg1_1350
ctg1_1351
ctg1_1352
ctg1_1353
ctg1_1354
ctg1_1355
A
B
1
0.8
0.6
0.4
0.2
0
−0.2
−0.4
−0.6
−0.8
−1

BGC_1.8a

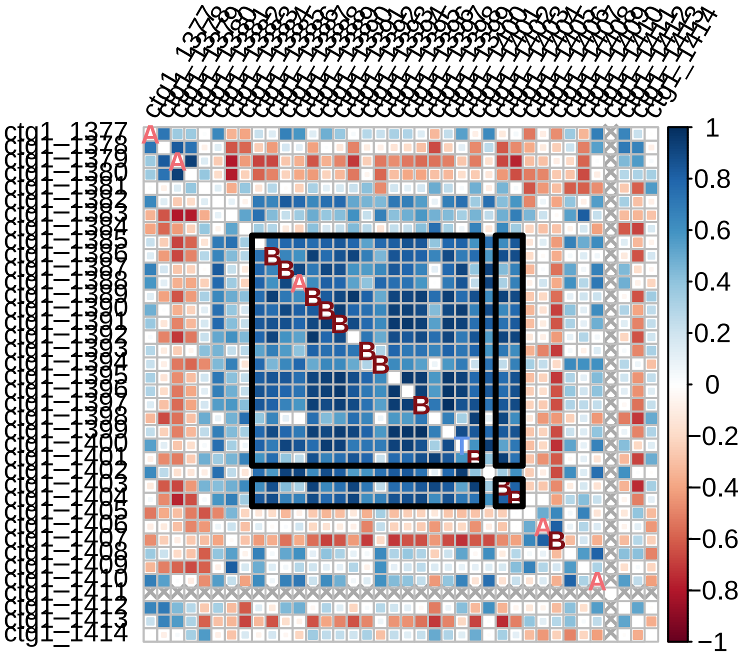

BGC_1.8b
Pk

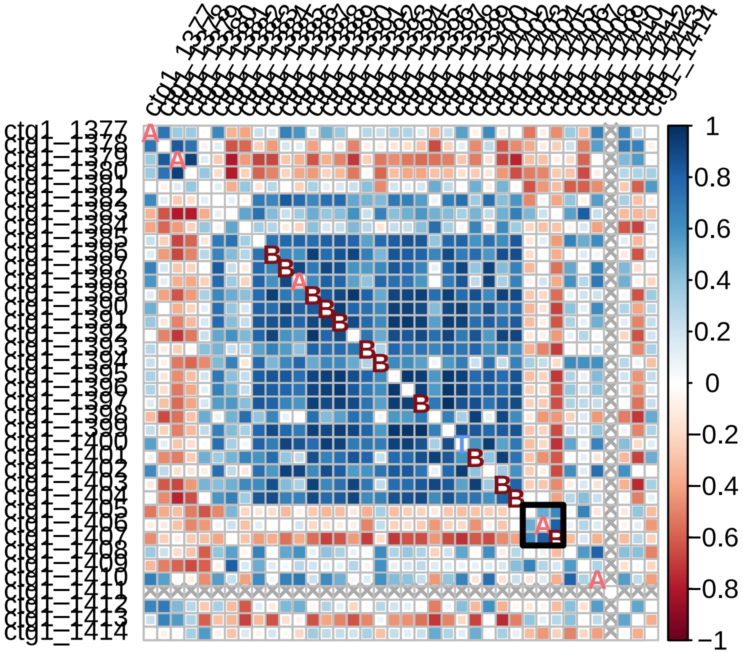

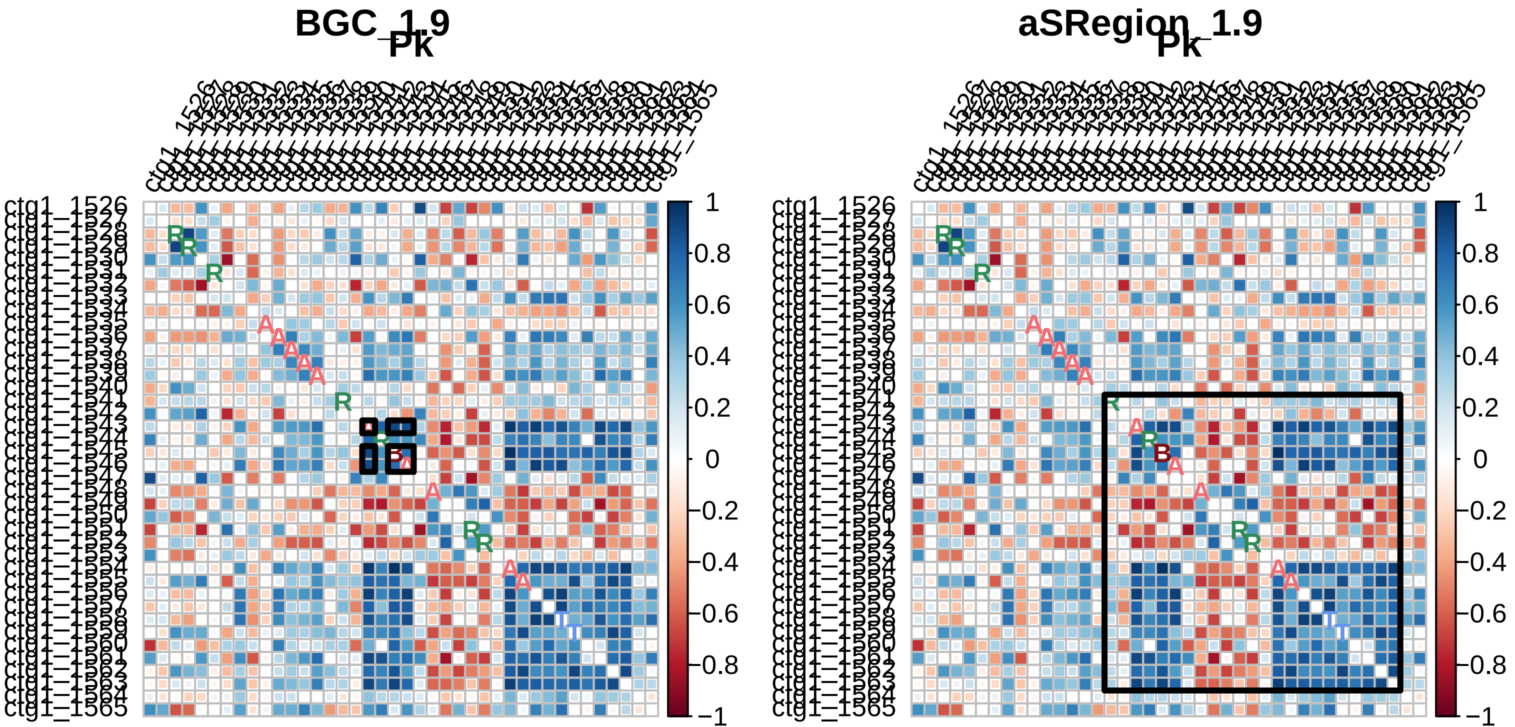

BGC_1.9
Pk
aSRegion_1.9
Pk
ctg1_1526
ctg1_1565
1
0.8
0.6
0.4
0.2
0
-0.2
-0.4
-0.6
-0.8
-1

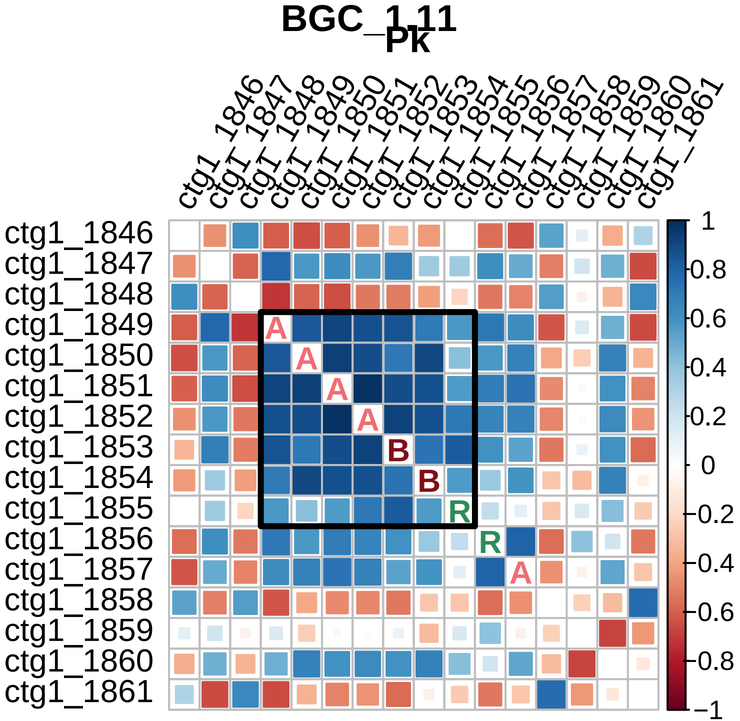

BGC_1.11
_Pk
ctg1_1846
ctg1_1847
ctg1_1848
ctg1_1849
ctg1_1850
ctg1_1851
ctg1_1852
ctg1_1853
ctg1_1854
ctg1_1855
ctg1_1856
ctg1_1857
ctg1_1858
ctg1_1859
ctg1_1860
ctg1_1861
A
A
A
A
B
B
R
R
A
1
0.8
0.6
0.4
0.2
0
−0.2
−0.4
−0.6
−0.8
−1

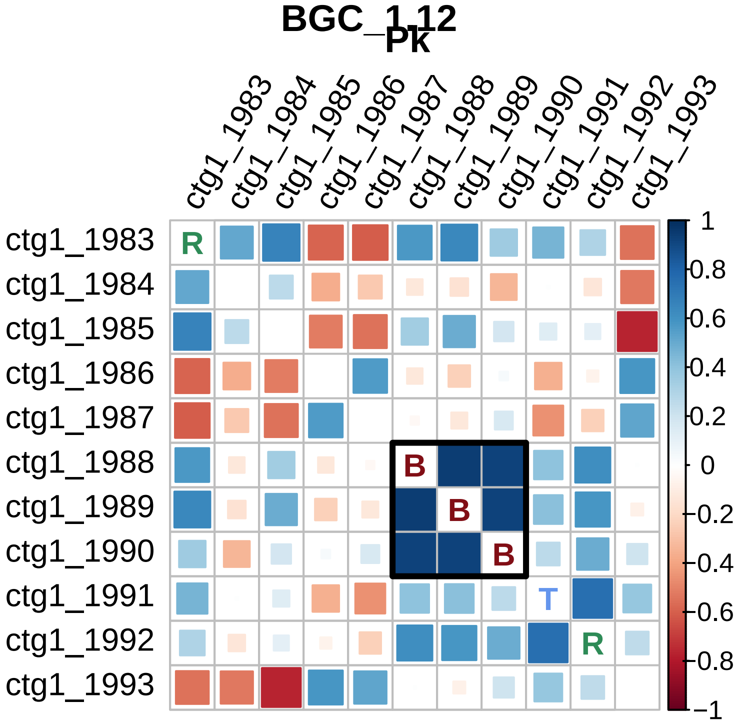
BGC_1.12
Pk
ctg1_1983
ctg1_1984
ctg1_1985
ctg1_1986
ctg1_1987
ctg1_1988
ctg1_1989
ctg1_1990
ctg1_1991
ctg1_1992
ctg1_1993
R
B
B
B
T
R
1
0.8
0.6
0.4
0.2
0
−0.2
−0.4
−0.6
−0.8
−1

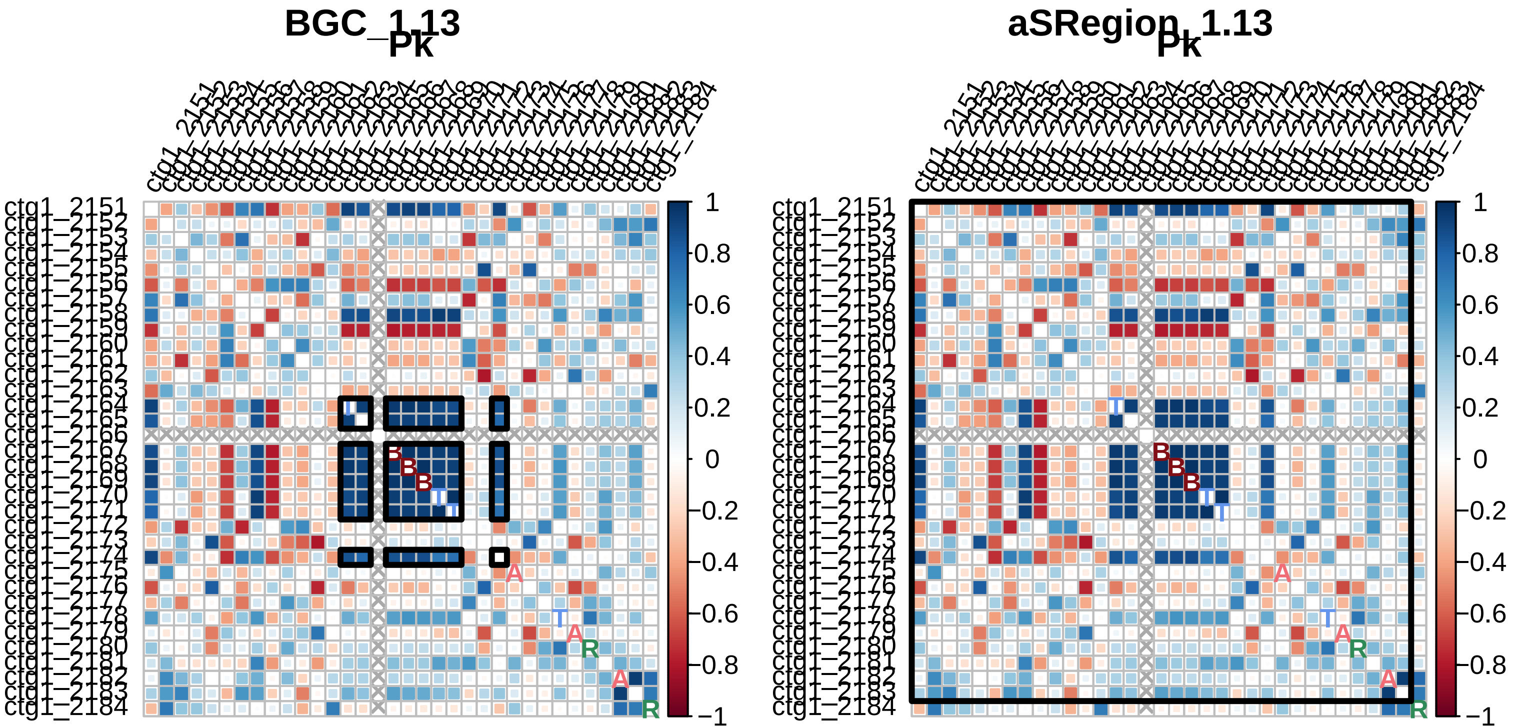
BGC_1.13
Pk
aSRegion_1.13
Pk
ctg1_2151
ctg1_2152
ctg1_2153
ctg1_2154
ctg1_2155
ctg1_2156
ctg1_2157
ctg1_2158
ctg1_2159
ctg1_2160
ctg1_2161
ctg1_2162
ctg1_2163
ctg1_2164
ctg1_2165
ctg1_2166
ctg1_2167
ctg1_2168
ctg1_2169
ctg1_2170
ctg1_2171
ctg1_2172
ctg1_2173
ctg1_2174
ctg1_2175
ctg1_2176
ctg1_2177
ctg1_2178
ctg1_2179
ctg1_2180
ctg1_2181
ctg1_2182
ctg1_2183
ctg1_2184
1
0.8
0.6
0.4
0.2
0
-0.2
-0.4
-0.6
-0.8
-1
B
T
A
R

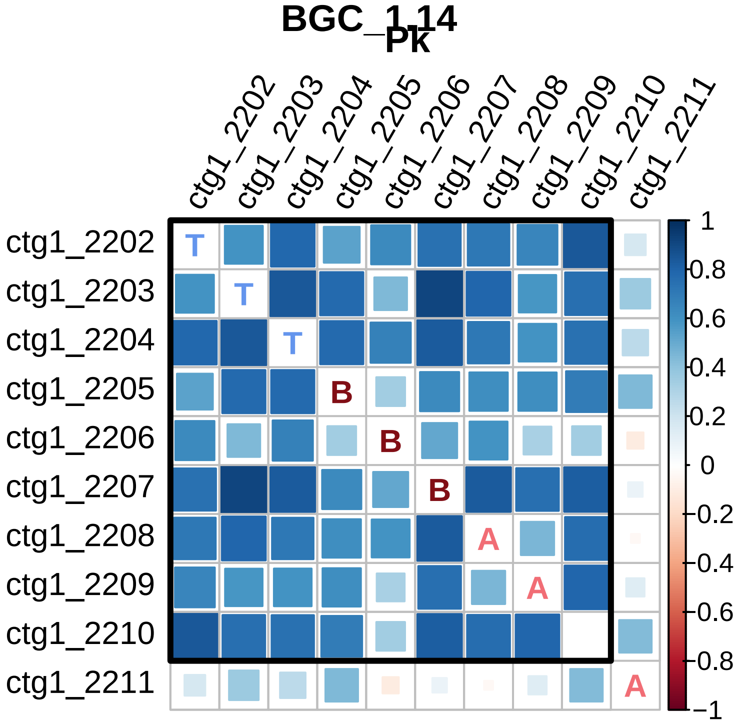
BGC_1_14
Pk
ctg1_2202
ctg1_2203
ctg1_2204
ctg1_2205
ctg1_2206
ctg1_2207
ctg1_2208
ctg1_2209
ctg1_2210
ctg1_2211
T
T
T
B
B
B
A
A
A
1
0.8
0.6
0.4
0.2
0
−0.2
−0.4
−0.6
−0.8
−1

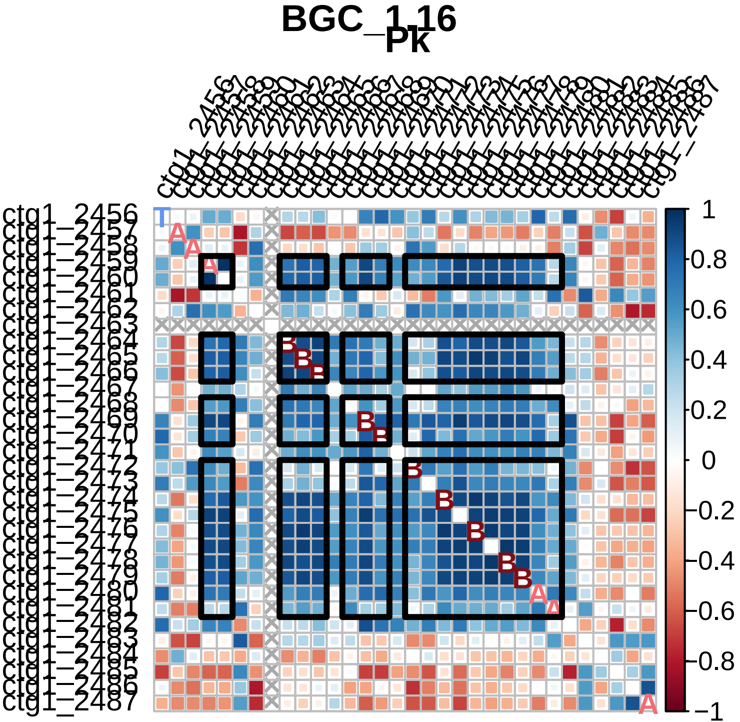
BGC_1_16
Pk
ctg1_2456
ctg1_2457
ctg1_2458
ctg1_2459
ctg1_2460
ctg1_2461
ctg1_2462
ctg1_2463
ctg1_2464
ctg1_2465
ctg1_2466
ctg1_2467
ctg1_2468
ctg1_2469
ctg1_2470
ctg1_2471
ctg1_2472
ctg1_2473
ctg1_2474
ctg1_2475
ctg1_2476
ctg1_2477
ctg1_2478
ctg1_2479
ctg1_2480
ctg1_2481
ctg1_2482
ctg1_2483
ctg1_2484
ctg1_2485
ctg1_2486
ctg1_2487
1
0.8
0.6
0.4
0.2
0
−0.2
−0.4
−0.6
−0.8
−1

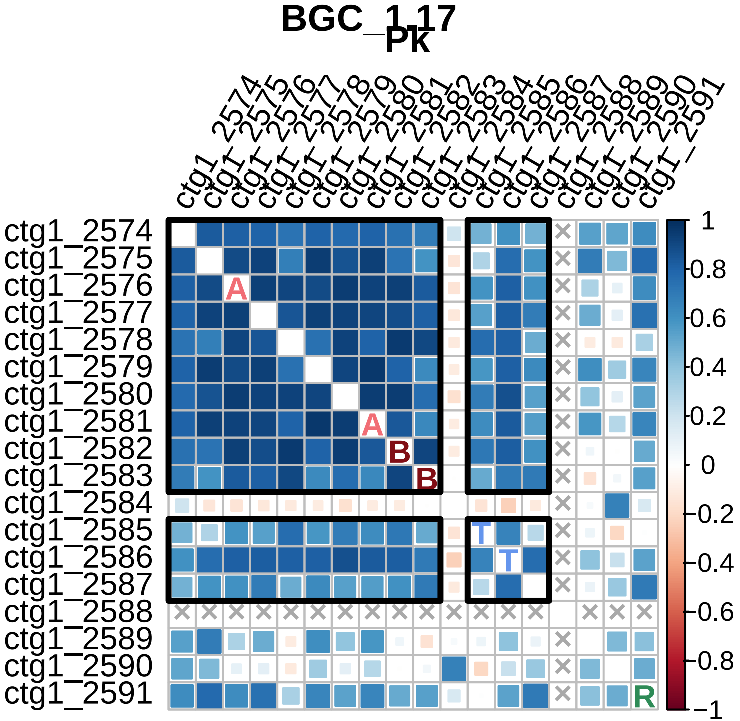

BGC_1_17
_Pk
ctg1_2574
ctg1_2575
ctg1_2576
ctg1_2577
ctg1_2578
ctg1_2579
ctg1_2580
ctg1_2581
ctg1_2582
ctg1_2583
ctg1_2584
ctg1_2585
ctg1_2586
ctg1_2587
ctg1_2588
ctg1_2589
ctg1_2590
ctg1_2591
A
A
B
B
T
T
R
1
0.8
0.6
0.4
0.2
0
−0.2
−0.4
−0.6
−0.8
−1

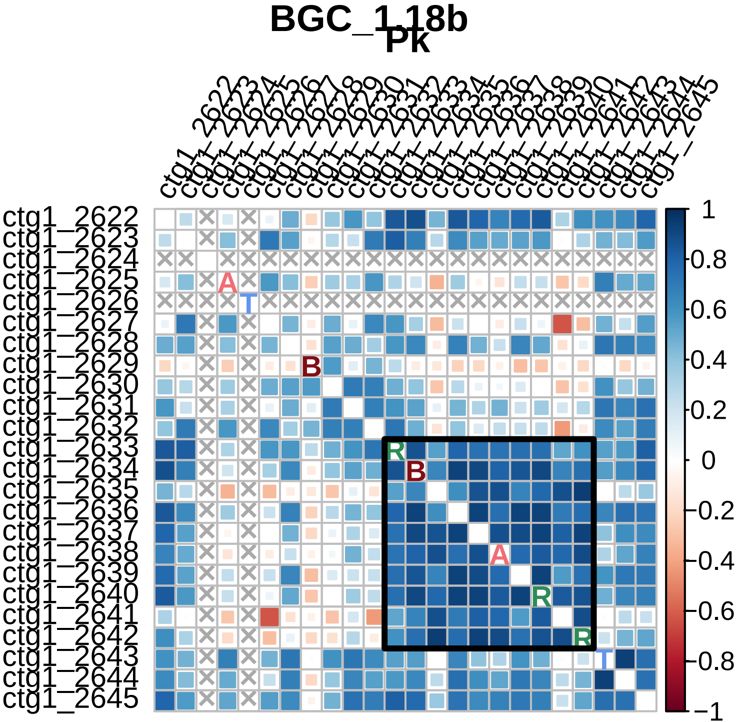

BGC_1.18b
Pk
ctg1_2622
ctg1_2623
ctg1_2624
ctg1_2625
ctg1_2626
ctg1_2627
ctg1_2628
ctg1_2629
ctg1_2630
ctg1_2631
ctg1_2632
ctg1_2633
ctg1_2634
ctg1_2635
ctg1_2636
ctg1_2637
ctg1_2638
ctg1_2639
ctg1_2640
ctg1_2641
ctg1_2642
ctg1_2643
ctg1_2644
ctg1_2645
A
T
B
R
B
A
R
R
T
1
0.8
0.6
0.4
0.2
0
−0.2
−0.4
−0.6
−0.8
−1

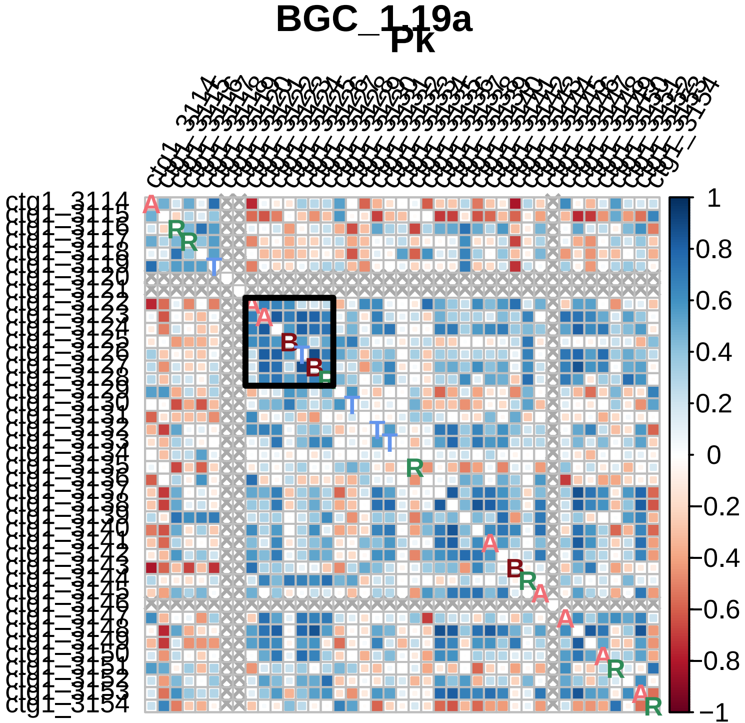

BGC_1.19a
_Pk
ctg1_3114
ctg1_3154
1
0.8
0.6
0.4
0.2
0
−0.2
−0.4
−0.6
−0.8
−1

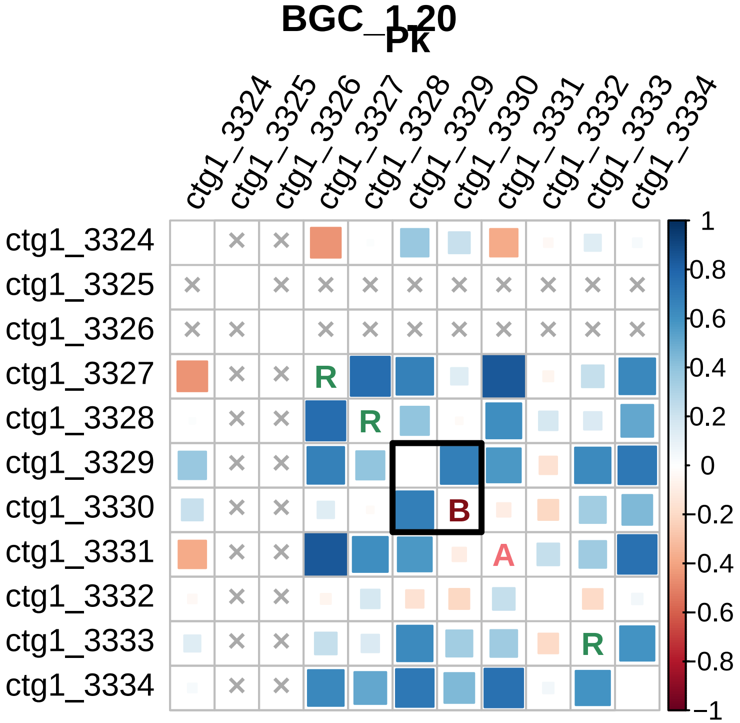
BGC_1.20
_PK
ctg1_3324
ctg1_3325
ctg1_3326
ctg1_3327
ctg1_3328
ctg1_3329
ctg1_3330
ctg1_3331
ctg1_3332
ctg1_3333
ctg1_3334
R
R
B
A
R
1
0.8
0.6
0.4
0.2
0
−0.2
−0.4
−0.6
−0.8
−1

BGC_1.21b
Pk

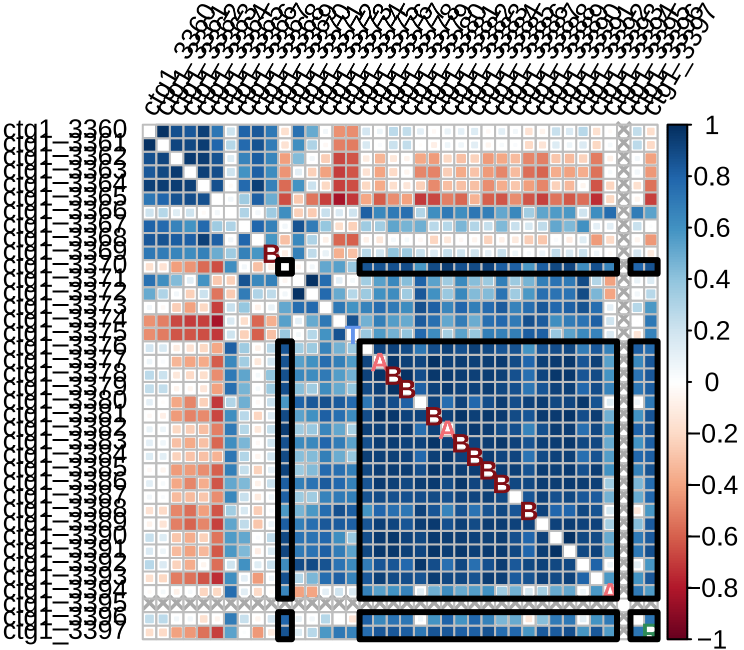

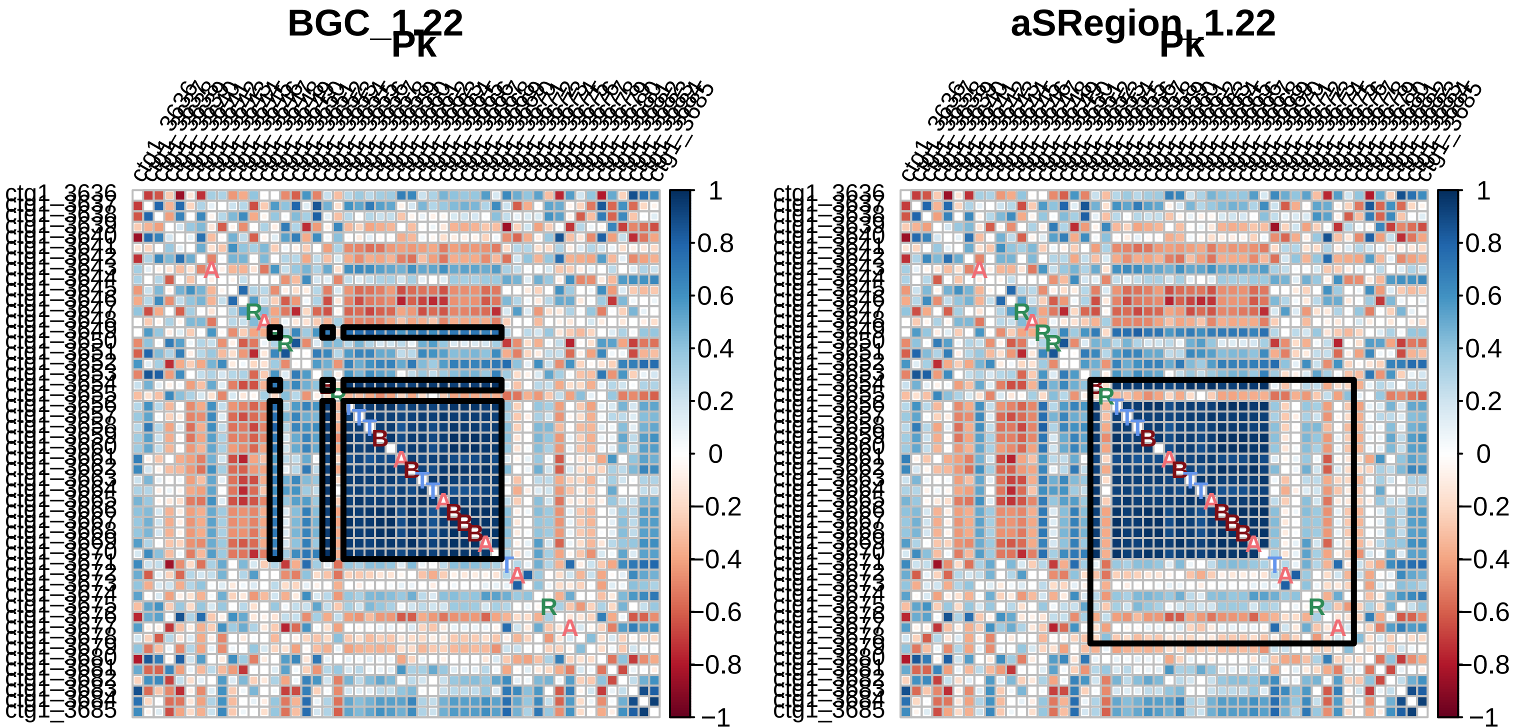
BGC_1.22
Pk
aSRegion_1.22
Pk
ctg1_3636
ctg1_3685
1
0.8
0.6
0.4
0.2
0
−0.2
−0.4
−0.6
−0.8
−1

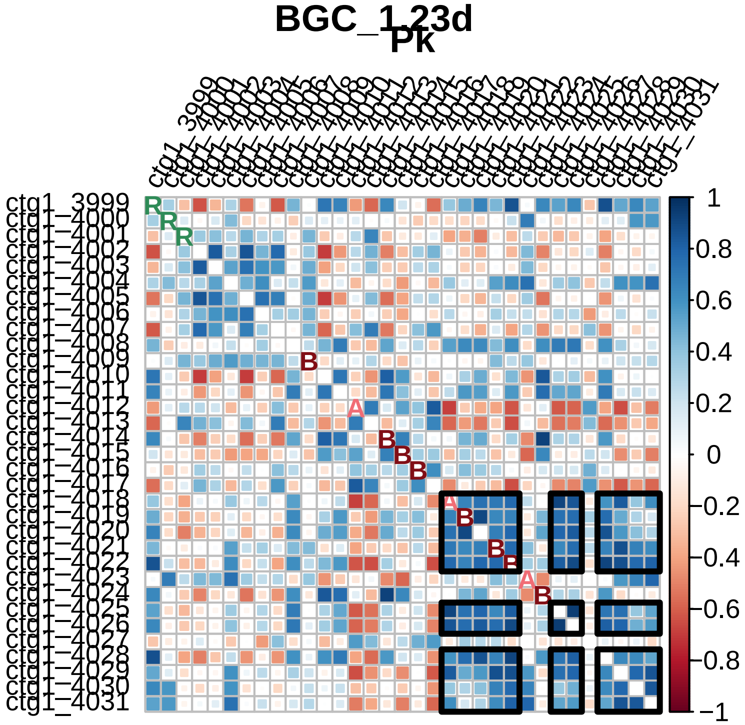

BGC_1.23d
Pk
ctg1_3999
ctg1_4000
ctg1_4001
ctg1_4002
ctg1_4003
ctg1_4004
ctg1_4005
ctg1_4006
ctg1_4007
ctg1_4008
ctg1_4009
ctg1_4010
ctg1_4011
ctg1_4012
ctg1_4013
ctg1_4014
ctg1_4015
ctg1_4016
ctg1_4017
ctg1_4018
ctg1_4019
ctg1_4020
ctg1_4021
ctg1_4022
ctg1_4023
ctg1_4024
ctg1_4025
ctg1_4026
ctg1_4027
ctg1_4028
ctg1_4029
ctg1_4030
ctg1_4031
1
0.8
0.6
0.4
0.2
0
−0.2
−0.4
−0.6
−0.8
−1

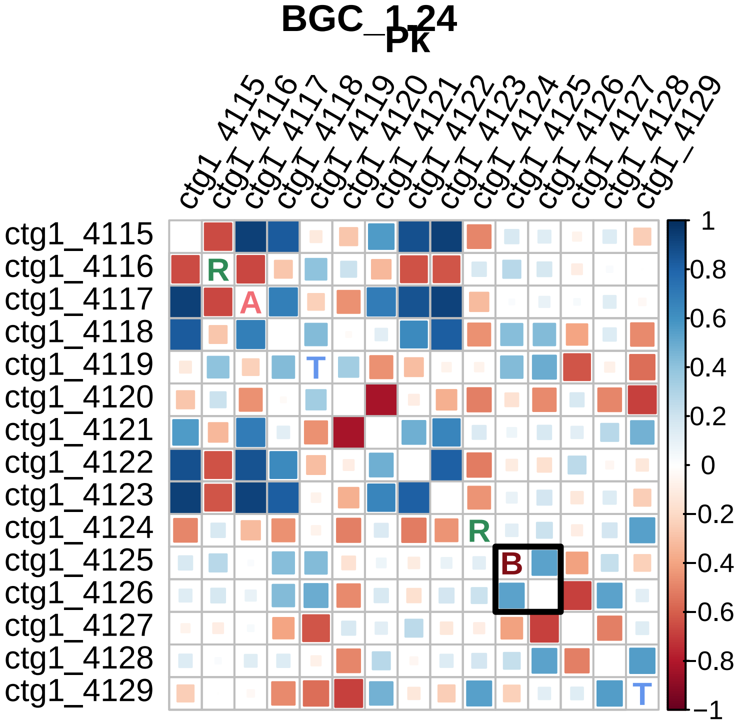

BGC_1_24
_PK
ctg1_4115
ctg1_4116
ctg1_4117
ctg1_4118
ctg1_4119
ctg1_4120
ctg1_4121
ctg1_4122
ctg1_4123
ctg1_4124
ctg1_4125
ctg1_4126
ctg1_4127
ctg1_4128
ctg1_4129
R
A
T
R
B
T
1
0.8
0.6
0.4
0.2
0
−0.2
−0.4
−0.6
−0.8
−1

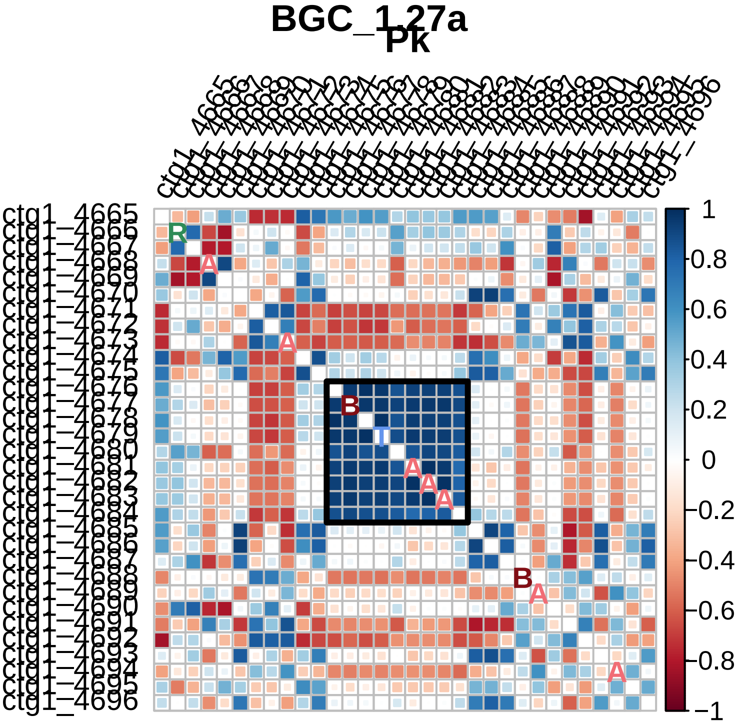
BGC_1_27a
Pk
ctg1_4665
ctg1_4666
ctg1_4667
ctg1_4668
ctg1_4669
ctg1_4670
ctg1_4671
ctg1_4672
ctg1_4673
ctg1_4674
ctg1_4675
ctg1_4676
ctg1_4677
ctg1_4678
ctg1_4679
ctg1_4680
ctg1_4681
ctg1_4682
ctg1_4683
ctg1_4684
ctg1_4685
ctg1_4686
ctg1_4687
ctg1_4688
ctg1_4689
ctg1_4690
ctg1_4691
ctg1_4692
ctg1_4693
ctg1_4694
ctg1_4695
ctg1_4696
R
A
A
B
T
A
A
A
B
A
A
1
0.8
0.6
0.4
0.2
0
−0.2
−0.4
−0.6
−0.8
−1

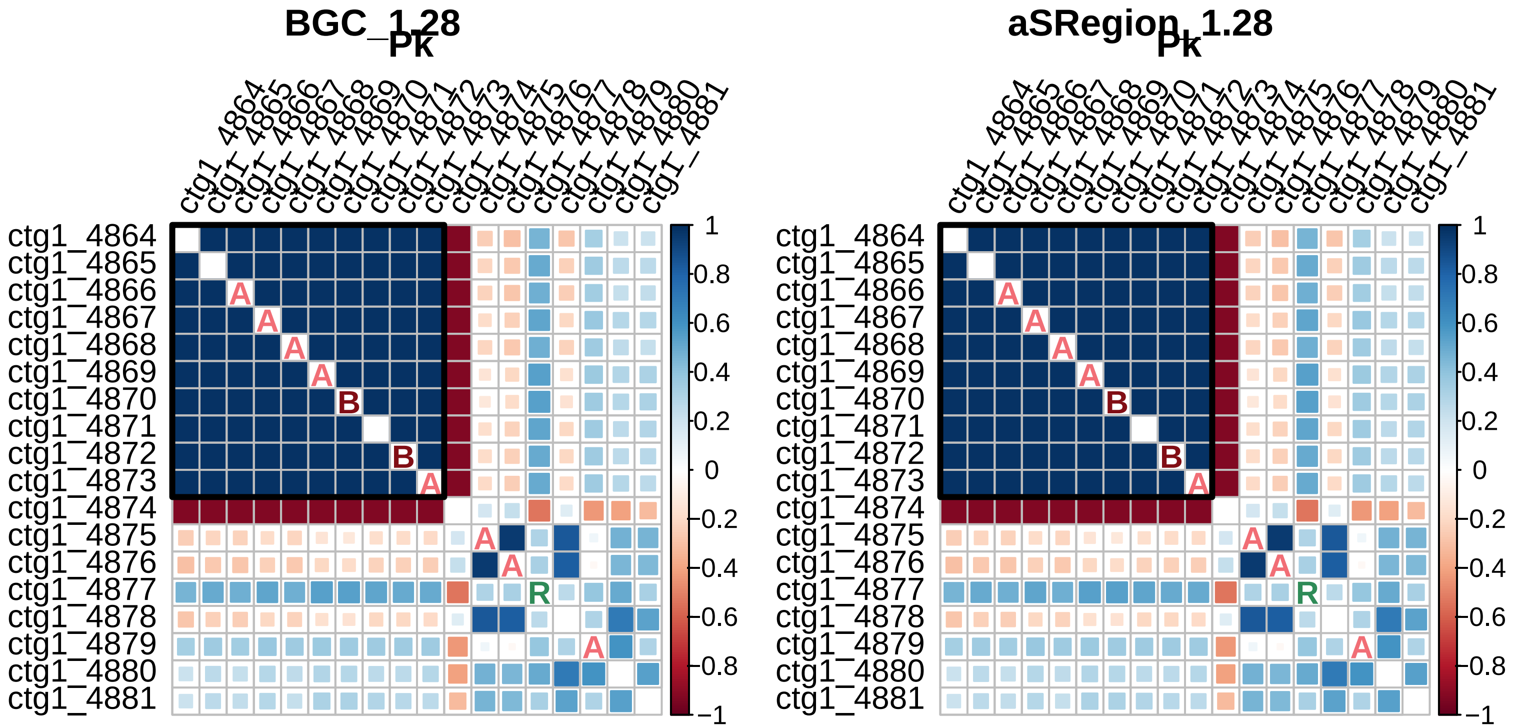
BGC_1.28
Pk
aSRegion_1.28
Pk
ctg1_4864
ctg1_4865
ctg1_4866
ctg1_4867
ctg1_4868
ctg1_4869
ctg1_4870
ctg1_4871
ctg1_4872
ctg1_4873
ctg1_4874
ctg1_4875
ctg1_4876
ctg1_4877
ctg1_4878
ctg1_4879
ctg1_4880
ctg1_4881
1
0.8
0.6
0.4
0.2
0
−0.2
−0.4
−0.6
−0.8
−1
A
B
R

BGC_1.30a

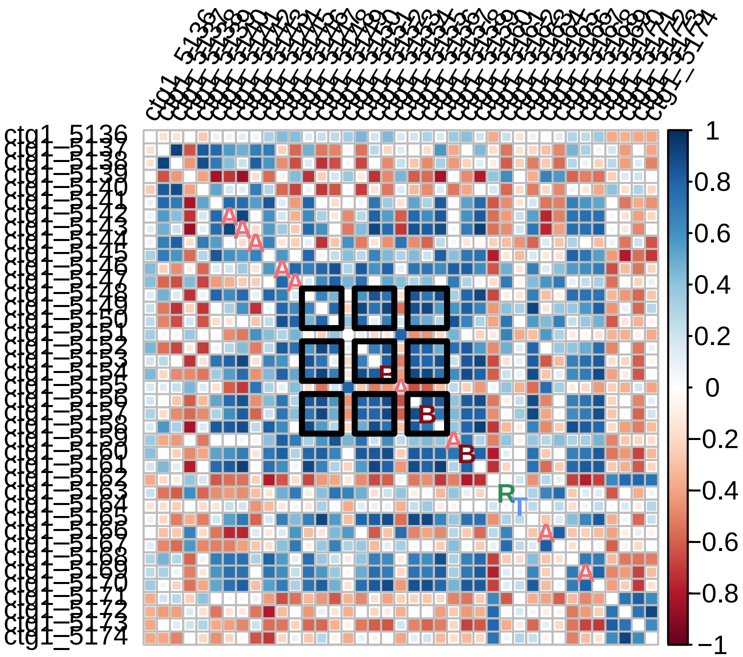

BGC_1.30b
Pk

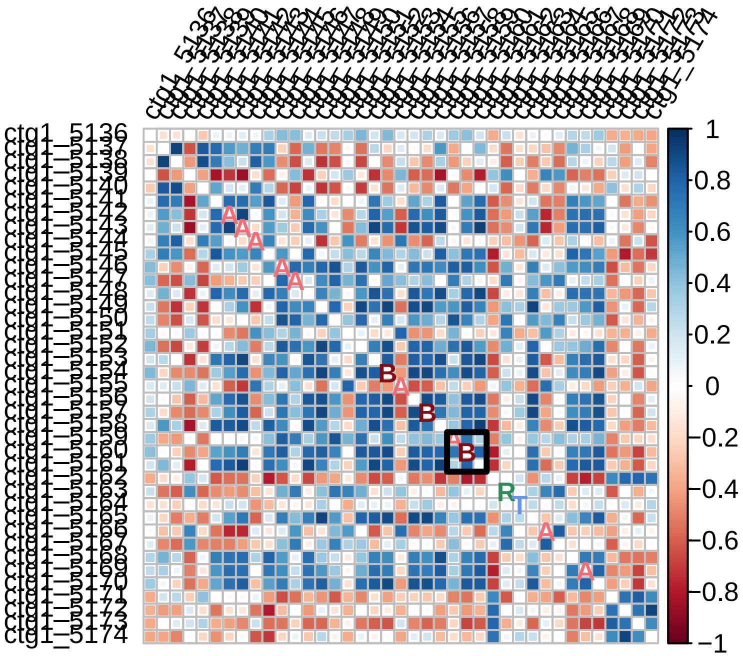

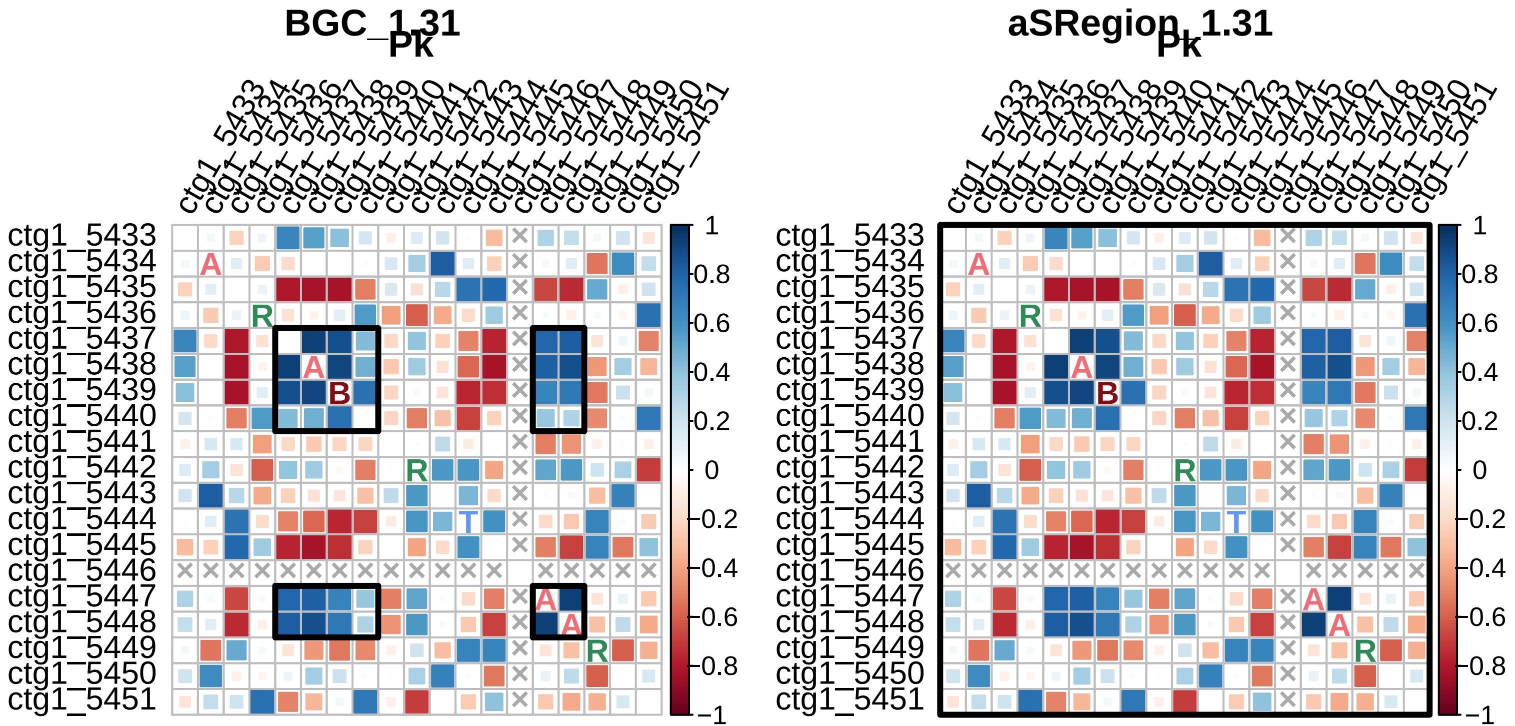
BGC_1.31
Pk
aSRegion_1.31
Pk
ctg1_5433
ctg1_5434
ctg1_5435
ctg1_5436
ctg1_5437
ctg1_5438
ctg1_5439
ctg1_5440
ctg1_5441
ctg1_5442
ctg1_5443
ctg1_5444
ctg1_5445
ctg1_5446
ctg1_5447
ctg1_5448
ctg1_5449
ctg1_5450
ctg1_5451
A
R
B
T
1
0.8
0.6
0.4
0.2
0
−0.2
−0.4
−0.6
−0.8
−1

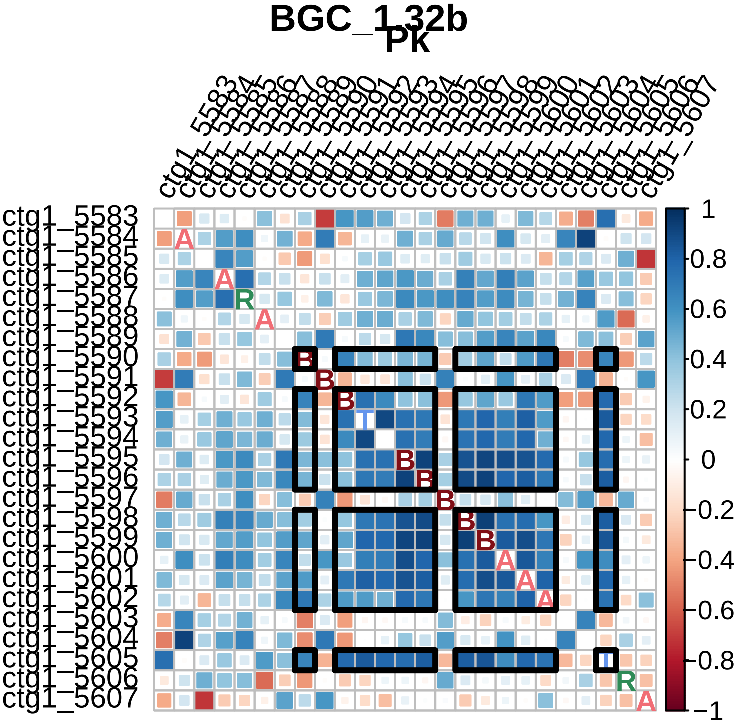
BGC_1.32b
Pk
ctg1_5583
ctg1_5584
ctg1_5585
ctg1_5586
ctg1_5587
ctg1_5588
ctg1_5589
ctg1_5590
ctg1_5591
ctg1_5592
ctg1_5593
ctg1_5594
ctg1_5595
ctg1_5596
ctg1_5597
ctg1_5598
ctg1_5599
ctg1_5600
ctg1_5601
ctg1_5602
ctg1_5603
ctg1_5604
ctg1_5605
ctg1_5606
ctg1_5607
1
0.8
0.6
0.4
0.2
0
−0.2
−0.4
−0.6
−0.8
−1
